# Selected Pharmaceuticals in Different Aquatic Compartments: Part I—Source, Fate and Occurrence

**DOI:** 10.3390/molecules25051026

**Published:** 2020-02-25

**Authors:** André Pereira, Liliana Silva, Célia Laranjeiro, Celeste Lino, Angelina Pena

**Affiliations:** LAQV, REQUIMTE, Laboratory of Bromatology and Pharmacognosy, Faculty of Pharmacy, University of Coimbra, Polo III, Azinhaga de Stª Comba, Coimbra, 3000-548, Portugal; ljgsilva@hotmail.com (L.S.); celialaranjeiro@gmail.com (C.L.); cmlino@ci.uc.pt (C.L.); apena@ci.uc.pt (A.P.)

**Keywords:** environmental contaminants, pharmaceuticals occurrence, pharmaceuticals, aquatic compartments

## Abstract

Potential risks associated with releases of human pharmaceuticals into the environment have become an increasingly important issue in environmental health. This concern has been driven by the widespread detection of pharmaceuticals in all aquatic compartments. Therefore, 22 pharmaceuticals, 6 metabolites and transformation products, belonging to 7 therapeutic groups, were selected to perform a systematic review on their source, fate and occurrence in different aquatic compartments, important issues to tackle the Water Framework Directive (WFD). The results obtained evidence that concentrations of pharmaceuticals are present, in decreasing order, in wastewater influents (WWIs), wastewater effluents (WWEs) and surface waters, with values up to 14 mg L^−1^ for ibuprofen in WWIs. The therapeutic groups which presented higher detection frequencies and concentrations were anti-inflammatories, antiepileptics, antibiotics and lipid regulators. These results present a broad and specialized background, enabling a complete overview on the occurrence of pharmaceuticals in the aquatic compartments.

## 1. Introduction

Human pharmaceuticals, presenting different characteristics and, consequently, producing different environmental exposure profiles, represent a group of widely used chemicals that contaminate the aquatic environment. Albeit in trace amounts, they are of concern, since they are designed to perform a biological effect. Moreover, given their continuous introduction into the environment, their impact, both as stressors and as agents of change, is of great importance [1].

The main source of pharmaceuticals residues in the aquatic environment is human excretion, and consequently, the widespread presence of pharmaceuticals in environmental samples is most likely to occur from wastewater treatment plants (WWTPs), which incompletely remove these compounds. Pharmaceuticals are then released into the environment as parent compounds, metabolites, as well as transformation products [2], leading to the contamination of surface waters, seawaters, groundwater and even some drinking waters already identified by new analytical methodologies which allowed the detection at low ng L^−1^ [3,4,5,6,7,8,9,10].

Although no legal limits have been established in water, seven pharmaceuticals and one metabolite became part of the WFD watch list established by the Directive 2013/39/EU amended by the Commission Implementing Decision from the EU 2015/495 and the EU 2018/840. This list is dynamic, changing with the awareness on the persistence in the water cycle, and its validity in time is limited. Therefore, identifying and prioritizing new pharmaceuticals are important goals to be accomplished for future updates in order to minimize the aquatic environmental contamination by pharmaceuticals [11]. Additionally, as a part of the strategy implemented by the Directive 2013/39/EU, all member states shall monitor the substances in the watch list at the selected surface waters’ representative monitoring stations.

Globally, heavy contamination pressures from extensive urban activities characterize the main rivers that might lead to high aquatic contamination levels and consequent environmental and human exposure. Although the concentrations of pharmaceuticals in influents (WWIs) and effluents (WWEs) of WWTPs and surface waters are routinely monitored in many countries, only in recent years there has been an increase in the number of studies concerning the occurrence of pharmaceuticals in the aquatic environment [12,13,14,15,16]. Additionally, other aquatic compartments such as seawater, groundwater, mineral water and drinking water have a lower amount of data available regarding this contamination. However, most of these studies are primarily focused on a small number of targeted compounds in localized areas. Therefore, there is a knowledge gap which demands a comprehensive and systematic evaluation of pharmaceuticals, its metabolites and transformation products in the aquatic environment.

Thus, a systematic review, in order to provide a clear insight on pharmaceuticals’ contamination of the water compartment, should embrace, not only several parent compounds, but also metabolites and transformations products belonging to different therapeutic groups (Table 1).

The pharmaceuticals in study, key representatives of major classes of pharmaceuticals, were selected based on the EU watch list, their high consumption, pharmacokinetics, physicochemical properties, persistence, previous studies on the occurrence on the aquatic environment and their potential toxicological impact, both on humans and on the aquatic environment [11,17,18,19,20]. In this way, the complete scenario of the contamination of pharmaceuticals in the aquatic environment could be acquired, contributing to future improvements in minimization measures, calculation of the environmental risk assessment and legislation.

In a larger vision of future water resource management sustainability, with the escalating population growth and intensified agricultural and industrial activity, water scarcity will be a reality [21,22,23]. Therefore, there will be the need for water/wastewater recycling, and the contamination of water resources by pharmaceuticals gains yet another perspective. Therefore, it is important to obtain a better understanding of the context, concerning the source, fate and occurrence posed by pharmaceuticals in the aquatic environment.

## 2. Sources and Fate of Pharmaceuticals in the Environment

### 2.1. Sources

Pharmaceuticals are widely consumed throughout the world and can reach the aquatic environment, primarily through human excretion or by direct disposal of unused or expired drugs in toilets, being WWTPs are considered the primary sources of these contaminants into the water bodies [18,24]. Although they are administered within healthcare facilities, namely, hospitals, nursing, assisted living and independent living healthcare facilities, its contribution to the input of pharmaceuticals into the municipal WWTPs is quite low, since these facilities typically make a small contribution to the overall load [3,25,26]. The hospital contribution to the total load of pharmaceuticals in municipal WWTPs is for most compounds under 10% and, usually, even below 3% [9]. However, wastewaters from drug production can be a potential source of pharmaceuticals in certain locations, namely, in major production areas for the global bulk drug market [6]. Finally, veterinary medicines can also enter the environment; however, their environmental exposure routes and fate differ from human pharmaceuticals [19,27].

Thus, these drugs, their metabolites and/or transformation products may enter the environment via WWTPs effluents or by land application of biosolids, originating from WWTPs sludges, which, through runoff or leaching, can enter the aquatic environment, surface or groundwaters [28]. It is important to highlight that the EU banned disposal of sewage sludge at sea in 1998, and since then, its application rate to land has risen significantly [29].

### 2.2. Consumption Patterns

The presence of pharmaceuticals in the environment generally correlates well with the amount used in human medicine. Therefore, these data can be used to identify pharmaceuticals that may pose a risk to the environment [30]. An accurate estimate of the extent of drug exposure in a population is difficult in most countries, as precise consumption data are often lacking. In addition, the statistics frequently cover prescription drugs only and do not include over-the-counter medicines or hospital use of pharmaceuticals [31].

Nevertheless, for several reasons, consumption of pharmaceuticals is expected to increase and, thus, increase the burden of their presence in the environment. First, as the number of older people is rising, with frequent therapeutic regimes of five or more medicines, the extensive use of pharmaceuticals will also increase. In addition, with a rise in living standards and with a decrease in pharmaceuticals price, their usage will escalate throughout the world [9].

Bearing in mind the available data on antidepressants and lipid regulators provided by the Organization for Economic Cooperation and Development (OECD), in defined daily dose (DDD), which is calculated per 1000 inhabitants per day, the increased consumption from 2000 to 2015 is clear, with an increase of 30.7 to 60.6 DDD and of 28.1 to 100.7 DDD in antidepressants and lipid regulators, respectively [32].

However, the correlation between consumption data and environmental contamination is related to the amount consumed per year (kg y^−1^), which may not correspond to a higher DDD, that varies widely between pharmaceuticals. For example, in 2000, approximately 100 million women worldwide were current users of combined hormonal contraceptives; however, since the DDD is very low for hormones, this will not correlate with the amount sold in kg [33].

When observing the pharmaceuticals consumption data on European countries (Table 2), namely, the amount consumed per year, we can realize that the amount used in Switzerland and Sweden is lower than the rest of the countries. This is explained by the fact that they have a significantly lower population when compared to the other countries referred to in Table 2 (Germany, France, Italy and Spain).

Besides the differences in population, different patterns are also observed between countries, even within each therapeutic group; however, some trends are clear regarding the global consumption of therapeutic groups. Anti-inflammatories are clearly the group with higher consumption (in kg), being PARA the pharmaceutical with the highest consumption. This group is followed by the antiepileptic CAR, with particularly high values in Germany. Antibiotics and lipid regulators have similar consumption patterns; nonetheless, these groups have great variations within them, showing distinct trends in different countries. Anxiolytics, SSRIs and hormones, in decreasing order, were the therapeutic groups with the lowest consumptions.

One should note that there are often discrepancies between pharmaceuticals sold and those actually consumed, due to delays between sales and actual use of medication. Moreover, patterns of local consumption might differ from those observed on a national scale [34,35].

### 2.3. Mechanism of Action, Metabolization and Excretion

Pharmaceuticals have different mechanisms of action resulting in several therapeutical indications, which differ between therapeutic groups. However, within each group, some variations can also occur, since there is more than one class of pharmaceuticals in each group.

The therapeutic group of anxiolytics include pharmaceuticals from the class of benzodiazepines like ALP and LOR, which are used for numerous indications, including anxiety, insomnia, muscle relaxation, relief from spasticity caused by central nervous system pathology and epilepsy. They act by binding to gamma-aminobutyric acid, increasing its activity, reducing the excitability of neurons and promoting a calming effect on the brain [36]. Although the hypnotic ZOL is not a benzodiazepine, it also acts on gamma-aminobutyric acid, promoting a shorter effect than benzodiazepines [37].

The selected antibiotics belong to two different classes, fluoroquinolones (CIP) and macrolides (AZI, CLA and ERY), which inhibit bacterial growth. Fluoroquinolones act by inhibiting bacterial DNA synthesis, and macrolides link to the bacterial ribosomes, inhibiting protein biosynthesis [38,39].

Lipid regulators drugs are used to treat dyslipidaemias; primarily, raised cholesterol. Statins like SIM have the capacity to reduce the endogenous cholesterol synthesis by inhibiting the principal enzyme involved. The fibrates (BEZ and GEM) increase the expression of some proteins in the liver, which results in a substantial decrease in plasma triglycerides and is usually associated with a moderate decrease in cholesterol concentrations [40,41].

The antiepileptic CAR has been extensively used in the treatment of epilepsy, as well as in the treatment of neuropathic pain and affective disorders, mainly due to the inhibition of sodium channel activity [42].

The SSRIs (CIT, ESC, FLU, PAR and SER) are antidepressants that, via inhibition of the serotonin reuptake mechanism, induce an increase in serotonin concentration within the central nervous system [43]. It should be noticed that CIT is a racemic mixture of *R*-citalopram and *S*-citalopram enantiomers with different potencies, but since *S*-citalopram is more potent, it is also marketed as the single *S*-enantiomer formulation ESC [44].

The anti-inflammatories DIC, IBU and NAP are non-steroids, and their mechanism of action is through inhibition of cyclooxygenase (1 and 2) in the periphery and central nervous system, reducing pain and inflammation but also other physiologic processes [45]. As for PARA, it acts on cyclooxygenase (2 and 3) in the central nervous system and only reduces pain and fever [46].

Finally, the hormones E1 and E2 are estrogen sex hormones, mainly female, and although they regulate the reproductive system, they also act in very different endocrine systems. As pharmaceuticals, E2 is mostly used in hormone replacement therapy, and EE2, a synthetic hormone more potent than E2, is primarily used in oral contraception [47,48].

According to other authors, pharmacokinetic data could provide a better knowledge of the environmental fate of pharmaceuticals, especially in the water compartment [30,49].

After consumption, pharmaceuticals are metabolized and primarily excreted in urine and feces as a mixture of the parent compound and its metabolites. The elimination in urine and/or feces is driven by two mechanisms, Phase I and Phase II metabolites. The first one uses the hepatic metabolism and, through biochemical oxidations, reductions and hydrolysis, increases the polarity and water solubility of the metabolites. Phase II metabolites are produced by a biochemical reaction through a conjugation step (i.e., glucuronidation and sulphation), where polar groups are transferred to parent compounds or metabolites, allowing these conjugated metabolites to become enough hydrophilic and water soluble to be eliminated through urine and/or feces [1,50,51]. These processes usually promote the loss of pharmaceutical activity of the compound. However, there are pharmaceuticals that are only active after metabolic activation by enzymatic system(s) of the parent compound (pro-drugs) to metabolite(s) [1].

To determine this pharmacokinetic feature, the proportion of the unchanged active molecule excreted in urine and/or in feces and the proportion of the parent molecule excreted as conjugates (glucuronide and sulphate) was included when available [52,53] (Table 3). The excretion rate, in addition to the consumption data, contributes to either a greater or lesser environmental impact and is related to the reported occurrence of the parent compound and its metabolites in the aquatic compartment [30,54]. Therefore, the excretion features were revised and are presented in Table 3.

While several publications are available on the metabolism of pharmaceuticals, the results of these studies can vary. The observed differences are probably explained by genomically distinct metabolizing capacities, as well as differences in race, sex, age and health status of the studied subjects, which are all known to affect the route and rate of metabolism [54,72]. SSRIs are clearly the therapeutic group with lower excretion rates, ranging from 0.2% to 23%, whereas the other groups present higher variability. The compounds with higher excretion rates are CIP (84%), PARA (80%), LOR (73%), BEZ (72%), E2 (68%) and GEM (50%).

## 3. Physicochemical Properties and Fate

### 3.1. Physicochemical Properties

The fate and persistence of the excreted pharmaceuticals and/or metabolites in the aquatic environment depend upon their physicochemical properties and the chemical and biological characteristics of the receiving water compartment. Several important chemical measurements of the pharmaceuticals in study, such as pKa (acid dissociation constant), log K_ow_ (octanol-water partitioning coefficient), log D_ow_ (the pH-dependent n-octanol-water distribution ratio), log Koc (soil organic carbon-water partitioning coefficient) and solubility, are presented in Table S1 (supporting information). These features can provide strong evidence of the ionization state of the compounds, their hydrophobicity and can help determining whether they will partition into water, biosolids, sediment and/or biological media [28,73].

Some authors defend that the log K_ow_ and log K_oc_ approaches are excessive restrictive models of pharmaceuticals distribution in the environment. In complex natural water and wastewater samples, partitioning due to hydrophobicity/lipophilicity is not the only physicochemical force of attraction operating between molecules. Electrostatic interactions, chemical bounding and nonspecific forces between ionized molecules and dissolved organic matter are neglected through exclusive log K_ow_ and K_oc_ approaches. Some studies have illustrated that water pH could play an important role in the interactions between organic matter and pH-depending pharmaceuticals, since there is a great variability between these compounds as regard to their pKa (4.0–18.3) [1]. Therefore, the log D_ow_ and log K_oc_ values presented in Table S1 (supporting information) are specific for pH 7.4, a value close to the ones usually observed in the water compartments (wastewater and surface water) [29,73,74].

With a log D_ow_ superior to 1, the likelihood of predominance of the chemical in the aqueous phase decreases logarithmically, whereas below a log D_ow_ of -1, the likelihood of predominance of the chemical in the aqueous phase increases logarithmically. Therefore, compounds having log D_ow_ values between -1 to +1 could be anticipated to be distributed in both the water and organic phases [73].

As seen in Table S1 (supporting information), the physicochemical properties of pharmaceuticals show a high variability. For example, the log D_ow_ ranges from -2.23 to 4.6, the log K_oc_ varies between 0 and 3.88 and even solubility goes from 0.1 to 101,200 (mg L^−1^). These variations are not only observed between different therapeutic groups but also within each group, since, as previously referred, this pharmaceuticals grouping does not correspond to similar chemical structures and there are more than one class per group. This can be seen especially for antibiotics, lipid regulators and anti-inflammatories, where greater fluctuations in these parameters are reported.

In summary, although pharmaceuticals present different physicochemical properties, some are expected to be more lipophilic and others to sorb to soils and sediments, they all have relatively high water solubility, having the potential to contaminate the aquatic environment [75].

### 3.2. Fate in Wastewater Treatment Plants

After excretion, pharmaceuticals are transported to WWTPs through the sewer system, and no significant removal occurs during transport in sewer pipes to WWTPs [76]. As hotspots of aquatic contamination, WWTPs play an important role in the life cycle of pharmaceuticals, since many are incompletely removed by conventional treatment processes and behave as persistent organic micropollutants [77].

The removal of pharmaceuticals in WWTPs is a complex phenomenon with many plausible mechanisms; additionally, these facilities are generally not equipped to deal with complex pharmaceuticals, as they were built and upgraded with the principal aim of removing easily or moderately biodegradable carbon, nitrogen and phosphorus compounds and microbiological organisms [18,78]. The main mechanisms involved in the removal of pharmaceuticals by WWTPs are filtration; biodegradation (e.g., oxidation, hydrolysis, demethylation and cleavage of glucuronide conjugates); sorption to sludge or particulate matter (by hydrophobic or electrostatic interactions) and chemical oxidation. Loss by volatilization can be considered as negligible [79,80,81].

WWTPs employ a primary, a secondary and an optional tertiary treatment process, being the last one is always associated with a high treatment cost. During primary treatment, physical removal of solids is achieved through a sieve, regularly followed by coagulation-flocculation processes for the removal of particulate matter, as well as colloids and some dissolved substances; however, this process is ineffective for the elimination of pharmaceuticals [82]. In the secondary treatment, usually with activated sludges, pharmaceuticals are subjected to a range of processes, including dispersion, dilution, partition, biodegradation and abiotic transformation, being biodegradation and sorption to solids are the main removal pathways of pharmaceuticals during this biological treatment. Afterwards, some WWTPs possess tertiary treatments like advanced oxidation processes, ultraviolet radiation (UV) or ozonation [82,83]. Most of the WWTPs in northern Europe comprise tertiary wastewater treatment; however, in other countries, they are less frequent [18].

Besides the type of wastewater treatment, WWTPs’ efficiency in removing pharmaceuticals is influenced by operational and environmental conditions, namely, the hydraulic retention time (HRT) (high HRT allows reactions like biodegradation and sorption mechanisms to occur); solid retention time (SRT) (which controls the size and diversity of the microbial community, and higher SRT will facilitate the build-up of slowly growing bacteria enhancing removal); environmental temperature (since higher temperatures reflect superior removal efficiencies) and pH conditions (affecting on the degradation kinetics of the compounds) [50,78,82,84,85].

As previously mentioned, the physicochemical characteristics of the pharmaceuticals also affect their removal in WWTPs. Since a significant part of the removal process is through sorption or biodegradation in sludge, the ability to interact with solid particles plays a major role. Thus, compounds with low sorption coefficients tend to remain in the aqueous phase, favoring their mobility through the WWTPs and into the receiving waters [86,87]. Independently of their physicochemical characteristics, some authors state that the portion of some pharmaceuticals in the treated sludge is negligible (<20%) when compared to the aqueous fraction for NAP, DIC, BEZ, GEM, LOR and CAR, although higher sorption removals were noted for selected compounds (AZI, CIP, IBU, PAR and PARA) [29,85].

Generally, during secondary treatment, compounds with log D_ow_ higher than 3, which indicates high sorption potential, tend to be removed through sorption onto sewage sludge, while compounds with log D_ow_ between 1.5 and 3 are removed mainly by biodegradation. The remaining pharmaceuticals with log D_ow_ inferior to 1.5 tend to remain dissolved [50,80,82,88]. Therefore, it is expected that the removal efficiency of substances with higher log D_ow_ are more influenced by SRT, while compounds with low log D_ow_ are more influenced by HRT [78]. During the secondary treatment, besides sorption to sludges, another removal mechanism is through microbial degradation, where nitrifiers are the most important group. This mechanism has been described as the main removal pathway for polar acidic pharmaceuticals; however, they are also sensitive to inhibitors, and some pharmaceuticals can have this effect on these microorganism [89,90].

Currently, besides the conventional treatments, new methodologies have been applied as tertiary treatments with higher removal efficiencies, but some of these new methods have high construction, maintenance and energy costs associated [77]. Advanced oxidation processes that include UV, ozone and hydrogen peroxide, among others, can also be used. UV treatment has been shown to partially remove some pharmaceuticals; however, it does not completely eliminate them [49,64,91,92]. Ozonation alone promotes the partial oxidation of pharmaceuticals, and to overcome this drawback, this process has been combined with heterogeneous catalysts or membrane technologies, such as nanoparticles of titanium dioxide, a known photocatalyst [11,77,82]. Adsorption by activated carbon is another methodology that proves to be effective in removing pharmaceuticals, with powdered activated carbon and granular activated carbon widely used in these adsorption processes. Generally, efficient removals are obtained when the compounds have nonpolar characteristics, as well as matching pore size/shape requirements. The main advantage of using activated carbon to remove pharmaceuticals is that it does not generate toxic or pharmacologically active products [82,93].

More recently, the growing trend of improving sustainability and reducing energy demands in WWTPs have encouraged alternative methods, such as algae ponds for secondary effluent polishing, with promising results [29].

As previously referred, metabolization in the human body can lead to elimination of pharmaceuticals conjugates. However, these phase II metabolites can be converted back into the parent compound, especially in WWTPs, being infrequently found in surface waters. One of the mechanisms used is the action of a β-glucuronidase enzyme produced by *Escherichia coli* capable of deconjugating the β-glucuronated pharmaceuticals excreted by the human body, resulting in the release of the active pharmaceutical into the wastewater [29,50,89,94,95]. On the other hand, the WWTPs processes responsible for pharmaceuticals elimination do not commonly lead to their complete mineralization; instead, breakdown products can emerge, which can also be toxic to the environment. In general, there is still a knowledge gap concerning the generation of metabolites and transformation products of known contaminants, which can potentially be as hazardous, or even more, than the parent compounds and can be present in different aquatic bodies at a higher concentration than parent compounds [90,96,97,98].

Naturally, the type of treatment can affect not only the removal efficiencies but also the metabolites and transformation products generated.

This supports the need for the evaluation of metabolites and transformation products and the further development of new treatment techniques to achieve complete mineralization of emerging contaminants [90,97]. Besides the fact that some of the new treatments, like advanced oxidation processes, can originate toxic transformation products, they have higher efficiencies when compared to traditional treatments [77,82,99,100].

**Figure 1:**
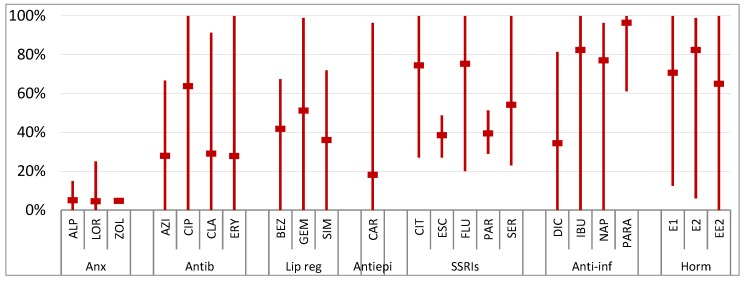
Minimum, maximum and average removal efficiencies in WWTPs (%). Anx—anxiolytics, Antib—antibiotics, Lip reg—lipid regulators, Antiepi—antiepileptics, SSRIs—selective serotonin reuptake inhibitors, Anti-inf—anti-inflammatories and Horm—hormones [3,5,13,16,18,51,59,63,67,68,71,78,79,80,81,82,85,87,88,92,99,102,103,104,105,106,107,108,109,110,111,112,113,114,115,116,117,118,119,120,121,122,123,124,125,126,127,128,129,130,131,132].

Data from 52 publications were collected, and removal efficiencies of the selected pharmaceuticals are summarized in Figure 1. One should note that, although we are comparing the fate of pharmaceuticals in WWTPs, there are some countries with inadequate wastewater and collection infrastructures or even functional WWTPs. For example, in Ghana and India, only 7.9% and 30.7% of the wastewaters are treated, which anticipates that the presence of pharmaceuticals in the aquatic environment in these countries should represent an even bigger problem [101].

Although, as mentioned, some studies indicate that physicochemical properties set the efficiency of removal of pharmaceuticals in WWTPs, the literature review performed showed that the target compounds present very different removal rates, ranging between negative and high removal rates, and no obvious pattern in behavior was observed, even within the same therapeutic group, implying that factors other than compound-specific properties affect removal efficiency [68,85]. Negative values for some compounds have been reported and may reflect deconjugation of metabolites during the treatment process or changes in the adsorption to particles during treatment [133]. Generally, what becomes evident is that the elimination of most pharmaceuticals is incomplete, and it is not exclusively related neither to the physicochemical properties nor to the type of treatment processes. Additionally, most pharmaceuticals have always one report that shows no removal [16,18,85,88].

Concerning the removal efficiencies of each therapeutic group, anxiolytics present the lowest average, having a small variation due to their similar physicochemical properties, with values ranging from 0% to 25%. Although their log D_ow_ (from 2.49 to 3.06), higher than most of the selected pharmaceuticals, predicted large sorption to sludge and higher removal rates, this was not observed in real removal data.

As for antibiotics, the range observed in the removal efficiencies was from 0% to 100%, similar to anti-inflammatories and hormones. The average removal rates for AZI, CLA and ERY (macrolides) are near 30%, whereas CIP presented higher removal rates (64%). Despite the lower log D_ow_ for CIP (-2.23) sorption to sludges, it has been suggested as the primary removal mechanism for fluoroquinolones, whereas, for macrolides, limited sorption to sludge is observed [108,132,134].

Although the therapeutic group of lipid regulators encloses a statin (SIM) and fibrates (BEZ and GEM) and their removals vary between 0% and 99%, their averages are similar, ranging from 36% to 51%, being also found in sludges [33].

For CAR, although presenting a lower log D_ow_ (2.28) than anxiolytics and a wide range of removal efficiencies, it is one of the most persistent compounds and is averagely reduced by only 18.1% [135,136]. This pharmaceutical is very resistant to wastewater treatments, since it has low biological degradation and sorption and has only higher removal rates with the use of advanced treatments such as ozonation together with the usage of the photocatalyst titanium dioxide [134,135].

Regarding SSRIs, even though they all belong to the same group, the average removal efficiencies range from 39% to 75%, with ESC, PAR and SER presenting lower values, below 55%, when compared to CIT and FLU that present higher removal rates, 75%.

The most investigated therapeutic group in WWTPs are anti-inflammatories, and despite their high variability, average removal rates are above 77% and up to 96% (PARA), with the exception for DIC (34%) [82,135]. Excluding DIC, anti-inflammatories undergo sorption to sludges and biological and photolytic degradation [33,82,89,96,137]. As for DIC, sorption to sludge and biodegradability have been reported but to a lower extent, translating into low elimination rates during wastewater treatment; moreover, a low removal efficiency of 4-OH-DIC has been reported in WWTPs [89]. Advanced oxidation processes are described as highly efficient for DIC removal, since it rapidly decomposes by direct photo-oxidation, indicating that this pathway is one of its main degradation mechanisms. However, ozonation alone is not completely effective, but the O_3_/H_2_O_2_ system shows high efficacy [11,135]. On the other hand, PARA, which has the higher removal rate during wastewater treatment, can generate different transformation products, being 4-PARA was identified as the main one, and its presence in wastewater samples was already reported. However, there are other possible sources, since it is also widely used in industrial applications and is a known transformation product from pesticides. Furthermore, 4-PARA was also described as the primary degradation product of PARA during storage [138].

Hormones are the therapeutic group with higher log Dow and high average removal efficiency, which ranges from 65% to 82%. This low variation was expected, since the molecules have similar physicochemical properties [82]. Although most hormone conjugates are degraded in the WWTPs, some are still observed in WWEs representing less than 33% of the parent compound (E1 and E2), which can be reconverted back into the parent compound in the environment [50,139]. It is also possible that E2 can be converted in E1 in the WWTPs, possibly explaining the higher removal rate for this pharmaceutical [71]. Once again, advanced oxidation processes are described as highly efficient processes in hormone removal [11].

As observed, the WWTPs are unable to completely remove the pharmaceuticals, and through direct discharge of WWEs in surface water or by land application of WWTPs’ sludge or through leaching, these facilities are the major sources of pharmaceuticals in the environment [29,59,79,140,141].

Optimization of wastewater treatment still remains a task of high priority. Biological treatment is commonly unable to remove pharmaceuticals; however, its efficacy can be improved under favorable conditions. Although advanced treatment technologies, such as membrane and advanced oxidation processes, have been promising for pharmaceuticals removal, high operation costs and formation of degradation products still remain an issue [82].

### 3.3. Fate in Surface Waters

Since WWTPs are not able to completely remove pharmaceuticals, they are disseminated through their WWEs and sludges, mostly, into surface waters. In the aquatic environment, the fate and concentration of pharmaceuticals can be reliant on the receiving water body flow rate, partitioning to sediments, biological entities and consequent degradation, uptake by biota, volatilization, photodegradation or transformation through other abiotic mechanisms, such as hydrolysis [29,74,134,142].

When WWEs reach the surface waters, the dilution effect varies significantly due to different flows in different rivers; however, this effect can be relatively low, especially in arid or semi-arid regions due to water scarcity, like some Iberian rivers, where other processes gain relative importance [143,144]. Although multiple biotic and abiotic routes could transform pharmaceuticals once they reach the surface water, the predominant pathways to remove pharmaceuticals are photodegradation and sorption [77,143].

The fate of different pharmaceuticals has already been studied in surface waters by several authors using estimates of mass loading, dilution and in-stream attenuation, here understood as the reduction of the concentration of pharmaceuticals along the river segment by processes different from dilution [28,74,98,141,143].

Overall, it is expected that the log D_ow_ of a given compound influences its in-stream attenuation; in the case of hydrophobic compounds (with higher log D_ow_), sorption to suspended particles and sediments is a dominant process leading to in-stream attenuation by reducing the concentration in the aqueous phase along the river segment [74]. In this way, these compounds become less exposed to other biotic (biotransformation) and abiotic (photolysis and volatilization) transformation processes and, therefore, become less affected by the variation of environmental conditions between river segments. Therefore, it is expected that compounds with low log D_ow_ show not only more differences in attenuation rates between sites but also more temporal differences (i.e., seasonal and day–night) within each site [143]. This sorption mechanism in the aquatic environment represents an important sink for pharmaceuticals, as it has been suggested that strong pharmaceutical interactions may act as a long-term storage of pharmaceuticals that will increase their persistence, while their bioavailability in the environment is reduced, being recalcitrant to microbial degradation [28,33]. In fact, the sediments could be a source of contaminants in downstream river segments if resuspension of fine-grained bedded sediments occurs, for instance, during seasonal increases in flow rate or during flood events [143]. Moreover, the activity of benthic invertebrate in sediments can result in an increased desorption, leading to improved bioavailability in the water compartment [29]. Additionally, sorption to colloids can also provide an important sink for the pharmaceuticals in the aquatic environment, increasing their persistence while reducing their bioavailability. In general, sorption may result in a biased risk estimation [9].

As already referred, in complex natural waters, electrostatic interactions, chemical bounding and nonspecific forces between ionized molecules and dissolved organic matter can also occur, meaning that we cannot generalize the attenuation of a compound based on its physicochemical properties alone [98,143]. However, the different log D_ow_ of pharmaceuticals influence the variability of rates among rivers, likely due to its effect on sorption to sediments and suspended particles, and therefore, influence the balance between the different attenuation mechanisms (biotransformation, photolysis and sorption) [143].

The attenuation of pharmaceuticals was evaluated in surface water in Spain where the total concentration of pharmaceuticals (CLA, DIC, IBU, BEZ, GEM, CAR and CIT) decreased about 40% in less than 5 km, although the number of compounds detected only decreased 13% [74]. Studies also reported that GEM is a quite persistent compound in surface water, with half-lives ranging from 70 to 288 days [137]. As for CIP, photodegradation is reported to be the main mechanism of attenuation [90]. However, for CAR, there are reports evaluated in a Swedish lake where no attenuation was observed and with an estimated half-life of 780-5700 days [98]. This was also supported by other studies that revealed that CAR and IBU were stable against sunlight, while PARA suffers moderate photodegradation and DIC was rapidly photodegraded in surface water [90,145]. Accordingly, another study noticed that no biodegradation of IBU was observed in a sterile river, but in river water and using microbial biofilms, biodegradation occurred in a few hours, evidencing that although its transformation is a complex process, microorganisms play an important role in IBU degradation [137]. Concerning SSRIs, which have high sorption coefficients, they have proven to be persistent compounds, and FLU demonstrated that it was far more resistant to photolysis than the other SSRIs, with a half-life of 122 days [28].

Besides the presence of the parent compounds in surface waters, sulphate conjugates of E1 and E2 have already been observed. Although these conjugates no longer possess a significant biological activity, they can act as precursor steroid reservoirs that might be converted into free estrogens [128,139]. Even though the synthetic hormone EE2 has lower solubility than E2, it is also considerably more persistent in the aquatic environment, with an estimated half-life in surface water between 1.5 and 17 days [146].

In addition to the parent compounds, some studies also addressed the contribution of WWTPs for pharmaceuticals transformation products in surface waters and confirmed that these facilities were a major source of contamination to the recipients [74,98].

In summary, on one hand, the emissions from WWEs vary widely because of differences in regional usage of the compounds and efficiency of WWTPs. On the other hand, the processes that drive in-stream attenuation (i.e., biotransformation, photolysis, sorption and volatilization) depend on the different pharmaceutical characteristics, as well as on a series of physicochemical and biological parameters of the river, such as river flow rate, temperature, the vertical hydrological exchange between surface and subsurface compartments, turbidity, dissolved oxygen concentration, biofilm biomass and pH [143]. The magnitude of the measured attenuation rates urges scientists to consider them as important as dilution when aiming to predict concentrations in freshwater ecosystems. Since pharmaceuticals are continuously introduced in surface waters and are not completely removed, they eventually will reach groundwater, seawater, mineral water and drinking water, contaminating all aquatic compartments [98].

## 4. Occurrence

Along with advances in analytical instruments and techniques, trace levels of various pharmaceuticals and their metabolites have been detected in the aquatic compartment since the latter half of the 1970s [145].

**Figure 2:**
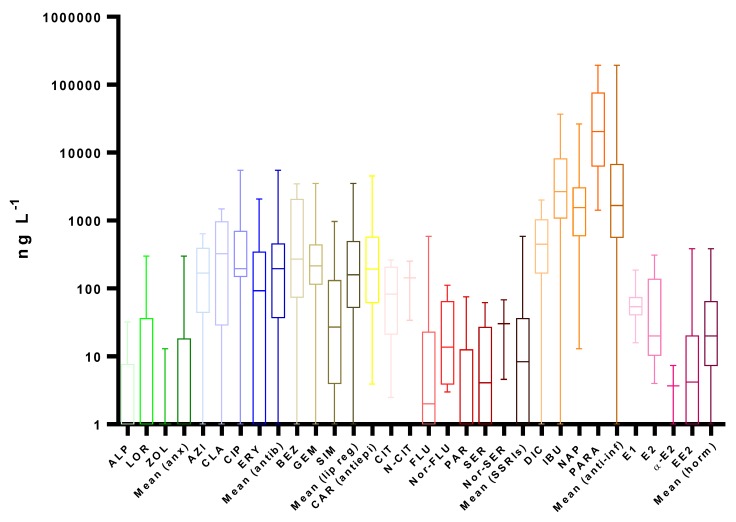
Boxplots with median, maximum and minimum average concentrations of pharmaceuticals in wastewater influents (WWIs). Anx—anxiolytics, Antib—antibiotics, Lip reg—lipid regulators, Antiepi—antiepileptics, SRRIs—selective serotonin uptake inhibitors, Anti-inf—anti-inflammatories and Horm—hormones [3,13,15,16,18,29,34,59,63,67,68,71,78,79,82,83,86,87,94,96,100,102,107,108,109,111,112,113,114,115,117,118,119,120,122,123,126,128,130,131,132,133,140,147,148,149,150,151,152,153,154,155,156,157,158,159,160,161,162,163,164,165,166,167,168,169,170].

**Figure 3:**
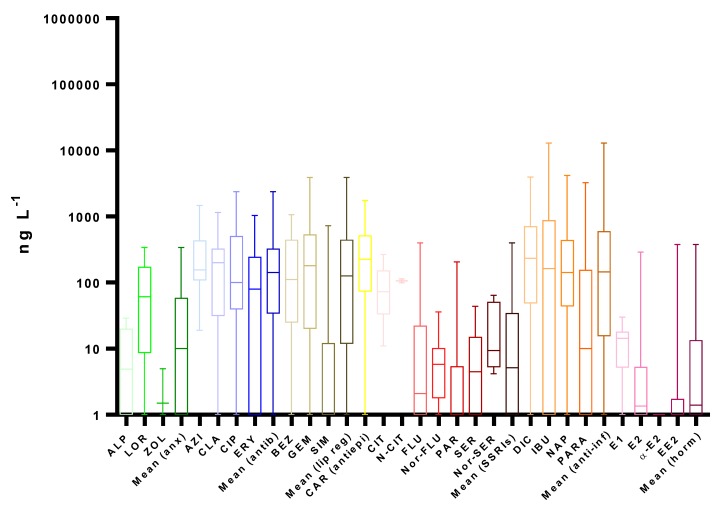
Boxplots with median, maximum and minimum average concentrations of pharmaceuticals in wastewater effluents (WWEs). Anx—anxiolytics, Antib—antibiotics, Lip reg—lipid regulators, Antiepi—antiepileptics, SSRIs—selective serotonin reuptake inhibitors, Anti-inf—anti-inflammatories, Horm—hormones and different letters represent significant statistical differences) [3,5,6,8,13,15,16,18,29,34,59,63,67,68,71,78,79,82,83,86,87,91,94,96,100,102,103,107,108,109,111,112,113,114,115,117,118,119,120,122,126,128,129,130,131,132,133,136,139,140,141,146,147,149,150,152,153,154,155,156,157,158,159,160,161,162,163,164,165,166,167,168,169,170,171,172,173,174,175,176,177,178,179,180,181,182,183].

**Figure 4:**
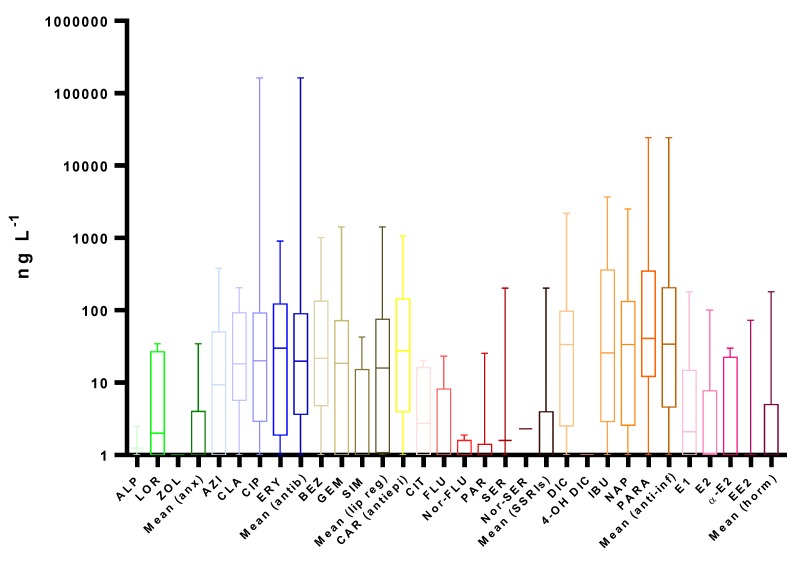
Boxplots with median, maximum and minimum average concentrations of pharmaceuticals in surface waters. Anx—anxiolytics, Antib—antibiotics, Lip reg—lipid regulators, Antiepi—antiepileptics, SSRIs—selective serotonin reuptake inhibitors, Anti-inf—anti-inflammatories, Horm—hormones and different letters represent significant statistical differences [1,5,6,29,34,58,59,73,74,82,96,98,101,113,119,122,128,129,132,134,136,138,139,140,141,145,146,147,154,155,157,158,159,161,163,164,166,167,169,170,171,174,175,176,178,179,180,181,182,184,185,186,189,190,191,192,193,194,195,196,197,198,199,200,201,202,203,204,205,206,207,208,209,210,211,212,213,214,215,216,217,218,219,220,221,222,223,224].

**Figure 5:**
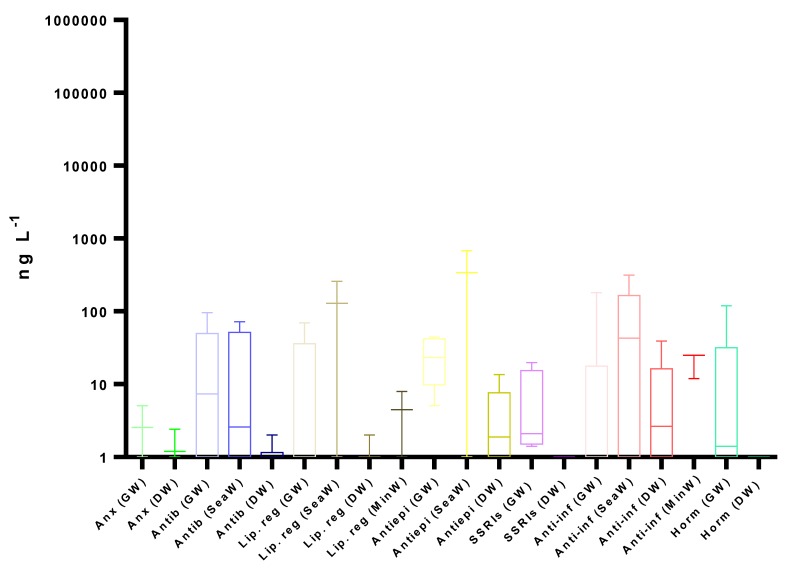
Boxplots with median, maximum and minimum average concentrations of pharmaceuticals in other water bodies. GW—groundwaters, SeaW—seawaters, DW—drinking waters, MinW—mineral waters, Anx—anxiolytics, Antib—antibiotics, Lip reg—lipid regulators, Antiepi—antiepileptics, SSRIs selective serotonin reuptake inhibitors, Anti-inf—anti-inflammatories, Horm—hormones and different letters represent significant statistical differences [1,6,82,91,113,128,136,141,145,166,167,174,179,189,193,200,206,209,213,215,217,225,226,227,228,229,230,231].

A literature review on worldwide monitoring programs in recent years, presented in Figure 2, Figure 3, Figure 4 and Figure 5 and Tables S2–S5 (supporting information), clearly reveals the ubiquitous distribution of pharmaceuticals in different aquatic environment compartments, including WWIs, WWEs and surface waters, with concentrations up to mg L^−1^ [145,147]. Usually, this occurrence is related to the gross domestic product per capita of each country and is presented as the shape of an inverted-U; i.e., pollution worsens as the economy of countries starts to grow (increased consumption of pharmaceuticals), and then it improves when countries reach a higher stage of economic growth (improved WWTPs) [101].

Beside the aspects previously referred, several others can influence the concentration of pharmaceuticals in the different aquatic compartments, promoting a great variability in the detected concentrations. In WWTPs, other aspects that can influence the detected concentrations are the flow rate, the time of the year, the temperature, the type of WWTPs, day and the type of sampling, etc. [105]. As for surface waters, the flow rate, temperature, sunlight, time of the year, day and the type of sampling are also parameters that can influence pharmaceuticals concentrations [23]. Moreover, some of these parameters can also influence the detected concentrations in other water bodies.

### 4.1. Wastewater

#### 4.1.1. Wastewater Influents

Figure 2 and Table S2 (supporting information) summarizes the median, averages and maximum concentrations of the targeted pharmaceuticals in the WWIs across the world, collected from 66 references. These concentrations are likely to be influenced by both consumption data and excretion rates.

All investigated pharmaceuticals were frequently detected in WWIs, with PARA, CIT, IBU, CAR, BEZ, CLA and α-E2 (E2 isomer) presenting detection frequencies higher than 88%. As for the different therapeutic groups, antiepileptics and anti-inflammatories were the ones with higher detection frequencies, above 86%, followed by lipid regulators (75%) and hormones (74%). Anxiolytics were the group with lower values (31%), much different from the other groups. The highest median concentration (1.7 µg L^−1^) was observed in the anti-inflammatories group, with statistical differences for all of the other therapeutic groups, being the maximum individual concentration observed for IBU (700 µg L^−1^) [78]. Antibiotics, lipid regulators and the antiepileptics had median concentrations between 160 and 196 ng L^−1^, followed by the other groups, with medians under 20 ng L^−1^.

Although anxiolytics were the group with the lower detection frequency and median, ALP had concentrations up to 4.7 µg L^−1^. Additionally, the highest detection frequency belonged to LOR, with 38% [150]. These results are in line with data already mentioned, such as the low consumption and low excretion rates observed for this therapeutic group. The anxiolytic with the highest excretion rates and consumption is LOR, which is reflected on the occurrence reported.

Antibiotics were the most homogenous group, with median concentrations ranging from 93 to 324 ng L^−1^ and with all detection frequencies above 65%. Although some discrepancies in excretion rates, with higher values for CIP, both CIP and CLA have higher consumptions, being this pattern was observed in the occurrence data.

Lipid regulators occurrence data was comparable to that of antibiotics, mostly because of similar consumption and excretion rates. Within this group, we can observe that the one with the highest consumption in most countries, SIM, had the lowest detection frequency and median concentration in WWIs. This can be due to a significant difference in excretion data, where BEZ have clearly higher rates than SIM, with excretion values up to 72% and 12.5%, respectively [1,60]. Therefore, it is shown that a pharmaceutical with low consumption can reach relatively high detection frequencies and median concentration in WWIs (89% and 271 ng L^−1^, respectively).

The antiepileptic CAR with excretion rates up to 33%, and whose consumption is only surpassed by anti-inflammatories, had a detection frequency of 89% and concentrations up to 22 µg L^−1^ [25,111].

Like anxiolytics, SSRIs also had low consumption and excretion rates, which reflected also in low concentrations in the WWIs, with a median concentration of 8 ng L^−1^. However, this group presented some peculiarities, SER being one of them. This SSRI has the highest consumption in European countries. Nonetheless, due to its very low excretion rate (0.2%), this compound and its metabolite (Nor-SER) present lower median concentrations than CIT and Nor-FLU [56]. On the other hand, despite the low consumption data for CIT, its higher excretion rate explains the fact that this SSRI and its metabolite (N-CIT) are the ones with the highest concentrations within this therapeutic group, followed by FLU and its metabolite (Nor-FLU), that also present higher excretion rates (up to 11%) [65].

As referred, anti-inflammatories were the group with higher concentrations in WWIs, not only due to their high consumption but also to significant excretion rates (up to 80%), with median concentrations of 450, 1550, 2680 and 20 601 ng L^−1^ for DIC, NAP, IBU and PARA, respectively [69].

In the hormones group, although there were lower excretion rates observed for E2, its higher consumption (2.5 kg y^−1^) when compared to EE2 (0.7 kg y^−1^) resulted in higher concentrations even for its metabolite E1, being even present in the enantiomer of E2 (α-E2) up to 10 µg L^−1^ [155]. As previously mentioned, one should also take into account that both E1 and E2 are produced in the human body and can be excreted naturally [71,128].

These data highlight that pharmaceutical compounds with low excretion rates are not necessarily present at low levels in WWIs, because this could be offset by the massive use of these compounds [82]. Additionally, it was also observed that, in general, the mean pharmaceutical concentrations could vary between 1 to 3 orders of magnitude from one sampling day or week to the next. Diurnal trends were also observed, and peak concentrations were highly unpredictable [150].

#### 4.1.2. Wastewater Effluents

The first report of human pharmaceuticals in WWEs is from 1976, and subsequent studies have confirmed the presence of pharmaceuticals in this aquatic compartment [170]. After passing through WWTPs and being submitted to the different treatments already discussed, it would be expected that WWEs presented lower concentrations than the influent, with a decrease proportional to the removal efficiency of the WWTPs [18].

Data regarding 87 references were collected and summarized in Figure 3 and Table S3 (supporting information). In the effluents, the median concentrations of the therapeutic groups varied from 1.4 ng L^−1^, for hormones, to 226 ng L^−1^, for antiepileptics, and, in general, significantly lower concentrations were found when comparing to influent samples, as shown in Figure 2. However, since concentrations in WWIs, as well as removal efficiencies, have a wide variability, the range of concentrations in WWEs is still high [78].

In general, regarding the median concentrations, antiepileptics were followed by anti-inflammatories (146 ng L^−1^), antibiotics (142 ng L^−1^) and lipid regulators (126 ng L^−1^), a similar pattern to that in WWIs but with no statistical significance between them. The remaining three groups had lower medians, with 10, 5.2 and 1.4 ng L^−1^ for anxiolytics, SSRIs and hormones, respectively. The highest individual mean concentration observed was for DIC 233 ng L^−1^; however, the maximum concentration regarded CIP, 14 mg L^−1^. This high value, along with others that are completely offset, were observed in the effluents of pharmaceutical industries and hospitals [25,26,111,183].

Anxiolytics were the only therapeutic group with a clear higher median and individual concentrations in WWEs than in WWIs and surpassed the mean concentration of hormones and SSRIs. This is justified by the fact that anxiolytics have the lowest removal efficiencies, and, in some cases, even negative values are found. This increased concentration in WWEs is related to the transformation of metabolites and/or transformation products back into the parent compounds during wastewater treatment [80,82]. Since all the three compounds have similar removal efficiencies, LOR, with the highest concentration in WWIs, presented again the highest values in WWEs, both median (61 ng L^−1^) and individual (438 ng L^−1^) levels [94].

As indicated in Table S3 (supporting information), CLA was once again the antibiotic more frequently detected in WWEs (87%), and this group remained the most homogenic, with median concentrations ranging from 80 to 200 ng L^−1^. The extremely high value found for CIP was observed in the effluent of a pharmaceutical industry [111].

As regard to the antiepileptic CAR, the fact that it does not adsorb to soils and has low removal efficiencies in WWTPs results in a small increased median from WWIs to WWEs, from 193 to 226 ng L^−1^, respectively [184].

Lipid regulators having removal efficiencies analogous to those observed for antibiotics present an occurrence pattern in WWEs comparable to that of WWIs, again with SIM presenting the lowest median concentration (1 ng L^−1^).

The therapeutic group SSRIs had also the same pattern observed in WWIs, with CIT and N-CIT presenting the higher median concentrations of 73 and 107 ng L^−1^, respectively, and, once again, the metabolites (N-CIT, Nor-FLU and Nor-SER) concentrations were in the same range or higher as the parent compounds [118]. The highest value regarded CIT with 430 µg L^−1^, which was also detected in a pharmaceutical industry effluent [111].

Anti-inflammatories had one of the highest removal efficiencies, only comparable to hormones, and although they remain with a high median concentration, the difference to the other therapeutic groups (antiepileptics, lipid regulators and antibiotics) was significantly reduced. Within this therapeutic group, DIC presented the highest median concentration, followed by IBU, NAP and PARA, with 163, 142 and 10 ng L^−1^, respectively, meaning that PARA shifted from the highest median concentration in WWIs to the fourth in WWEs, mainly due to the high removal average (96%) presented.

As for hormones, with average removal efficiencies above 60%, concentrations were also significantly reduced, with the highest median concentration belonging to E1 (14 ng L^−1^) and the lowest to α-E2 (0.4 ng L^−1^); the highest individual value was also for α-E2 (4.7 µg L^−1^), observed in only one study [155].

Despite these concentrations, it is possible that some conjugates, which were not evaluated, enter surface waters, where they can be reconverted back to the parent compound, increasing the pharmaceuticals contamination burden [29].

As expected, some positive correlation could be observed between the concentrations found in WWIs and in WWEs with removal efficiencies. Nonetheless, even at relatively low population densities and low industrial and hospital activity, human pharmaceuticals are present at quantifiable levels in WWEs [170].

### 4.2. Surface Water

The release of WWEs into surface water, in comparison to other sources, has been considered the main cause of the presence of pharmaceuticals in this water body [59,184].

As previously discussed, following the treatment processes in WWTPs, pharmaceuticals are subjected to different degrees of natural attenuation. These conditions can promote a variation higher than one order of magnitude in the same sampling location and even higher between different rivers [19]. Due to these factors, pharmaceutical compounds are expected to occur in surface waters at lower levels than in WWEs [82,98,185].

Since 1970, the issue regarding the presence of chemicals in surface waters has been addressed by the EU. Nowadays, the chemical quality of surface waters is controlled under the WFD (Directive 2000/60/EC of the European Parliament and of the council of 23 October 2000, establishing a framework for community action in the field of water policy), transposed into the Portuguese legal system by the Law N 58/2005 of 29 December 2005 (the Water law). Within this framework, the key strategy adopted was the establishment of priority substances or groups of substances due to their persistence, toxicity, bioaccumulation, widespread use and detection in rivers, lakes, transitional and coastal waters. Additionally, a list of environmental quality standards have been issued for these substances, to ensure adequate protection of the aquatic environment and human health [8]. Although no pharmaceutical belongs to this list, their environmental presence in surface waters is a growing problem that must be tackled and was addressed by the WFD in order to minimize their aquatic environmental contamination and support future prioritization measures. Despite this awareness, legal limits have not yet been set for pharmaceuticals in surface water, although a watch list that includes seven pharmaceuticals (E2, EE2, AZI, CLA, ERY, amoxicilin and CIP) and one metabolite (E1) has been recently established [17,34,186,187]. IBU has also been proposed to enter this list; however, its inclusion was rejected in January 2012 owing to a lack of sufficient evidence of significant risks to aquatic environments [9].

According to the Directive 2013/39/EU strategy, all member states shall monitor each substance in the watch list at selected surface waters representative monitoring stations at least once per year. The number of monitoring stations varies within each member state, taking into account the population and area of each country. About 40% of European water bodies still have an unknown chemical status, as not even the monitoring of the EU priority substances have been performed [21].

After reviewing 88 scientific references, as expected, lower median concentrations (ten times lower) were found in surface waters than in WWEs (Figure 4 and Table S4 (supporting information).

We can observe similar patterns in WWEs, with the same four therapeutic groups presenting higher median concentrations, anti-inflammatories (34 ng L^−1^), antiepileptics (28 ng L^−1^), antibiotics (20 ng L^−1^) and lipid regulators (16 ng L^−1^). These four therapeutic groups had statistically significant higher median concentrations than SSRIs and hormones. SSRIs, hormones and anxiolytics, with notably lower values, had the lowest median concentrations of 0.8, 0.4 and 0 ng L^−1^, respectively. The highest values observed were reported for CIP in India, with a maximum concentration of 650 µg L^−1^ for CIP [6].

Regarding anxiolytics, only LOR and ALP were found in surface waters, with a detection frequency of 30%. ZOL was evaluated in only one study, which did not detect it [188].

As above mentioned, antibiotics were one of the therapeutic groups with high median concentrations (20 ng L^−1^). It also presented two extremely high average concentrations detected for CIP in surface waters near pharmaceutical industries in Pakistan (1.3 µg L^−1^) and in India (164 µg L^−1^); however, all the other average concentrations were below 108 ng L^−1^ [6,189]. Comparing the antibiotics concentrations with WWEs, a very similar pattern was observed, with a tendency for a relative higher detection frequency and concentration for ERY, probably revealing a higher persistency in the environment.

Lipid regulators presented similar patterns than in WWE, with SIM being the one with the lower median concentration. BEZ, apparently, presented higher persistence, since its detection frequency and median concentration, 67% and 22 ng L^−1^, respectively, surpassed those of GEM, 51% and 19 ng L^−1^, respectively.

As previously noted, CAR continued among the most frequently detected pharmaceutical compounds in surface waters (78%) and presented concentrations up to 12 µg L^−1^, reflecting, as expected, the recalcitrant nature of this molecule, given its high half-life [190]. In fact, it is also one of the most frequently detected pharmaceuticals in European surface waters [137].

The SSRIs values decreased from WWEs to surface waters in median concentrations (from 5.2 to 0.8 ng L^−1^) and in detection frequencies from 55% to 26%. The highest concentration regarded CIT (76 µg L^−1^); however, it was found, once again, near a pharmaceutical industry in India [6]. The metabolites suffer even a higher reduction than the parent compounds.

Anti-inflammatories presented, once again, higher concentrations when comparing with other therapeutic groups [170]. PARA presented the higher median concentration (41 ng L^−1^), followed by DIC and NAP (34 ng L^−1^) and IBU (26 ng L^−1^). The results of PARA, higher than the ones in WWE, move PARA to values in the same range as the other anti-inflammatories. Looking at the detection frequencies, they all fall in the same range, from 52% to 59%. In this group, another extremely high concentration was observed for PARA in Kenya 107 µg L^−1^ [166]. Although, in wastewaters, no study on 4-OH-DIC was reviewed, in surface waters, two studies were found and 40 ng L^−1^ was the highest concentration found for this metabolite [191]. The average concentration observed for DIC (221 ng L^−1^) was twice the purposed value of 100 ng L^−1^ for the environmental quality standard in 2012-2013. The high values in surface waters possibly raised some issues regarding the establishment of this standard.

Within the hormones group, E1 presented the higher median concentration (2.1 ng L^−1^), and the highest average value was detected in China, 180 ng L^−1^, whereas its detection frequency was slightly decreased (from 57% to 54%) [192]. Contrary to what was previously mentioned, namely that EE2 was more persistent than E2, EE2 registered a higher decrease in detection frequency (from 25% to 2%) than E2 (from 43% to 22%). In surface waters, conjugates of both E1 and E2 were also found in a concentration range from a quarter to half of the parent compound [139,193].

As above mentioned, lower concentrations of pharmaceuticals (ten times lower) were found in surface waters than in WWEs. Surface waters showed an overall trend of higher concentrations in sites influenced by the location of WWTPs [104,194].

### 4.3. Other Water Bodies

As discussed earlier, the concentrations of pharmaceuticals decrease from the WWIs to WWEs and to surface waters through different mechanisms. However, data collected from 28 references showed that pharmaceuticals can reach groundwaters, seawaters and even mineral waters and drinking waters (Figure 5 and Table S5 (supporting information)). Regarding groundwaters, it is important to underline that this is an important resource of water supply in the world, and it is especially vulnerable to contamination, although soil provides a big inertia to propagation of the contamination, and for that same reason, once contaminated, the effects can hardly ever be reverted [225].

The concentrations in remaining waters bodies should be lower than the previous ones, since they suffer attenuation mechanisms similar to surface water. Additionally, drinking water has dedicated treatment plants. However, these facilities do not completely remove pharmaceuticals and can also produce transformation products that can be toxic [145,173,199].

Although susceptible to degradation or transformation, pharmaceuticals’ continuous introduction into the aquatic environment confers some degree of pseudo-persistence, reaching, at extremely low concentrations, all aquatic compartments all over the world, even drinking waters [64,91]. However, it is unlikely that pharmaceuticals pose significant threats to human health at the concentrations that may occur in drinking waters [145,231].

In Figure 5, we observe that, once again, antibiotics, lipid regulators, antiepileptics and anti-inflammatories had higher detection frequencies and median concentrations; however, CAR stands out from the others with a higher detection frequency and average concentration of 45% and 60 ng L^−1^, respectively. Groundwater and seawater were the water bodies with higher detection frequencies and concentrations, and the highest concentration found was of 14 µg L^−1^ for CIP in groundwater [6]. No statistical significance was observed between the different therapeutic group averages.

## 5. Final Remarks

A careful literature review was conducted in order to understand the sources, fate and occurrence of pharmaceuticals in the aquatic environment. In this context, a broad and specialized background was obtained, enabling a complete overview of the state-of-the-art in these subjects.

The data provided in this review evidenced that WWTPs are the major source of pharmaceuticals contamination. It is also noteworthy that pharmaceuticals belonging to the same therapeutic group can have distinct physicochemical properties, resulting in different behaviours both in WWTPs and in the aquatic environment.

The concentrations of pharmaceuticals found in the aquatic bodies were, in decreasing order, WWIs, WWEs, surface water and other water bodies.

Overall, these results present a global picture of the pharmaceuticals’ contamination, an important input for setting prioritizing measures and sustainable strategies to minimize their impact in the aquatic environment.

## Figures and Tables

**Table 1:** Selected pharmaceuticals.

Therapeutic Group	Compound and Chemical Structure			
**Anxiolytics and Hypnotics (Anx)**	Alprazolam (ALP)	Lorazepam (LOR)	Zolpidem (ZOL)	
	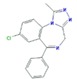	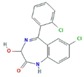	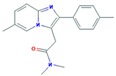	
**Antibiotics (Antib)**	Azithromycin (AZI)	Ciprofloxacin (CIP)	Clarithromycin (CLA)	Erythromycin (ERY)
	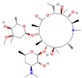	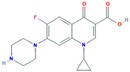	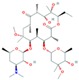	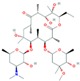
**Lipid Regulators** **(Lip Reg)**	Bezafibrate (BEZ)	Gemfibrozil(GEM)	Simvastatin (SIM)	
	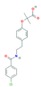	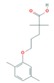	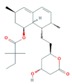	
**Antiepileptic (Antiepi)**	Carbamazepine (CAR)			
	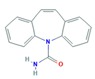			
**Selective Serotonin Reuptake Inhibitors (SSRIs)**	Citalopram (CIT)	Desmethylcitalopram (N-Cit) (metabolite)	Escitalopram (ESC)	Fluoxetine (FLU)
	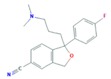	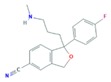	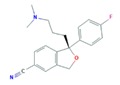	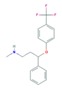
	Norfluoxetine (Nor-FLU) (metabolite)	Paroxetine (PAR)	Sertraline (SER)	Desmethylsertraline (Nor-SER) (metabolite)
	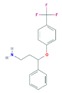	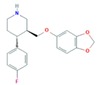	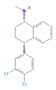	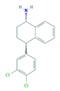
**Anti-Inflammatories (Anti-inf)**	Diclofenac (DIC)	4-hydroxydiclofenac (4-OH-DIC) (metabolite)	Ibuprofen(IBU)	Naproxen (NAP)
	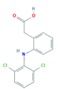	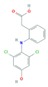	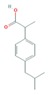	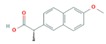
	Paracetamol (PARA)	4-aminophenol (4-PARA) (transformation product)		
	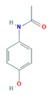	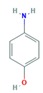		
**Hormones (Horm)**	Estrone (E1) (natural hormone/metabolite)	17β-estradiol (E2)	17α-ethinylestradiol (EE2)	
	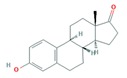	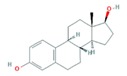	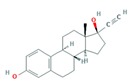	

**Table 2:** International consumption of the selected pharmaceuticals.

Therapeutic Group	Pharmaceutical	DDD 1000 inh^−1^ d^−1^	mg inh^−1^ y^−1^	kg y^−1^	Year	Country	Reference
Anx	ALP	17.64 ^a^	6.4 ^a^	302 ^a^	2010	Spain	[27]
		NA	2.9	178	2004	France	
	LOR	19.67 ^a^	17.9	844	2010	Spain	[27]
		NA	9.6	585	2004	France	
		13.3	NA	709	2010	Italy	[5]
Antib	AZI	0.9 ^a^	98.6	4634 ^a^	2010	Spain	[27]
		NA	67.1	4073	2004	France	
		NA	NA	13870	2010	Italy	[34]
		1.3	NA	13870	2010	Italy	[5]
	CIP	1.1 ^a^	401.5	18870 ^a^	2010	Spain	[27]
		NA	200.7	12186	2004	France	
		NA	NA	21672	2010	Italy	[34]
		1.0	NA	21672	2010	Italy	[5]
	CLA	0.6 ^a^	231.0	10864 ^a^	2010	Spain	[27]
		NA	150	12360	2010	Germany	
		NA	232.9	1700	2010	Switzerland	
		NA	276.1	16889	2010	France	
		NA	NA	64470	2010	Italy	[34]
		3.0	NA	64470	2010	Italy	[5]
	ERY	0.1 ^a^	NA	1716a	2010	Spain	[27]
		NA	NA	0.12	2010	Italy	[34]
Lip reg	BEZ	0.6 ^a^	133.0 ^a^	6178 ^a^	2010	Spain	[27]
		NA	475.2	39158	2010	Germany	
		NA	215.6	1574	2010	Switzerland	
		NA	343.4	20852	2004	France	
		NA	66.7	NA	2005	Sweden	
		NA	NA	7600	2001	Italy	[5]
	SIM	NA	282.7 ^a^	13340 ^a^	2010	Spain	[27]
		NA	114.3	6943	2004	France	
Antiepi	CAR	1.2 ^a^	438.0	20595	2010	Spain	[27]
		NA	1010.9	83299	2010	Germany	
		NA	857.5	6260	2010	Switzerland	
		NA	554.3	33364	2010	France	
		NA	463.0	820	2005	Sweden	
		NA	NA	31190	2010	Italy	[34]
		NA	0.61–0.98	NA	2010	Europe	[35]
		NA	NA	31190	2010	Italy	[5]
		NA	NA	88000	2001	Germany	[1]
SSRIs	ESC	0.01 ^a^	38.8	1824 ^a^	2010	Spain	[27]
		NA	0.08	4.6	2004	France	
	FLU	0.02 ^a^	62.0	2914 ^a^	2010	Spain	[27]
		NA	61.6	3740	2004	France	
	PAR	0.02 ^a^	69.4	3264 ^a^	2010	Spain	[27]
		NA	90.8	5515	2004	France	
	SER	0.05 ^a^	102.1	4800 ^a^	2010	Spain	[27]
		NA	102.5	6224	2004	France	
Anti-inf	DIC	7.9 ^a^	369.9	17395 ^a^	2010	Spain	[27]
		NA	953.6	78579	2010	Germany	
		NA	934.1	6819	2010	Switzerland	
		NA	370.1	22640	2010	France	
		NA	375.9	NA	2005	Sweden	
		NA	60–880	NA	2009	Europe	[35]
		4.5	NA	9602	2010	Italy	[5]
		NA	NA	345000	2001	Germany	[1]
	IBU	NA	4647.5	218527	2010	Spain	[27]
		NA	3043.6	250792	2010	Germany	
		NA	3078.2	22471	2010	Switzerland	
		NA	953.8	58353	2010	France	
		NA	NA	7864	2005	Sweden	
		NA	NA	622000	2001	Germany	[1]
	NAP	5.15 ^a^	1205.9	56700 ^a^	2010	Spain	[27]
		NA	614.7	37332	2004	France	
	PARA	NA	22667.7	1065835	2010	Spain	[27]
		NA	54389.5	3303077	2004	France	
		NA	NA	836000	2001	Germany	[1]
Horm	E2	0.894 ^a^		12.6 ^a^	2010	Spain	[27]
	EE2	1.1969 ^a^	0.03	1.2 ^a^	2010	Spain	[27]
		NA	0.58	48.2	2001	Germany	
		NA	0.54	4.0	2000	Switzerland	
		NA	0.11	NA	2005	Sweden	

Anx—anxiolytics, Antib—antibiotics, Lip reg—lipid regulators, Antiepi—antiepileptics, Anti-inf—anti-inflammatories, Horm—hormones, NA—not available, DDD—defined daily dose and SSRIs—selective serotonin reuptake inhibitors. ^a^ Estimated consumption. Data on ZOL, GEM and CIT was not possible to obtain.

**Table 3:** Excretion rates of the selected pharmaceuticals.

Therapeutic Group	Pharmaceutical	Excretion Results	References
Anx	ALP	20	[55]
	LOR	72.5	[56]
	ZOL	0.75	[57]
Antib	AZI	12	[56]
	CIP	60/83.7	[1]
		70	[5]
		70	[56]
	CLA	25	[58]
		25	[25]
	ERY	25	[49]
		10	[58]
		5	[59]
Lip reg	BEZ	72	[60]
		69	[5]
		47.5	[1]
		50	[61]
		45	[62]
	GEM	50	[63]
	SIM	12.5	[1]
		12.5	[62]
Antiepi	CAR	33	[25]
		5	[64]
		3	[29]
		3	[59]
SSRIs	CIT	23	[56]
		12/20	[65]
	ESC	9	[66]
	FLU	5/10/11	[65]
		10	[28]
	SER	0.2	[56]
		0.2	[28]
		0.2	[65]
	PAR	3	[56]
		3	[28]
		3	[65]
Anti-inf	DIC	39	[5]
		15	[1]
		15	[63]
		15	[60]
		12.5	[62]
	IBU	15	[67]
		10	[68]
		10	[61]
		5	[1]
	NAP	10	[25]
		<1	[59]
	PARA	80	[69]
		75	[56]
Horm	E2	5.6	[70]
	EE2	22/26/27/35/42/53/66/68	[71]

Anx—anxiolytics, Antib—antibiotics, Lip reg—lipid regulators, Antiepi—antiepileptics, SSRIs—selective serotonin reuptake inhibitors, Anti-inf—anti-inflammatories and Horm—hormones.

## Supplementary Materials

The following are available online: Table S1: Physicochemical properties of the selected pharmaceuticals (adapted from Chemspider, Drugbank, Pubchem and ECOSARv1.11), Table S2: Occurrence of pharmaceuticals in wastewaters influents (WWIs), Table S3: Occurrence of pharmaceuticals in wastewater efluents (WWEs), Table S4: Occurrence of pharmaceuticals in surface waters (SWs) and Table S5: Occurrence of pharmaceuticals in seawaters (SeaW), groundwaters (GWs), drinking waters (DWs) and mineral waters (DWs).
