# Supplementary material for: Selected Pharmaceuticals in Different Aquatic Compartments: Part I—Source, Fate and Occurrence
Source: Molecules. 2020 Feb 25;25(5):1026. doi: 10.3390/molecules25051026 (PMC7179177; doi:10.3390/molecules25051026)
Supplement: Supplementary file 1 [file molecules-25-01026-s001.pdf]

Supporting information for

Selected pharmaceuticals in different aquatic compartments: Part I - source, fate and occurrence.

André M.P.T. Pereira [a,*], Liliana J.G. Silva [a], Célia S.M. Laranjeiro [a], Celeste Lino [a], Angelina Pena [a]

[a] *LAQV, REQUIMTE, Laboratory of Bromatology and Pharmacognosy, Faculty of Pharmacy, University of Coimbra, Polo III, Azinhaga de St[a] Comba, 3000-548 Coimbra, Portugal*

***Corresponding author:**

LAQV, REQUIMTE, Group of Bromatology, Pharmacognosy and Analytical Sciences, Faculty of Pharmacy, University of Coimbra

Pólo das Ciências da Saúde, Azinhaga de Santa Comba, 3000-548 Coimbra, Portugal.

Tel: +351239488400

Fax : +351239827126

E-mail addresses:

amptpereira@gmail.com (A.M.P.T. Pereira), ljgsilva@hotmail.com (L.J.G. Silva), cmlino@ci.uc.pt (C. Lino), apena@ci.uc.pt (A. Pena)

Table S1. Physicochemical properties of the selected pharmaceuticals (adapted from Chemspider, Drugbank, Pubchem and ECOSARv1.11).

Therapeutic Group/Compound	Cas Number	Structural Formula	Molecular Weight	pKa (DB)	log K_{ow}	log D_{ow} (pH 7.4)	log K_{oc} (pH 7.4)	Solubility (mg L^{-1})
Anxiolitics			307.40–321.16	5.1–18.3	2.41–3.87	2.49–3.06	2.73–3.01	16.6–32.4
Alprazolam (ALP)	28981-97-7	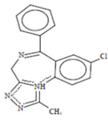 $C_{17}H_{13}ClN_4$	308.77	5.1/18.3	3.87	2.63	2.81	32.4
Lorazepam (LOR)	846-49-1	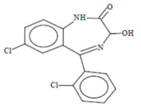 $C_{15}H_{10}Cl_2N_2O_2$	321.16	10.6	2.41	2.49	2.73	16.6
Zolpidem (ZOL)	82626-48-0	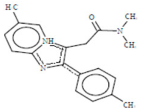 $C_{19}H_{21}N_3O$	307.40	5.65	3.85	3.06	3.01	31.3
Antibiotics			331.35–749.00	5.76–12.46	0.01–3.24	-2.23–1.69	0–2.33	217–1350

**Therapeutic Group/Compound**	**Cas Number**	**Structural Formula**	**Molecular Weight**	**pKa (DB)**	**log K_{ow}**	**log D_{ow} (pH 7.4)**	**log K_{oc} (pH 7.4)**	**Solubility (mg L^{-1})**
Azithromycin (AZI)	83905-01-5	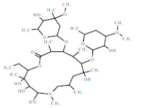 $C_{38}H_{72}N_2O_{12}$	749.00	9.57/12.43	3.24	1.36	1.24	514
Ciprofloxacin (CIP)	85721-33-1	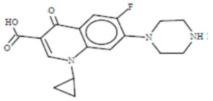 $C_{17}H_{18}FN_3O_3$	331.35	5.76/8.68	0.01	-2.23	0	1350
Clarithromycin (CLA)	81103-11-9	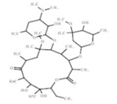 $C_{38}H_{69}NO_{13}$	747.97	8.38/12.46	3.16	0.67	2.33	217
Erythromycin (ERY)	114-07-8	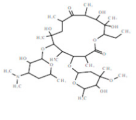 $C_{37}H_{67}NO_{13}$	733.95	8.38/12.44	3.06	1.69	1.96	459
Lipid regulators			250.34–418.58	3.83–14.91	4.25–5.19	-0.11–4.60	0–3.88	1.5–27.8

**Therapeutic Group/Compound**	**Cas Number**	**Structural Formula**	**Molecular Weight**	**pKa (DB)**	**log K_{ow}**	**log D_{ow} (pH 7.4)**	**log K_{oc} (pH 7.4)**	**Solubility (mg L^{-1})**
Bezafibrate (BEZ)	41859-67-0	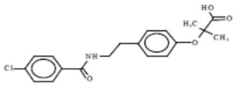 $C_{19}H_{20}ClN_1O_4$	361.83	3.83	4.25	-0.11	0	1.5
Gemfibrozil (GEM)	25812-30-0	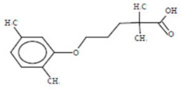 $C_{15}H_{22}O_3$	250.34	4.42	4.77	1.58	1.04	27.8
Simvastatin (SIM)	79902-63-9	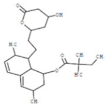 $C_{25}H_{38}O_5$	418.58	14.91	5.19	4.60	3.88	12.2
Antiepileptic								
Carbamazepine (CAR)	298-46-4	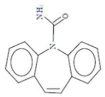 $C_{15}H_{12}N_2O$	236.28	15.96	2.45	2.28	2.62	152
Selective serotonin reuptake inhibitors (SSRIs)			291.06–329.14	9.05–10.5	1.22–4.82	0.5–3.14	0.37–2.16	0.1–58.8

Therapeutic Group/Compound	Cas Number	Structural Formula	Molecular Weight	pKa (DB)	log K_{ow}	log D_{ow} (pH 7.4)	log K_{oc} (pH 7.4)	Solubility (mg L^{-1})
Citalopram (CIT)	59729-33-8	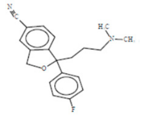 $C_{20}H_{21}FN_2O$	324.16	9.78	1.39	1.27	1.10	58.8
Desmethylcitalopram (N-Cit) (metabolite)	62498-67-3	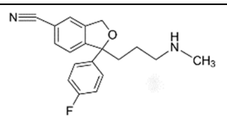 $C_{19}H_{19}FN_2O$	310.15	10.50	3.53	0.50	0.37	57.0
Escitalopram (ESC)								
Fluoxetine (FLU)	54910-89-3	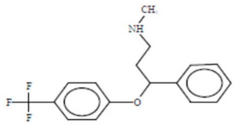 $C_{17}H_{18}F_3NO$	309.13	9.80	1.22	1.75	1.18	1.7
Norfluoxetine (Nor-FLU) (metabolite)	83891-03-6	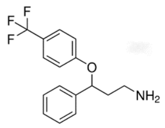 $C_{16}H_{16}F_3NO$	295.12	9.05	4.18	2.23	1.84	35.7

Therapeutic Group/Compound	Cas Number	Structural Formula	Molecular Weight	pKa (DB)	log K_{ow}	log D_{ow} (pH 7.4)	log K_{oc} (pH 7.4)	Solubility (mg L^{-1})
Paroxetine (PAR)	61869-08-7	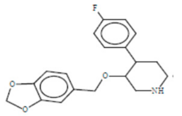 $C_{19}H_{20}FNO_3$	329.14	10.30	1.37	1.46	1.16	35.3
Sertraline (SER)	87857-41-8 79617-96-2	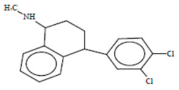 $C_{17}H_{17}Cl_2N$	305.07	9.85	1.37	3.14	2.16	0.1
Desmethylsertraline (Nor-SER) (metabolite)	87857-41-8	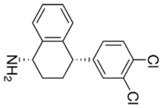 $C_{16}H_{15}Cl_2N$	291.06	9.41	4.82	2.83	2.13	10.6
**Anti-inflammatories**			109.13–312.15	4.0–10.46	0.24–4.02	0.16–1.37	0.29–1.60	4.5–101 200
Diclofenac (DIC)	15307-86-5	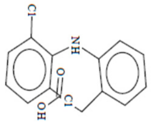 $C_{14}H_{11}Cl_2NO_2$	296.15	4.00	4.02	1.37	0.71	4.5

Therapeutic Group/Compound	Cas Number	Structural Formula	Molecular Weight	pKa (DB)	log K_{ow}	log D_{ow} (pH 7.4)	log K_{oc} (pH 7.4)	Solubility (mg L^{-1})
4-hydroxydiclofenac (4-OH-DIC) (metabolite)	64118-84-9	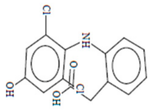 $C_{14}H_{11}Cl_2NO_3$	312.15	-	3.18	1.18	0.59	17.9
Ibuprofen (IBU)	15687-27-1	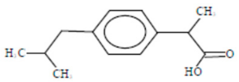 $C_{13}H_{18}O_2$	206.29	4.90	3.80	0.45	0.29	68.4
Naproxen (NAP)	22204-53-1	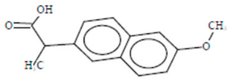 $C_{14}H_{14}O_3$	230.3	4.20	3.50	0.45	0.47	51.1
Paracetamol (PARA)	103-90-2	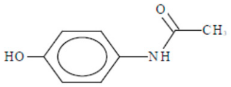 $C_8H_9NO_2$	151.17	9.50	0.27	0.40	1.60	4150
4-aminophenol (4-PARA) (transformation product)	123-30-8	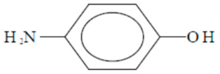 C_6H_7NO	109.13	5.48/10.46	0.24	0.16	1.46	101 200

Therapeutic Group/Compound	Cas Number	Structural Formula	Molecular Weight	pKa (DB)	log K_{ow}	log D_{ow} (pH 7.4)	log K_{oc} (pH 7.4)	Solubility (mg L^{-1})
Hormones			270.37–296.41	10.33	3.43–4.12	3.38–3.87	3.21–3.48	3.9–21.3
Estrone (E1)<br>(natural hormone/metabolite)	53-16-7	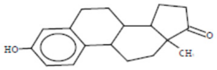<br>$C_{18}H_{22}O_2$	270.37	10.33	3.43	3.38	3.21	3.9
17β-estradiol (E2)	50-28-2	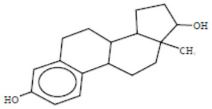<br>$C_{18}H_{24}O_2$	272.39	10.33	3.94	3.62	3.34	21.3
17α-ethinylestradiol (EE2)	57-63-6	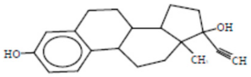<br>$C_{20}H_{24}O_2$	296.41	10.33	4.12	3.87	3.48	6.8

Table S2. Occurrence of pharmaceuticals in wastewaters influents (WWIs).

Pharmaceutical	Country	Year	Sampling Type/Locations	Number Of Samples	Method	LOD/LOQ (ng L^{-1})	Frequency (Highest Value) (%)	Average (Highest Value) (ng L^{-1})	Maximum (Highest Value) (ng L^{-1})	Reference
Anxiolytics										
Alprazolam (ALP)	Belgium	2013	TP/2	10	SPE-LC-HRMS	18/60	0	ND	ND	[1]
	China	2011	GS/3	3	SPE-LC-MS/MS	NA	0	ND	ND	[2]
	China	2011	GS/3	19	SPE-LC-MS/MS	NA/6	5	<LOQ	<LOQ	[3]
	China	2014	TP/5	5	SPE-LC–MS/MS	NA/0.1	100	7.6	8.6	[4]
	India	2013	GS/2	7	SPE-LC-MS/MS	NA/NA	14.5	20.5	69	[5]
	Portugal	2009	GS/2	2	SPE-LC-MS/MS	1/5	0	ND	ND	[6]
	Portugal	2011	TP/1	7	SPE-LC-MS/MS	NA	100	32.3	49.1	[7]
	Portugal	NA	TP/1	48	SPE-LC-MS	NA	35	NA	4705	[8]
	Spain	2008/2009	TP/3	42	SPE-UPLC-MS/MS	0/33	0	ND	ND	[9]
	Spain	2008/2009	TP/3	42	SPE-LC-MS/MS	NA/30	0	ND	ND	[9]
	Spain	2009	TP/3	14	SPE-LC-MS/MS	NA/30	0	ND	ND	[9]
	USA	2013	TP/2	14	SPE-LC-MS/MS	NA	100	6	6	[10]
							29.5(100)	6.0(32.3)	403.1(4705.0)	
Lorazepam (LOR)	China	2011	GS/3	19	SPE-LC-MS/MS	NA/55	63	<LOQ	<LOQ	[3]
	China	2011	GS/3	3	SPE-LC-MS/MS	NA	0	ND	ND	[2]

Pharmaceutical	Country	Year	Sampling Type/Locations	Number Of Samples	Method	LOD/LOQ (ng L^{-1})	Frequency (Highest Value) (%)	Average (Highest Value) (ng L^{-1})	Maximum (Highest Value) (ng L^{-1})	Reference
	China	2014	TP/5	5	SPE-LC–MS/MS	NA/4.8	100	35.8	40.7	[4]
	India	2013	GS/2	7	SPE-LC-MS/MS	NA/NA	78.5	36	95	[5]
	International	NA	NA	NA	NA	NA	NA	ND	ND	[11]
	Portugal	2009	GS/2	2	SPE-LC-MS/MS	2/7	0	ND	ND	[6]
	Portugal	2011	TP/1	7	SPE-LC-MS/MS	NA	100	299	446	[7]
	Portugal	NA	TP/1	48	SPE-LC-MS	NA	0	ND	ND	[8]
	Spain	2007/2009	CS/3	24	SPE-LC-MS/MS	2.1/NA	NA	69	NA	[12]
	Spain	2008/2009	TP/3	42	SPE-UPLC-MS/MS	0/54	0	ND	ND	[9]
	Spain	2008/2009	TP/3	42	SPE-LC-MS/MS	NA/50	0	ND	ND	[9]
	Spain	2009	TP/3	14	SPE-LC-MS/MS	NA/150	0	ND	ND	[9]
	USA	2013	TP/2	14	SPE-LC-MS/MS	NA	72	18	34	[10]
							37.6(100)	35.2(299.0)	51.3(446.0)	
Zolpidem (ZOL)	Portugal	2009	GS/2	2	SPE-LC-MS/MS	1/3	0	ND	ND	[6]
	Portugal	NA	TP/1	48	SPE-LC-MS	NA	0	ND	ND	[8]
	Sweden	2013	FP/1	7	SPE-LC-MS/MS	NA	43	13	NA	[13]
							14.3(43.0)	4.3(13.0)	NA	
Total							31.2(100)	19.9(299.0)	209.7(4705.0)	
Antibiotics										
Azithromycin (AZI)	Croatia	NA	NA/5	5	SPE-LC-MS/MS	7/23	NA	152	300	[14]

Pharmaceutical	Country	Year	Sampling Type/Locations	Number Of Samples	Method	LOD/LOQ (ng L-1)	Frequency (Highest Value) (%)	Average (Highest Value) (ng L-1)	Maximum (Highest Value) (ng L-1)	Reference
	Czech Republic	2011/2012	TP/1	136	SPE-LC-MS/MS	NA/7	99	140	510	[15]
	Greece	2011	FP/1	8	SPE-LC-MS/MS	19/58.5	25	ND	64	[16]
	International	NA	NA	NA	NA	NA	NA	400	3000	[11]
	Italy	NA	TP/1	4	SPE-LC-MS/MS	2/NA	NA	120	NA	[17]
	Japan	2007/2009	NA/9	9	NA	NA	NA	645	NA	[18]
	NA	NA	NA	6	NA	NA	100	260	NA	[19]
	Portugal	2009	GS/2	2	SPE-LC-MS/MS	2/6	100	594	617	[6]
	Portugal	2011	TP/1	7	SPE-LC-MS/MS	NA	100	186	295	[7]
	Portugal	2013/2014	FP/2	18	SPE-UHPLC-MS/MS	0.06/0.18	16.65	18.95	67	[20]
	Portugal	NA	TP/1	48	SPE-LC-MS	3/11	17	NA	210	[8]
	Spain	2011	GS/3	3	SPE-UPLC-MS/MS	3.3/10.9	66	368	437	[21]
	USA	2010	FP/1	6	SPE-LC-MS/MS	3.7/11	NA	<LOQ	280	[22]
							65.5(100)	240.3(645.0)	578.0(3000.0)	
Clarithromycin (CLA)	Czech Republic	2011/2012	TP/1	136	SPE-LC-MS/MS	NA/3	100	1480	3090	[15]
	Greece	2011	FP/1	8	SPE-LC-MS/MS	1.9/5.8	100	1377	2683	[16]
	International	NA	NA	NA	NA	NA	NA	1300	8000	[11]
	Italy	2007/2008	TP/4	24	SPE-LC-MS/MS	NA/NA	NA	476	NA	[23]
	Italy	NA	TP/1	4	SPE-LC-MS/MS	6/NA	NA	200	NA	[17]

Pharmaceutical	Country	Year	Sampling Type/Locations	Number Of Samples	Method	LOD/LOQ (ng L^{-1})	Frequency (Highest Value) (%)	Average (Highest Value) (ng L^{-1})	Maximum (Highest Value) (ng L^{-1})	Reference
	Japan	2007/2009	NA/9	9	NA	NA	NA	964	NA	[18]
	NA	NA	NA	6	NA	NA	100	647	NA	[19]
	Portugal	2011	TP/1	7	SPE-LC-MS/MS	NA	NA	22.2	52.3	[7]
	Portugal	2013/2014	FP/2	18	SPE-UHPLC-MS/MS	0.06/0.20	16.65	24.15	48.2	[20]
	Spain	2007/2009	CS/3	24	SPE-LC-MS/MS	1.5/NA	NA	62	NA	[12]
	Spain	2009	TP/3	14	SPE-LC-MS/MS	NA/10	100	230	620	[9]
	Spain	2011	GS/3	3	SPE-UPLC-MS/MS	16.6/55.5	100	358	632	[21]
	Sweden	2013	FP/1	7	SPE-LC-MS/MS	NA	100	29	NA	[13]
	Taiwan	NA	GS/1	NA	SPE-LC-MS/MS	0.5	NA	324	NA	[24]
	USA	2010	FP/1	6	SPE-LC-MS/MS	3.2/9.6	NA	<LOQ	5.6	[22]
							88.1(100)	499.6(1480.0)	1891.4(8000.0)	
Ciprofloxacin (CIP)	Belgium	2013	TP/2	10	SPE-LC-HRMS	75/250	70	698	NA	[1]
	Czech Republic	2011/2012	TP/1	136	SPE-LC-MS/MS	NA/3	100	410	860	[15]
	Greece	2011	FP/1	8	SPE-LC-MS/MS	5.4/16.3	100	1588	2881	[16]
	Greece	2013/2014	TP/1	24	SPE-LC-MS/MS	18.4/60.7	29.2	152	591	[25]
	International	NA	NA	NA	NA	NA	NA	1600	11 000	[11]
	Italy	2007/2008	TP/4	24	SPE-LC-MS/MS	NA/NA	NA	68	NA	[23]

**Pharmaceutical**	**Country**	**Year**	**Sampling Type/Locations**	**Number Of Samples**	**Method**	**LOD/LOQ (ng L^{-1})**	**Frequency (Highest Value) (%)**	**Average (Highest Value) (ng L^{-1})**	**Maximum (Highest Value) (ng L^{-1})**	**Reference**
	Italy	NA	TP/1	4	SPE-LC-MS/MS	3/NA	NA	2200	NA	[17]
	Kenya	2012/2013	GS/3	3	SPE-LC-MS	NA/NA	66.6	136.6	270	[26]
	Korea	2007	GS/10	10	SPE-LC-MS/MS	NA	0	ND	ND	[27]
	Korea	2008	GS/12	30	SPE-LC-MS/MS	NA	10	182	246	[28]
	NA	NA	NA	20	NA	NA	83	413	571	[19]
	Portugal	2007	TP/1	2	SPE-LC-FD	17/NA	100	433.0	418.8	[29]
	Portugal	2009	GS/2	2	SPE-LC-MS/MS	2/6	0	ND	ND	[6]
	Portugal	2011	TP/1	7	SPE-LC-MS/MS	NA	100	221	330	[7]
	Portugal	2013/2014	FP/2	18	SPE-UHPLC-MS/MS	28.1/84.2	38.9	196	251	[20]
	Portugal	NA	TP/1	48	SPE-LC-MS	1/3	10	NA	15 397	[8]
	Spain	2009	TP/3	14	SPE-LC-MS/MS	NA/320	100	2450	3850	[9]
	Spain	2011	GS/3	3	SPE-UPLC-MS/MS	13.6/34.5	100	414	613	[21]
	Spain	NA	NA/1	12	SPE-LC-MS/MS	NA	NA	5524	13 625	[30]
	Sweden	2002/2003	FP/5	10	SPE-LC-MS	NA/6	100	158	300	[31]
	Sweden	2013	FP/1	7	SPE-LC-MS/MS	NA	71	196	NA	[13]
	Taiwan	NA	GS/1	NA	SPE-LC-MS/MS	0.5	NA	157	NA	[24]
	USA	2010	FP/1	6	SPE-LC-MS/MS	3.3/6.7	NA	<LOQ	8.7	[22]

Pharmaceutical	Country	Year	Sampling Type/Locations	Number Of Samples	Method	LOD/LOQ (ng L^{-1})	Frequency (Highest Value) (%)	Average (Highest Value) (ng L^{-1})	Maximum (Highest Value) (ng L^{-1})	Reference
	USA	NA	CS/7	10	SPE-LC-MS	NA/50	40	150	210	[32]
							62.2(100)	754.2(5524.0)	2706.4(15 397)	
Erythromycin (ERY)	Croatia	NA	NA/5	5	SPE-LC-MS/MS	6/20	NA	ND	ND	[14]
	Czech Republic	2011/2012	TP/1	136	SPE-LC-MS/MS	NA/6	100	110	350	[15]
	Greece	2011	FP/1	8	SPE-LC-MS/MS	155/470	0	ND	ND	[16]
	Greece	2013/2014	TP/1	24	SPE-LC-MS/MS	32.8/108.2	83.3	70.8	320	[25]
	International	NA	NA	NA	NA	NA	NA	NA	10 000	[33]
	International	NA	NA	NA	NA	NA	NA	1800	10 000	[11]
	Italy	2007/2008	TP/4	24	SPE-LC-MS/MS	NA/NA	NA	0.9	NA	[23]
	Italy	NA	TP/1	4	SPE-LC-MS/MS	7/NA	NA	46	NA	[17]
	Japan	2007/2009	NA/9	9	NA	NA	NA	212	NA	[18]
	Korea	2007	GS/10	10	SPE-LC-MS/MS	NA	NA	NA	1050	[27]
	Korea	2008	GS/12	30	SPE-LC-MS/MS	NA	47	23	46	[28]
	NA	NA	NA	3	NA	NA	100	413	571	[19]
	Portugal	2011	TP/1	7	SPE-LC-MS/MS	NA	100	92.7	220	[7]
	South Africa	2013	GS/3	3	SPE-LC-MS/MS	3/12	NA	1130	NA	[34]
	Spain	2009	TP/3	14	SPE-LC-MS/MS	NA/20	0	ND	ND	[9]
	Spain	NA	NA/1	12	SPE-LC-MS/MS	NA	NA	346	2310	[30]

Pharmaceutical	Country	Year	Sampling Type/Locations	Number Of Samples	Method	LOD/LOQ (ng L^{-1})	Frequency (Highest Value) (%)	Average (Highest Value) (ng L^{-1})	Maximum (Highest Value) (ng L^{-1})	Reference
	Sweden	2002/2003	FP/5	10	SPE-LC-MS	NA/160	0	ND	ND	[31]
	Taiwan	NA	GS/1	NA	SPE-LC-MS/MS	0.1	NA	319	NA	[24]
	UK	2007	GS/2	20	SPE-LC-MS/MS	NA	100	2070	10 025	[35]
	UK	NA	NA	NA	NA	NA	NA	NA	2530	[36]
	USA	2010	TP/1	6	SPE-LC-MS/MS	2.2/7.3	0	ND	ND	[37]
	USA	NA	CS/7	10	SPE-LC-MS	NA/50	60	340	1200	[32]
							53.7(100)	367.0(2070.0)	2413.9(10 025)	
Total							64.8(100)	502.9(5524.0)	2093.5(15 397)	
Lipid regulators										
Bezafibrate (BEZ)	Brazil	1997	TP/1	6	SPE-GC-MS	NA	NA	1200	NA	[38]
	China	2009/2010	GS/2	24	SPE-UPLC-MS/MS	NA	100	72.6	159	[39]
	Croatia	NA	NA/5	5	SPE-LC-MS/MS	9/31	NA	23	50	[14]
	Greece	2010/2011	TP/8	32	SPE-LC-MS/MS	NA	66	137	946	[40]
	Greece	2013/2014	TP/1	24	SPE-LC-MS/MS	7.9/26.1	8.3	<LOD	51.8	[25]
	International	NA	NA	NA	NA	NA	NA	NA	1390	[33]
	International	NA	NA	NA	NA	NA	NA	3500	15 000	[11]
	Mexico	2015/2016	TP/2	8	SPE- LC-MS/MS	4.9/16	100	2342.5	3445	[41]
	NA	NA	NA	25	NA	NA	100	2400	7600	[19]
	Portugal	2009	GS/2	2	SPE-LC-MS/MS	5/18	100	3283	5217	[6]
	Portugal	2011	TP/1	7	SPE-LC-MS/MS	NA	100	490	623	[7]

Pharmaceutical	Country	Year	Sampling Type/Locations	Number Of Samples	Method	LOD/LOQ (ng L^{-1})	Frequency (Highest Value) (%)	Average (Highest Value) (ng L^{-1})	Maximum (Highest Value) (ng L^{-1})	Reference
	Spain	2007/2009	CS/3	24	SPE-LC-MS/MS	1.2/NA	NA	381	NA	[12]
	Spain	2008/2009	TP/3	42	SPE-LC-MS/MS	NA/20	100	160	460	[9]
	Spain	2009	TP/3	14	SPE-LC-MS/MS	NA/30	100	80	100	[9]
	Spain	2012	GS/3	21	SPE-UPLC-MS/MS	NA/5.0	100	53	59.1	[42]
	Spain	NA	NA/1	12	SPE-LC-MS/MS	NA	NA	141	361	[30]
	UK	2007	GS/2	20	SPE-LC-MS/MS	NA	100	429	1391	[35]
	UK	NA	NA	NA	NA	NA	NA	NA	971	[43]
							88.6(100)	918.3(3500)	2364.0(15 000)	
Gemfibrozil (GEM)	Brazil	1997	TP/1	6	SPE-GC-MS	NA	NA	350	NA	[38]
	Canada	2004	TP/8	8	SPE-GC-MS/MS	10/NA	100	260	36 530	[44]
	China	2009/2010	GS/2	24	SPE-UPLC-MS/MS	NA	100	116	359	[39]
	China	2012/2013	GS/1	4	SPE-LC-MS/MS	0.1/NA	NA	70	NA	[45]
	China	2014	GS/3	12	SPE-LC-MS/MS	0.1/NA	100	7.2	26.6	[46]
	Croatia	NA	NA/5	5	SPE-LC-MS/MS	3/9	NA	155	360	[14]
	Greece	2010/2011	TP/8	32	SPE-LC-MS/MS	NA	66	174	899	[40]
	Greece	2011	FP/1	8	SPE-LC-MS/MS	2.6/7.9	100	348	753	[16]
	Greece	2013/2014	TP/1	24	SPE-LC-MS/MS	38.7/127.7	0	ND	ND	[25]
	International	NA	NA	NA	NA	NA	NA	NA	17 100	[33]

**Pharmaceutical**	**Country**	**Year**	**Sampling Type/Locations**	**Number Of Samples**	**Method**	**LOD/LOQ (ng L^{-1})**	**Frequency (Highest Value) (%)**	**Average (Highest Value) (ng L^{-1})**	**Maximum (Highest Value) (ng L^{-1})**	**Reference**
	International	NA	NA	NA	NA	NA	NA	2400	12 000	[11]
	Korea	2007	GS/10	10	SPE-LC-MS/MS	NA	NA	NA	20	[27]
	Korea	2010	NA/5	5	SPE-LC-MS/MS	NA	100	222	318	[47]
	Mexico	2015/2016	TP/2	8	SPE- HPLC-MS/MS	11/38	100	111.5	225	[41]
	NA	NA	NA	4	NA	NA	25	1630	3000	[19]
	Portugal	2009	GS/2	2	SPE-LC-MS/MS	11/40	100	299	307	[6]
	Portugal	2011	TP/1	7	SPE-LC-MS/MS	NA	NA	<LOQ	22.5	[7]
	Spain	2007/2009	CS/3	24	SPE-LC-MS/MS	0.9/NA	NA	144	NA	[12]
	Spain	2008/2009	TP/3	42	SPE-LC-MS/MS	NA/50	100	1110	2120	[9]
	Spain	2009	TP/3	14	SPE-LC-MS/MS	NA/70	100	210	540	[9]
	Spain	2012	GS/3	21	SPE-UPLC-MS/MS	NA/10	100	336	371.8	[42]
	Spain	NA	NA/1	12	SPE-LC-MS/MS	NA	NA	3525	17 055	[30]
	Sweden	2002	FP/1	1	SPE-GC/LC-MS/MS	NA	100	710	710	[48]
	USA	2010	FP/1	6	SPE-LC-MS/MS	1.6/4.8	100	180	1200	[22]
							86.1(100)	561.7(3525.0)	4472.2(36 530)	
Simvastatin (SIM)	Canada	2002	NA/1	3	SPE-LC-MS/MS	1.0/NA	NA	4	NA	[49]
	Greece	2010/2011	TP/8	32	SPE-LC-MS/MS	NA	34	20	91	[40]

Pharmaceutical	Country	Year	Sampling Type/Locations	Number Of Samples	Method	LOD/LOQ (ng L-1)	Frequency (Highest Value) (%)	Average (Highest Value) (ng L-1)	Maximum (Highest Value) (ng L-1)	Reference
	Greece	2011	FP/1	8	SPE-LC-MS/MS	28/84.2	38	147	914	[16]
	Greece	2013/2014	TP/1	24	SPE-LC-MS/MS	11.6/38.3	8.3	49.1	718	[25]
	International	NA	NA	NA	NA	NA	NA	4	4	[11]
	Malaysia	2013	GS/4	12	SPE-LC-MS/MS	NA/15	50	27	35	[50]
	Portugal	2009	GS/2	2	SPE-LC-MS/MS	8/29	50	976	976	[6]
	Spain	2009	TP/3	14	SPE-LC-MS/MS	NA/130	0	ND	ND	[9]
	UK	2007	GS/2	20	SPE-LC-MS/MS	NA	29	115	798	[35]
	UK	NA	NA	NA	NA	NA	NA	NA	115	[43]
							29.9(50.0)	149.1(976.0)	405.7(976.0)	
Total							75.0(100)	604.1(3525.0)	2943.3(36 530)	
Antiepileptic										
Carbamazepine (CAR)	Belgium	2013	TP/2	10	SPE-LC-HRMS	39/129	100	609.6	NA	[1]
	Canada	NA	TP/1	NA	SPE-LDTD-APCI-MS/MS	54/108	NA	305	NA	[51]
	China	2009/2010	GS/2	24	SPE-UPLC-MS/MS	NA	100	70.7	233	[39]
	China	2011	GS/3	19	SPE-LC-MS/MS	NA/0.3	84	NA	23.7	[3]
	China	2011	GS/3	3	SPE-LC-MS/MS	NA	100	21.3	23	[2]
	China	2012/2013	GS/1	4	SPE-LC-MS/MS	0.1/NA	NA	50	NA	[45]
	China	2014	GS/3	12	SPE-LC-MS/MS	0.1/NA	75	3.9	15.1	[46]

Pharmaceutical	Country	Year	Sampling Type/Locations	Number Of Samples	Method	LOD/LOQ (ng L^{-1})	Frequency (Highest Value) (%)	Average (Highest Value) (ng L^{-1})	Maximum (Highest Value) (ng L^{-1})	Reference
	China	2014	TP/5	5	SPE-LC–MS/MS	NA/2.5	100	45.2	68.2	[4]
	Croatia	NA	NA/5	5	SPE-LC-MS/MS	18/61	NA	420	950	[14]
	Czech Republic	2011/2012	TP/1	136	SPE-LC-MS/MS	NA/8	100	460	710	[15]
	Greece	2010/2011	TP/8	32	SPE-LC-MS/MS	NA	100	76	355	[40]
	Greece	2011	FP/1	8	SPE-LC-MS/MS	21/64.2	100	533	1713	[16]
	Greece	2013/2014	TP/1	24	SPE-LC-MS/MS	2.1/7	100	34.1	114	[25]
	India	2013	GS/2	7	SPE-LC-MS/MS	NA/NA	100	500	750	[5]
	International	NA	NA	NA	NA	NA	NA	NA	3780	[33]
	International	NA	NA	NA	NA	NA	NA	1200	12 000	[11]
	Italy	NA	TP/1	4	SPE-LC-MS/MS	4/NA	NA	570	NA	[17]
	Japan	2001/2003	TP/5	12	SPE-GC-MS	NA	NA	54	270	[52]
	Kenya	2012/2013	GS/3	3	SPE-LC-MS	NA/NA	33.3	116.66	350	[26]
	Korea	2007	GS/10	10	SPE-LC-MS/MS	NA	NA	NA	500	[27]
	Korea	2008	GS/12	30	SPE-LC-MS/MS	NA	100	1920	21 600	[28]
	Korea	2010	NA/5	5	SPE-LC-MS/MS	NA	100	72	127	[47]
	Malaysia	2013	GS/4	12	SPE-LC-MS/MS	NA/22	83	62	182	[50]
	Mexico	2015/2016	TP/2	8	SPE- LC-MS/MS	5.5/18	100	214	380	[41]

Pharmaceutical	Country	Year	Sampling Type/Locations	Number Of Samples	Method	LOD/LOQ (ng L^{-1})	Frequency (Highest Value) (%)	Average (Highest Value) (ng L^{-1})	Maximum (Highest Value) (ng L^{-1})	Reference
	NA	NA	NA	64	NA	NA	100	968	1900	[19]
	Portugal	2011	TP/1	7	SPE-LC-MS/MS	NA	100	565	673	[7]
	Portugal	2013/2014	FP/2	18	SPE-UHPLC-MS/MS	0.06/0.18	100	94.95	226	[20]
	Portugal	NA	GS/5	10	SPE-LC-MS	2/7	20	NA	994	[53]
	Portugal	NA	TP/1	48	SPE-LC-MS	2/7	8	NA	1950	[8]
	South Africa	2013	GS/3	3	SPE-LC-MS/MS	268/891	NA	4560	NA	[34]
	Spain	2003/2004	TP/1	10	SPE-LC-MS/MS	30/NA	100	150	310	[54]
	Spain	2007/2009	CS/3	24	SPE-LC-MS/MS	0.6/NA	NA	591	NA	[12]
	Spain	NA	NA/1	12	SPE-LC-MS/MS	NA	NA	129	173	[30]
	Sweden	2002	FP/1	1	SPE-GC/LC-MS/MS	NA	100	1680	1680	[48]
	Sweden	2013	FP/1	7	SPE-LC-MS/MS	NA	100	284	NA	[13]
	Taiwan	NA	GS/1	NA	SPE-LC-MS/MS	0.1	NA	24.8	NA	[24]
	UK	2007	GS/2	20	SPE-LC-MS/MS	NA	100	1322	3110	[35]
	UK	NA	NA	NA	NA	NA	NA	NA	2593	[43]
	USA	2010	TP/1	6	SPE-LC-MS/MS	86.5/288.3	NA	5	NA	[37]
	USA	2010	FP/1	6	SPE-LC-MS/MS	2.7/8.2	100	72	310	[22]
	USA	2013	TP/2	14	SPE-LC-MS/MS	NA	100	193	588	[10]

Pharmaceutical	Country	Year	Sampling Type/Locations	Number Of Samples	Method	LOD/LOQ (ng L^{-1})	Frequency (Highest Value) (%)	Average (Highest Value) (ng L^{-1})	Maximum (Highest Value) (ng L^{-1})	Reference
							89.0(100)	513.6(4560.0)	1832.8(21 600)	
Selective serotonin reuptake inhibitors (SSRIs)										
Citalopram (CIT)	Canada	2007	FP/1	6	SPE-LC-MS/MS	0.077/NA	100	52.5	52.7	[55]
	China	2011	GS/3	19	SPE-LC-MS/MS	NA/0.1	100	NA	3.5	[3]
	China	2011	GS/3	3	SPE-LC-MS/MS	NA	100	2.5	4	[2]
	Czech Republic	2011/2012	TP/1	136	SPE-LC-MS/MS	NA/5	100	83	180	[15]
	Norway	2005	FP/3	3	SPE-LC-MS/MS	NA/0.6	100	257	612	[56]
	Norway	2007	FP/2	3	LPME-LC-MS/MS	0.017/0.057	100	156	303.6	[57]
	Portugal	2011	TP/1	7	SPE-LC-MS/MS	NA	100	23.3	34.3	[7]
	Portugal	2013/2014	FP/2	18	SPE-UHPLC-MS/MS	0.96/2.94	38.85	19.4	35.7	[20]
	Sweden	2013	FP/1	7	SPE-LC-MS/MS	NA	100	266	NA	[13]
	USA	2013	TP/2	14	SPE-LC-MS/MS	NA	100	96	170	[10]
							93.9(100)	106.2(266.0)	155.1(612.0)	
Desmethylcitalopram (N-CIT)	Norway	2007	FP/2	3	LPME-LC-MS/MS	0.229/0.762	100	253	425.7	[57]
	USA	2013	TP/2	14	SPE-LC-MS/MS	NA	50	34	126	[10]
							75.0(100)	143.5(253.0)	275.9(425.7)	
Escitalopram (ESC)	Portugal	NA	TP/1	48	SPE-LC-MS	14/47	21	NA	32 228	[8]

Pharmaceutical	Country	Year	Sampling Type/Locations	Number Of Samples	Method	LOD/LOQ (ng L^{-1})	Frequency (Highest Value) (%)	Average (Highest Value) (ng L^{-1})	Maximum (Highest Value) (ng L^{-1})	Reference
							21.0(21.0)	NA	32 228(32 228)	
Fluoxetine (FLU)	Belgium	2013	TP/2	10	SPE-LC-HRMS	8.6/29	0	ND	ND	[1]
	Canada	2007	FP/1	6	SPE-LC-MS/MS	0.05/NA	100	3.3	3.5	[55]
	China	2011	GS/3	19	SPE-LC-MS/MS	NA/4	68	<LOQ	<LOQ	[3]
	China	2011	GS/3	3	SPE-LC-MS/MS	NA	0	ND	ND	[2]
	China	2012/2013	GS/1	4	SPE-LC-MS/MS	0.1/NA	NA	40	NA	[45]
	China	2014	TP/5	5	SPE-LC–MS/MS	NA/0.2	100	2.6	4.7	[4]
	China	2014	GS/3	12	SPE-LC-MS/MS	0.1/NA	0	ND	ND	[46]
	Croatia	NA	NA/5	5	SPE-LC-MS/MS	35/100	NA	ND	ND	[14]
	England	NA	GS/7	109	SPE-LC-MS/MS	NA	100	31.3	175.9	[43]
	International	NA	NA	NA	NA	NA	NA	540	4000	[11]
	Norway	2005	FP/3	3	SPE-LC-MS/MS	NA/0.12	100	1.4	2.4	[56]
	Norway	2007	FP/2	3	LPME-LC-MS/MS	0.150/0.490	100	8.4	18.7	[57]
	Portugal	2009	GS/2	2	SPE-LC-MS/MS	1/5	0	ND	ND	[6]
	Portugal	2011	TP/1	7	SPE-LC-MS/MS	NA	NA	<LOQ	29.7	[7]
	Portugal	2013/2014	FP/2	18	SPE-UHPLC-MS/MS	0.08/0.24	100	8.77	17.8	[20]
	Portugal	NA	GS/5	10	SPE-LC-MS	15/57	50	NA	1704	[53]

Pharmaceutical	Country	Year	Sampling Type/Locations	Number Of Samples	Method	LOD/LOQ (ng L^{-1})	Frequency (Highest Value) (%)	Average (Highest Value) (ng L^{-1})	Maximum (Highest Value) (ng L^{-1})	Reference
	Portugal	NA	TP/1	48	SPE-LC-MS	17/57	42	NA	3465	[8]
	Spain	NA	NA/1	12	SPE-LC-MS/MS	NA	NA	585	1827	[30]
	Sweden	2013	FP/1	7	SPE-LC-MS/MS	NA	0	ND	ND	[13]
	UK	NA	NA	NA	NA	NA	NA	NA	86	[36]
	USA	2010	FP/1	6	SPE-LC-MS/MS	3.5/10.5	100	20	95	[22]
							57.3(100)	68.9(585.0)	571.5(4000.0)	
Norfluoxetine (Nor-FLU)	Canada	2007	FP/1	6	SPE-LC-MS/MS	0.087/NA	100	3.0	4.2	[55]
	England	NA	GS/7	109	SPE-LC-MS/MS	NA	97	15.4	118.0	[43]
	International	NA	NA	NA	NA	NA	NA	12	12	[11]
	Norway	2007	FP/2	3	LPME-LC-MS/MS	0.160/0.540	100	4.2	9.3	[57]
	Portugal	2011	TP/1	7	SPE-LC-MS/MS	NA	100	112	226	[7]
	Portugal	2013/2014	FP/2	18	SPE-UHPLC-MS/MS	0.28/0.82	11.1	48.2	51.2	[20]
	UK	NA	NA	NA	NA	NA	NA	NA	63	[36]
							81.6(100)	32.5(112.0)	69.1(226.0)	
Paroxetine (PAR)	Belgium	2013	TP/2	10	SPE-LC-HRMS	202/674	0	ND	ND	[1]
	Canada	2007	FP/1	6	SPE-LC-MS/MS	0.096/NA	100	5.0	5.3	[55]
	China	2011	GS/3	19	SPE-LC-MS/MS	NA/60	0	ND	ND	[3]
	China	2011	GS/3	3	SPE-LC-MS/MS	NA	0	ND	ND	[2]

Pharmaceutical	Country	Year	Sampling Type/Locations	Number Of Samples	Method	LOD/LOQ (ng L^{-1})	Frequency (Highest Value) (%)	Average (Highest Value) (ng L^{-1})	Maximum (Highest Value) (ng L^{-1})	Reference
	Croatia	NA	NA/5	5	SPE-LC-MS/MS	6/22	NA	ND	ND	[14]
	International	NA	NA	NA	NA	NA	NA	16	16	[11]
	Norway	2005	FP/3	3	SPE-LC-MS/MS	NA/0.12	100	5.1	12.3	[56]
	Norway	2007	FP/2	3	LPME-LC-MS/MS	0.053/0.205	100	9.1	12.9	[57]
	Portugal	2009	GS/2	2	SPE-LC-MS/MS	1/3	100	75	105	[6]
	Portugal	2011	TP/1	7	SPE-LC-MS/MS	NA	0	ND	ND	[7]
	Portugal	2013/2014	FP/2	18	SPE-UHPLC-MS/MS	4.32/13.1	16.65	22.8	23.6	[20]
	Portugal	NA	GS/5	10	SPE-LC-MS	27/89	30	NA	1312	[53]
	Portugal	NA	TP/1	48	SPE-LC-MS	27/89	44	NA	39 732	[8]
	Spain	2008/2009	TP/3	42	SPE-LC-MS/MS	NA/200	0	ND	ND	[9]
	Spain	2009	TP/3	14	SPE-LC-MS/MS	NA/270	0	ND	ND	[9]
							37.7(100)	10.2(75.0)	2747.9(39 732)	
Sertraline (SER)	Canada	2007	FP/1	6	SPE-LC-MS/MS	0.048/NA	100	6.1	6.1	[55]
	China	2011	GS/3	19	SPE-LC-MS/MS	NA/8	95	<LOQ	<LOQ	[3]
	China	2011	GS/3	3	SPE-LC-MS/MS	NA	0	ND	ND	[2]
	Czech Republic	2011/2012	TP/1	136	SPE-LC-MS/MS	NA/3	70	12	27	[15]
	Greece	2013/2014	TP/1	24	SPE-LC-MS/MS	1.5/4.9	20.8	<LOD	21.0	[25]

Pharmaceutical	Country	Year	Sampling Type/Locations	Number Of Samples	Method	LOD/LOQ (ng L^{-1})	Frequency (Highest Value) (%)	Average (Highest Value) (ng L^{-1})	Maximum (Highest Value) (ng L^{-1})	Reference
	India	2013	GS/2	7	SPE-LC-MS/MS	NA/NA	64	31.5	91	[5]
	Norway	2005	FP/3	3	SPE-LC-MS/MS	NA/0.29	100	2.1	2.5	[56]
	Norway	2007	FP/2	3	LPME-LC-MS/MS	0.160/0.520	100	12.5	19.8	[57]
	Portugal	2011	TP/1	7	SPE-LC-MS/MS	NA	0	ND	ND	[7]
	Portugal	2013/2014	FP/2	18	SPE-UHPLC-MS/MS	0.34/1.02	5.55	NA	NA	[20]
	Portugal	NA	TP/1	48	SPE-LC-MS	NA	0	ND	ND	[8]
	Sweden	2013	FP/1	7	SPE-LC-MS/MS	NA	100	36	NA	[13]
	USA	2013	TP/2	14	SPE-LC-MS/MS	NA	100	62	114	[10]
							58.1(100)	13.5(62.0)	25.6(114.0)	
Desmethylsertraline (Nor-SER)	Canada	2007	FP/1	6	SPE-LC-MS/MS	0.072/NA	100	4.6	5	[55]
	Norway	2007	FP/2	3	LPME-LC-MS/MS	0.618/4.1	33	30.5	30.6	[57]
	USA	2013	TP/2	14	SPE-LC-MS/MS	NA	100	68	183	[10]
							77.7(100)	34.4(68.0)	72.9(183.0)	
Total							62.2(100)	48.8(585.0)	1291.3(39 732)	
Anti-inflammatories										
Diclofenac (DIC)	Belgium	2013	TP/2	10	SPE-LC-HRMS	35/118	100	1073	NA	[1]
	Brazil	1997	TP/1	6	SPE-GC-MS	NA	NA	800	NA	[38]
	Canada	2004	TP/8	8	SPE-GC-MS/MS	10/NA	100	170	2450	[44]

Pharmaceutical	Country	Year	Sampling Type/Locations	Number Of Samples	Method	LOD/LOQ (ng L^{-1})	Frequency (Highest Value) (%)	Average (Highest Value) (ng L^{-1})	Maximum (Highest Value) (ng L^{-1})	Reference
	Canada	NA	TP/1	NA	SPE-LDTD-APCI-MS/MS	70/196	NA	256	NA	[51]
	China	2009/2010	GS/2	24	SPE-UPLC-MS/MS	NA	100	286	438	[39]
	China	2012/2013	GS/1	4	SPE-LC-MS/MS	0.1/NA	NA	300	NA	[45]
	China	2014	GS/3	12	SPE-LC-MS/MS	0.1/NA	100	49	71.8	[46]
	Croatia	NA	NA/5	5	SPE-LC-MS/MS	10/39	100	250	540	[14]
	Greece	1998/1999	NA/4	11	SPE-GC-MS	1/2	0	0	0	[58]
	Greece	2009	FP/2	18	SPE-GC-MS	NA	NA	1410	2170	[59]
	Greece	2010/2011	TP/8	32	SPE-LC-MS/MS	NA	94	249	5164	[40]
	Greece	2011	FP/1	8	SPE-LC-MS/MS	21/63.6	100	738	1001	[16]
	Greece	2013/2014	TP/1	24	SPE-LC-MS/MS	18.9/62.4	37.5	413	4869	[25]
	International	NA	NA	NA	NA	NA	NA	NA	94 200	[33]
	International	NA	NA	NA	NA	NA	NA	1000	10 000	[11]
	Kenya	2012/2013	GS/3	3	SPE-LC-MS	NA/NA	100	1186.6	1510	[26]
	Korea	2007	GS/10	10	SPE-LC-MS/MS	NA	NA	NA	30	[27]
	Korea	2008	GS/12	30	SPE-LC-MS/MS	NA	20	237	523	[28]
	Korea	2010	NA/5	5	SPE-LC-MS/MS	NA	100	131	243	[47]
	Luxembourg	2007/2008	TP/1	24	SPE-LC-MS/MS	0.3/1.0	100	NA	43	[60]

Pharmaceutical	Country	Year	Sampling Type/Locations	Number Of Samples	Method	LOD/LOQ (ng L-1)	Frequency (Highest Value) (%)	Average (Highest Value) (ng L-1)	Maximum (Highest Value) (ng L-1)	Reference
	Malaysia	2013	GS/4	12	SPE-LC-MS/MS	NA/182	42	2008	5048	[50]
	Mexico	2015/2016	TP/2	8	SPE- LC-MS/MS	5.5/18	100	1726.5	2470	[41]
	NA	NA	NA	91	NA	NA	81	1340	4110	[19]
	Portugal	2009	GS/2	2	SPE-LC-MS/MS	18/84	100	1014	1597	[6]
	Portugal	2011	TP/1	7	SPE-LC-MS/MS	NA	NA	69.7	269	[7]
	Portugal	2013/2014	FP/2	18	SPE-UHPLC-MS/MS	1.40/4.40	5.55	486	972	[20]
	Portugal	NA	GS/5	10	SPE-LC-MS	7/24	70	NA	6674	[53]
	Portugal	NA	TP/1	48	SPE-LC-MS	7/24	96	NA	64 479	[8]
	Spain	2003/2004	TP/1	10	SPE-LC-MS/MS	100/NA	100	1500	3600	[54]
	Spain	2007/2009	CS/3	24	SPE-LC-MS/MS	1.2/NA	NA	1002	NA	[12]
	Spain	2008/2009	TP/3	42	SPE-LC-MS/MS	NA/140	100	560	1490	[9]
	Spain	2009	TP/3	14	SPE-LC-MS/MS	NA/140	36	530	790	[9]
	Spain	2012	GS/3	21	SPE-UPLC-MS/MS	NA/20	100	21	121.8	[42]
	Spain	NA	NA	NA	SPE-GC-MS	NA/100	NA	NA	3600	[61]
	Spain	NA	NA/1	12	SPE-LC-MS/MS	NA	NA	232	561	[30]
	Spain/Belgium/Germany	NA	TP/NA	14	SPE-GC/LC-MS/MS	7/20	NA	NA	148	[62]
	Switzerland	2003/2004	FP/5	44	SPE-GC-MS	6/20	100	1300	1900	[63]

Pharmaceutical	Country	Year	Sampling Type/Locations	Number Of Samples	Method	LOD/LOQ (ng L^{-1})	Frequency (Highest Value) (%)	Average (Highest Value) (ng L^{-1})	Maximum (Highest Value) (ng L^{-1})	Reference
	Sweden	2002	FP/1	1	SPE-GC/LC-MS/MS	NA	100	160	160	[48]
	Sweden	2013	FP/1	7	SPE-LC-MS/MS	NA	100	615	NA	[13]
	Taiwan	NA	GS/1	NA	SPE-LC-MS/MS	1	NA	57.8	NA	[24]
	UK	2007	GS/2	20	SPE-LC-MS/MS	NA	100	165	1161	[35]
	UK	NA	NA	NA	NA	NA	NA	NA	1500	[36]
							81.5(100)	627.5(2008.0)	6397.2(94 200)	
Ibuprofen (IBU)	Belgium	2013	TP/2	10	SPE-LC-HRMS	312/1051	90	6993	NA	[1]
	Brazil	1997	TP/1	6	SPE-GC-MS	NA	NA	350	NA	[38]
	Canada	2004	TP/8	8	SPE-GC-MS/MS	10/NA	100	6770	10 210	[44]
	China	2012/2013	GS/1	4	SPE-LC-MS/MS	0.1/NA	NA	900	NA	[45]
	China	2014	GS/3	12	SPE-LC-MS/MS	0.1/NA	100	300	406	[46]
	Croatia	NA	NA/5	5	SPE-LC-MS/MS	12/20	NA	516	900	[14]
	Greece	1998/1999	NA/4	11	SPE-GC-MS	0.6/1.6	100	NA	560	[58]
	Greece	2009	FP/2	18	SPE-GC-MS	NA	NA	820	1440	[59]
	Greece	2010/2011	TP/8	32	SPE-LC-MS/MS	NA	59	711	8890	[40]
	Greece	2011	FP/1	8	SPE-LC-MS/MS	16/47	100	1269	1928	[16]
	India	2013	GS/2	7	SPE-LC-MS/MS	NA/NA	85.5	1300	2800	[5]
	International	NA	NA	NA	NA	NA	NA	NA	603 000	[33]

Pharmaceutical	Country	Year	Sampling Type/Locations	Number Of Samples	Method	LOD/LOQ (ng L-1)	Frequency (Highest Value) (%)	Average (Highest Value) (ng L-1)	Maximum (Highest Value) (ng L-1)	Reference
	International	NA	NA	NA	NA	NA	NA	37 000	700 000	[11]
	Japan	2001/2003	TP/5	12	SPE-GC-MS	NA	NA	672	1130	[52]
	Kenya	2012/2013	GS/3	3	SPE-LC-MS	NA/NA	100	8400	10550	[26]
	Korea	2007	GS/10	10	SPE-LC-MS/MS	NA	NA	NA	1050	[27]
	Korea	2008	GS/12	30	SPE-LC-MS/MS	NA	0	ND	ND	[28]
	Korea	2010	NA/5	5	SPE-LC-MS/MS	NA	100	2265	2853	[47]
	Luxembourg	2007/2008	TP/1	24	SPE-LC-MS/MS	0.3/1.0	100	NA	3080	[60]
	Mexico	2015/2016	TP/2	8	SPE- LC-MS/MS	12/39	NA	1396.5	2835	[41]
	NA	NA	NA	101	NA	NA	97	14 600	83 500	[19]
	Portugal	2009	GS/2	2	SPE-LC-MS/MS	65/219	100	2790	3588	[6]
	Portugal	2011	TP/1	7	SPE-LC-MS/MS	NA	NA	1596	496	[7]
	Portugal	2013/2014	FP/2	18	SPE-UHPLC-MS/MS	32.9/110	100	11994.5	24505	[20]
	Portugal	NA	GS/5	10	SPE-LC-MS	14/46	80	NA	106 490	[53]
	Portugal	NA	TP/1	48	SPE-LC-MS	14/46	60	NA	40 168	[8]
	South Africa	2013	GS/3	3	SPE-LC-MS/MS	244/813	NA	5760	NA	[34]
	Spain	2003/2004	TP/1	10	SPE-LC-MS/MS	23/NA	100	8400	168 000	[54]
	Spain	2008/2009	TP/3	42	SPE-LC-MS/MS	NA/640	98	12 400	39 800	[9]

Pharmaceutical	Country	Year	Sampling Type/Locations	Number Of Samples	Method	LOD/LOQ (ng L^{-1})	Frequency (Highest Value) (%)	Average (Highest Value) (ng L^{-1})	Maximum (Highest Value) (ng L^{-1})	Reference
	Spain	2009	TP/3	14	SPE-LC-MS/MS	NA/730	100	14 600	19 100	[9]
	Spain	2011/2012	NA/1	5	SPE-LC-MS/MS	20.0/66.5	100	10 728	13 740	[64]
	Spain	2012	GS/3	21	SPE-UPLC-MS/MS	NA/30	100	4374	9938.3	[42]
	Spain	NA	NA/1	12	SPE-LC-MS/MS	NA	NA	2687	4113	[30]
	Spain/Belgium/Germany	NA	TP/NA	14	SPE-GC/LC-MS/MS	12/42	NA	NA	860	[62]
	Sweden	2002	FP/1	1	SPE-GC/LC-MS/MS	NA	100	3590	3590	[48]
	Sweden	2013	FP/1	7	SPE-LC-MS/MS	NA	NA	7755	NA	[13]
	Switzerland	2003/2004	FP/4	44	SPE-GC-MS	8/30	100	2680	3100	[63]
	Taiwan	NA	GS/1	NA	SPE-LC-MS/MS	5	NA	2500	NA	[24]
	UK	2007	GS/2	20	SPE-LC-MS/MS	NA	100	1988	6328	[35]
	UK	NA	NA	NA	NA	NA	NA	NA	33 764	[36]
	USA	2010	FP/1	6	SPE-LC-MS/MS	4.7/14	100	2100	11 000	[22]
							90.8(100)	5459.9(37 000)	54 963.2(700 000)	
Naproxen (NAP)	Belgium	2013	TP/2	10	SPE-LC-HRMS	689/2295	24	3068	NA	[1]
	Brazil	1997	TP/1	6	SPE-GC-MS	NA	NA	600	NA	[38]
	Canada	2004	TP/8	8	SPE-GC-MS/MS	10/NA	100	2760	6030	[44]

Pharmaceutical	Country	Year	Sampling Type/Locations	Number Of Samples	Method	LOD/LOQ (ng L^{-1})	Frequency (Highest Value) (%)	Average (Highest Value) (ng L^{-1})	Maximum (Highest Value) (ng L^{-1})	Reference
	China	2012/2013	GS/1	4	SPE-LC-MS/MS	0.1/NA	NA	60	NA	[45]
	China	2014	GS/3	12	SPE-LC-MS/MS	0.1/NA	92	13	30.6	[46]
	Croatia	NA	NA/5	5	SPE-LC-MS/MS	9/32	NA	99	190	[14]
	Greece	2009	FP/2	18	SPE-GC-MS	NA	NA	935	2670	[59]
	Greece	2010/2011	TP/8	32	SPE-LC-MS/MS	NA	72	856	5810	[40]
	Greece	2011	FP/1	8	SPE-LC-MS/MS	16/47	100	942	1363	[16]
	Greece	2013/2014	TP/1	24	SPE-LC-MS/MS	23.5/77.6	37.5	133	648	[25]
	International	NA	NA	NA	NA	NA	NA	NA	52 900	[33]
	International	NA	NA	NA	NA	NA	NA	6000	100 000	[11]
	Japan	2001/2003	TP/5	12	SPE-GC-MS	NA	NA	100	230	[52]
	Korea	2007	GS/10	10	SPE-LC-MS/MS	NA	NA	NA	1050	[27]
	Korea	2008	GS/12	30	SPE-LC-MS/MS	NA	57	3810	12 500	[28]
	Korea	2010	NA/5	5	SPE-LC-MS/MS	NA	100	2584	5033	[47]
	Mexico	2015/2016	TP/2	8	SPE- LC-MS/MS	14/46	100	2500	4210	[41]
	NA	NA	NA	45	NA	NA	96	26 400	611 000	[19]
	Portugal	2009	GS/2	2	SPE-LC-MS/MS	14/47	100	3029	3474	[6]
	Portugal	2011	TP/1	7	SPE-LC-MS/MS	NA	100	741	1617	[7]

Pharmaceutical	Country	Year	Sampling Type/Locations	Number Of Samples	Method	LOD/LOQ (ng L^{-1})	Frequency (Highest Value) (%)	Average (Highest Value) (ng L^{-1})	Maximum (Highest Value) (ng L^{-1})	Reference
	Portugal	2013/2014	FP/2	18	SPE-UHPLC-MS/MS	0.10/0.40	100	592.5	3245	[20]
	Portugal	NA	GS/5	10	SPE-LC-MS	18/59	20	NA	3894	[53]
	Portugal	NA	TP/1	48	SPE-LC-MS	18/59	15	NA	244	[8]
	Spain	2007/2009	CS/3	24	SPE-LC-MS/MS	6.3/NA	NA	5464	NA	[12]
	Spain	2008/2009	TP/3	42	SPE-LC-MS/MS	NA/50	100	1550	3580	[9]
	Spain	2009	TP/3	14	SPE-LC-MS/MS	NA/80	100	1320	2240	[9]
	Spain	2012	GS/3	21	SPE-UPLC-MS/MS	NA/8	100	2399	5871	[42]
	Spain	NA	NA/1	12	SPE-LC-MS/MS	NA	NA	2363	5228	[30]
	Spain/Belgium/Germany	NA	TP/NA	14	SPE-GC/LC-MS/MS	26/75	NA	NA	455	[62]
	Sweden	2002	FP/1	1	SPE-GC/LC-MS/MS	NA	100	3650	3650	[48]
	Sweden	2013	FP/1	7	SPE-LC-MS/MS	NA	NA	8006	NA	[13]
	Taiwan	NA	GS/1	NA	SPE-LC-MS/MS	5	NA	432	NA	[24]
	UK	2007	GS/2	20	SPE-LC-MS/MS	NA	100	1006	3504	[35]
	UK	NA	NA	NA	NA	NA	NA	NA	1173	[36]
	USA	2010	FP/1	6	SPE-LC-MS/MS	1.0/2.9	100	3000	9400	[22]
							81.6(100)	2910.8(26 400)	29 353.1(611 000)	

Pharmaceutical	Country	Year	Sampling Type/Locations	Number Of Samples	Method	LOD/LOQ (ng L^{-1})	Frequency (Highest Value) (%)	Average (Highest Value) (ng L^{-1})	Maximum (Highest Value) (ng L^{-1})	Reference
Paracetamol (PARA)	Belgium	2013	TP/2	10	SPE-LC-HRMS	1217/4057	100	60 546	ND	[1]
	China	2012/2013	GS/1	4	SPE-LC-MS/MS	0.1/NA	NA	100 000	NA	[45]
	China	2014	GS/3	12	SPE-LC-MS/MS	0.1/NA	100	1900	3480	[46]
	Croatia	NA	NA/5	5	SPE-LC-MS/MS	10/58	100	10 194	26 090	[14]
	Greece	2010/2011	TP/8	32	SPE-LC-MS/MS	NA	88	6465	65 403	[40]
	Greece	2011	FP/1	8	SPE-LC-MS/MS	23/64.2	100	29 635	81 016	[16]
	Greece	2013/2014	TP/1	24	SPE-LC-MS/MS	15.2/50	100	1422	2452	[25]
	International	NA	NA	NA	NA	NA	NA	NA	56 900	[33]
	International	NA	NA	NA	NA	NA	NA	38 000	500 000	[11]
	Kenya	2012/2013	GS/3	3	SPE-LC-MS	NA/NA	100	110887	161290	[26]
	Korea	2007	GS/10	10	SPE-LC-MS/MS	NA	NA	NA	10 000	[27]
	Korea	2008	GS/12	30	SPE-LC-MS/MS	NA	97	13 200	51 900	[28]
	Korea	2010	NA/5	5	SPE-LC-MS/MS	NA	100	7460	10 234	[47]
	NA	NA	NA	5	NA	NA	100	80 000	292 000	[19]
	Portugal	2009	GS/2	2	SPE-LC-MS/MS	7/25	100	17 931	20 074	[6]
	Portugal	2011	TP/1	7	SPE-LC-MS/MS	NA	100	2463	9286	[7]
	Portugal	NA	TP/1	48	SPE-LC-MS	3/9	6	NA	342	[8]
	South Africa	2013	GS/3	3	SPE-LC-MS/MS	8.1/273	NA	6260	NA	[34]

**Pharmaceutical**	**Country**	**Year**	**Sampling Type/Locations**	**Number Of Samples**	**Method**	**LOD/LOQ (ng L^{-1})**	**Frequency (Highest Value) (%)**	**Average (Highest Value) (ng L^{-1})**	**Maximum (Highest Value) (ng L^{-1})**	**Reference**
	Spain	2003/2004	TP/1	10	SPE-LC-MS/MS	32/NA	100	134 000	246 000	[54]
	Spain	2008/2009	TP/3	42	SPE-LC-MS/MS	NA/110	100	44 800	201 000	[9]
	Spain	2009	TP/3	14	SPE-LC-MS/MS	NA/190	100	55 100	134 000	[9]
	Spain	NA	NA/1	12	SPE-LC-MS/MS	NA	NA	23 202	37 458	[30]
	Sweden	2013	FP/1	7	SPE-LC-MS/MS	NA	100	95 454	NA	[13]
	Taiwan	NA	GS/1	NA	SPE-LC-MS/MS	0.1	NA	2695	NA	[24]
	UK	2007	GS/2	20	SPE-LC-MS/MS	NA	100	194 748	482 687	[35]
	UK	NA	NA	NA	NA	NA	NA	NA	492 349	[43]
	USA	2010	TP/1	6	SPE-LC-MS/MS	2.6/8.7	NA	2800	NA	[37]
	USA	2010	FP/1	6	SPE-LC-MS/MS	2.5/7.5	100	18 000	150 000	[22]
							94.3(100)	44 048.4(194 748)	131 911.3(500 000)	
Total							86.6(100)	11 192.4(194 748)	49 449.3(700 000)	
Hormones										
Estrone (E1)	Canada	2004	TP/8	8	SPE-GC-MS/MS	1/NA	100	16	52	[44]
	Germany	2002	NA/1	18	SPE-LC-MS/MS	NA/1	100	188	NA	[65]
	International	NA	7	70	NA	NA	NA	53.9	132	[66]
	International	NA	NA	NA	NA	NA	NA	NA	170	[33]
	International	NA	NA	NA	NA	NA	NA	80	900	[11]

Pharmaceutical	Country	Year	Sampling Type/Locations	Number Of Samples	Method	LOD/LOQ (ng L^{-1})	Frequency (Highest Value) (%)	Average (Highest Value) (ng L^{-1})	Maximum (Highest Value) (ng L^{-1})	Reference
	Italy	2002	FP/1	7	SPE-LC-MS/MS	1.2/NA	100	35	60	[67]
	Japan	2001/2003	TP/5	12	SPE-GC-MS	NA	NA	NA	200	[52]
	Korea	2010	NA/5	5	SPE-LC-MS/MS	NA	100	47	70	[47]
	Luxembourg	2007/2008	TP/1	24	SPE-LC-MS/MS	0.3/1.0	NA	NA	9	[60]
	NA	NA	NA	109	NA	NA	100	67.2	670	[19]
	Portugal	NA	GS/5	10	SPE-LC-MS	18/60	20	NA	2484	[53]
	Portugal	NA	TP/1	48	SPE-LC-MS	18/60	40	NA	177	[8]
	UK	NA	NA	NA	NA	NA	NA	49	NA	[43]
	USA	2010	FP/1	6	SPE-LC-MS/MS	2.2/6.7	NA	64	350	[22]
							80.0(100)	66.7(188.0)	439.5(2484.0)	
17β-estradiol (E2)	Canada	2004	TP/8	8	SPE-GC-MS/MS	1/NA	100	9	22	[44]
	Canada	NA	TP/1	NA	SPE-LDTD-APCI-MS/MS	162/195	NA	310	NA	[51]
	Germany	2002	NA/1	18	SPE-LC-MS/MS	NA/2	100	11.8	NA	[65]
	International	NA	7	70	NA	NA	NA	12.0	25	[66]
	International	NA	NA	NA	NA	NA	NA	NA	50	[33]
	International	NA	NA	NA	NA	NA	NA	250	5000	[11]
	Italy	2002	FP/1	7	SPE-LC-MS/MS	1.9/NA	100	25	31	[67]
	Japan	2001/2003	TP/5	12	SPE-GC-MS	NA	NA	NA	30	[52]
	Korea	2010	NA/5	5	SPE-LC-MS/MS	NA	NA	4	4	[47]

Pharmaceutical	Country	Year	Sampling Type/Locations	Number Of Samples	Method	LOD/LOQ (ng L^{-1})	Frequency (Highest Value) (%)	Average (Highest Value) (ng L^{-1})	Maximum (Highest Value) (ng L^{-1})	Reference
	Luxembourg	2007/2008	TP/1	24	SPE-LC-MS/MS	1.0/3.0	NA	NA	102	[60]
	NA	NA	NA	108	NA	NA	100	22.2	12.5	[19]
	Portugal	NA	GS/5	10	SPE-LC-MS	4/12	10	NA	344	[53]
	Portugal	NA	TP/1	48	SPE-LC-MS	4/12	17	NA	937	[8]
	UK	NA	NA	NA	NA	NA	NA	20	NA	[43]
							71.2(100)	73.8(310.0)	596.1(5000.0)	
17α-estradiol (α-E2)	NA	NA	NA	36	NA	NA	100	7.4	17.2	[19]
	USA	2010	FP/1	6	SPE-LC-MS/MS	1.2/3.5	NA	<LOQ	10 000	[22]
							100(100)	3.7(7.4)	5008.6(10 000)	
17α-ethinylestradiol (EE2)	Canada	NA	TP/1	NA	SPE-LDTD-APCI-MS/MS	140/203	NA	388	NA	[51]
	Germany	2002	NA/1	18	SPE-LC-MS/MS	NA/2	100	8.8	NA	[65]
	International	NA	NA/7	70	NA	NA	NA	3.7	13	[66]
	International	NA	NA	NA	NA	NA	NA	NA	3	[33]
	International	NA	NA	NA	NA	NA	NA	20	80	[11]
	Luxembourg	2007/2008	TP/1	24	SPE-LC-MS/MS	2.0/6.0	NA	NA	24	[60]
	NA	NA	NA	70	NA	NA	91	4.2	70	[19]
	Portugal	NA	GS/5	10	SPE-LC-MS	21/69	20	NA	106	[53]
	Portugal	NA	TP/1	48	SPE-LC-MS	21/69	25	NA	80	[8]
	UK	NA	NA	NA	NA	NA	NA	1.0	NA	[43]
	USA	2010	FP/1	6	SPE-LC-MS/MS	1.3/3.8	NA	<LOQ	9.4	[22]
							59.0(100)	60.8(388.0)	48.2(106.0)	
Total							73.5(100)	62.9(388.0)	673.8(10 000)	

CS - Composite sampling
FP - Flow proportional sampling
FD - Fluorescence detection
GC - Gas chromatography

GS - Grab sampling
HPLC - High performance liquid chromatography
HRMS - High resolution mass spectrum
LC - Liquid chromatography
LDTD-APCI - Laser diode thermal desorption atmospheric pressure chemical ionization
LPME - Liquid-phase microextraction
LOQ - Limit of quantification
MS - Mass spectrometry
NA - Not available
ND - Not detected
SPE - Solid phase extraction
TP - Time proportional sampling
UPLC - Ultra performance liquid chromatography
UHPLC - Ultra high performance liquid chromatography

Table S3. Occurrence of pharmaceuticals in wastewater efluents (WWEs).

Pharmaceutical	Country	Year	Sampling Type/Locations	Number Of Samples	Method	LOD/LOQ (ng L^{-1})	Frequency (Highest Value) (%)	Average (Highest Value) (ng L^{-1})	Maximum (Highest Value) (ng L^{-1})	Reference
Anxiolytics										
Alprazolam (ALP)	Belgium	2013	TP/2	10	SPE-ESI-HRMS-LC	6.6/22	20	23	NA	[1]
	China	2011	GS/3	19	SPE-LC-MS/MS	NA/10	31	<LOQ	<LOQ	[3]
	China	2011	GS/3	3	SPE-LC-MS/MS	NA	0	ND	ND	[2]
	China	2014	TP/5	5	SPE-LC–MS/MS	NA/0.1	100	4.9	6	[4]
	EU	2010	GS/90	90	SPE-LC-MS/MS	NA/10	8	1.3	33.0	[68]
	India	2013	GS/2	7	SPE-LC-MS/MS	NA/NA	29	29	57	[5]
	Portugal	2009	GS/2	2	SPE-LC-MS/MS	1/4	0	ND	ND	[6]
	Portugal	2011	TP/1	7	SPE-LC-MS/MS	NA	100	27.5	33.5	[7]
	Spain	2008/2009	TP/3	42	SPE-LC-MS/MS	NA/10	38	<LOQ	<LOQ	[9]
	Spain	2009	TP/3	14	SPE-LC-MS/MS	NA/10	100	10	10	[9]
	USA	2011	TP/50	50	SPE-LC-MS/MS	9.1/NA	30	10	31	[69]
	USA	2013	TP/2	14	SPE-LC-MS/MS	NA	100	5	8	[10]
							46.3(100)	9.2(29.0)	16.2(57.0)	
Lorazepam (LOR)	China	2011	GS/3	19	SPE-LC-MS/MS	NA/70	50	<LOQ	<LOQ	[3]
	China	2011	GS/3	3	SPE-LC-MS/MS	NA	0	ND	ND	[2]

Pharmaceutical	Country	Year	Sampling Type/Locations	Number Of Samples	Method	LOD/LOQ (ng L^{-1})	Frequency (Highest Value) (%)	Average (Highest Value) (ng L^{-1})	Maximum (Highest Value) (ng L^{-1})	Reference
	China	2014	TP/5	5	SPE-LC–MS/MS	NA/4.1	100	NA	3.6	[4]
	India	2013	GS/2	7	SPE-LC-MS/MS	NA/NA	57	17.5	47	[5]
	International	NA	NA	NA	NA	NA	NA	200	200	[11]
	Italy	2010	CS/1	4	SPE-LC-MS/MS	NA	NA	105	NA	[70]
	Italy	2011	TP/2	6	SPE-LC-MS/MS	NA/17	84	61	82	[71]
	Portugal	2009	GS/2	2	SPE-LC-MS/MS	2/6	100	339	438	[6]
	Portugal	2011	TP/1	7	SPE-LC-MS/MS	NA	100	294	347	[7]
	Serbia	NA	GS/1	2	SPE-LC-MS/MS	0.55/1.82	0	ND	ND	[72]
	Spain	2007/2009	CS/3	24	SPE-LC-MS/MS	1.2/NA	NA	48	NA	[12]
	Spain	2008/2009	TP/3	42	SPE-LC-MS/MS	NA/30	55	40	60	[9]
	Spain	2009	TP/3	14	SPE-LC-MS/MS	NA/50	100	140	160	[9]
	USA	2013	TP/2	14	SPE-LC-MS/MS	NA	100	71	114	[10]
							67.8(100)	101.2(339.0)	121.0(438.0)	
Zolpidem (ZOL)	EU	2010	GS/90	90	SPE-LC-MS/MS	NA/0.5	58	1.5	42.6	[68]
	Portugal	2009	GS/2	2	SPE-LC-MS/MS	1/2	0	ND	ND	[6]
	Sweden	2013	FP/1	7	SPE-LC-MS/MS	NA	86	5	NA	[13]
							48.0(86.0)	2.2(5.0)	21.3(42.6)	
Total							55.6(100)	51.2(339.0)	66.9(438)	

Pharmaceutical	Country	Year	Sampling Type/Locations	Number Of Samples	Method	LOD/LOQ (ng L^{-1})	Frequency (Highest Value) (%)	Average (Highest Value) (ng L^{-1})	Maximum (Highest Value) (ng L^{-1})	Reference
Antibiotics										
Azithromycin (AZI)	Croatia	NA	NA/5	5	SPE-LC-MS/MS	3/10	100	96	210	[14]
	Czech Republic	2011/2012	TP/1	136	SPE-LC-MS/MS	NA/7	75	50	220	[15]
	Greece	2011	FP/1	8	SPE-LC-MS/MS	19/58.5	100	171	245	[16]
	International	NA	NA	NA	NA	NA	NA	160	7000	[11]
	Italy	2010	CS/1	4	SPE-LC-MS/MS	NA	NA	19	NA	[70]
	Italy	2011	TP/2	6	SPE-LC-MS/MS	NA/17	100	110	209	[71]
	Italy	NA	TP/1	4	SPE-LC-MS/MS	4/NA	NA	130	NA	[17]
	Japan	2007/2009	NA/9	9	NA	NA	NA	520	NA	[18]
	NA	NA	NA	6	NA	NA	100	138	NA	[19]
	Portugal	2009	GS/2	2	SPE-LC-MS/MS	2/5	100	683	836	[6]
	Portugal	2011	TP/1	7	SPE-LC-MS/MS	NA	100	171	297	[7]
	Portugal	2013/2014	FP/2	18	SPE-UHPLC-MS/MS	0.16/0.50	66.7	NA	21.5	[20]
	Serbia	NA	GS/1	2	SPE-LC-MS/MS	2.58/8.59	50	150	150	[72]
	Spain	2008/2009	TP/5	33	SPE-LC-MS/MS	7.1/23.5	22	440	1986	[73]
	Spain	2011	GS/3	3	SPE-UPLC-MS/MS	4.6/15.5	100	393	592	[21]
	USA	2010	FP/1	6	SPE-LC-MS/MS	3.7/11	NA	110	350	[22]

Pharmaceutical	Country	Year	Sampling Type/Locations	Number Of Samples	Method	LOD/LOQ (ng L^{-1})	Frequency (Highest Value) (%)	Average (Highest Value) (ng L^{-1})	Maximum (Highest Value) (ng L^{-1})	Reference
	USA	2014/2015	GS/2	6	SPE-UPLC-MS/MS	NA	NA	1465.2	5814.8	[74]
							83.1(100)	300.4(1465.2)	1379.3(7000.0)	
Clarithromycin (CLA)	Czech Republic	2011/2012	TP/1	136	SPE-LC-MS/MS	NA/3	100	930	2310	[15]
	Greece	2011	FP/1	8	SPE-LC-MS/MS	1.9/5.8	100	1153	1476	[16]
	International	NA	NA	NA	NA	NA	NA	290	800	[11]
	Italy	2007/2008	TP/4	24	SPE-LC-MS/MS	NA	NA	310	NA	[23]
	Italy	2010	CS/1	4	SPE-LC-MS/MS	NA	NA	265	NA	[70]
	Italy	2011	TP/2	6	SPE-LC-MS/MS	NA/13	100	193	374	[71]
	Italy	NA	TP/1	4	SPE-LC-MS/MS	2/NA	NA	280	NA	[17]
	Japan	2007/2009	NA/9	9	NA	NA	NA	593	NA	[18]
	NA	NA	NA	6	NA	NA	100	359	NA	[19]
	Portugal	2011	TP/1	7	SPE-LC-MS/MS	NA	100	22.4	40.0	[7]
	Portugal	2013/2014	FP/2	18	SPE-UHPLC-MS/MS	0.08/0.22	44.4	18.11	70.4	[20]
	Spain	2007/2009	CS/3	24	SPE-LC-MS/MS	1.2/NA	NA	38	NA	[12]
	Spain	2008/2009	TP/5	33	SPE-LC-MS/MS	2.9/9.7	25	202	589	[73]
	Spain	2009	TP/3	14	SPE-LC-MS/MS	NA/3	100	20	60	[9]
	Spain	2011	GS/3	3	SPE-UPLC-MS/MS	9.1/30.4	100	198	229	[21]

Pharmaceutical	Country	Year	Sampling Type/Locations	Number Of Samples	Method	LOD/LOQ (ng L^{-1})	Frequency (Highest Value) (%)	Average (Highest Value) (ng L^{-1})	Maximum (Highest Value) (ng L^{-1})	Reference
	Sweden	2013	FP/1	7	SPE-LC-MS/MS	NA	100	35	NA	[13]
	Taiwan	NA	GS/1	NA	SPE-LC-MS/MS	0.5/NA	NA	35.5	NA	[24]
	USA	2010	FP/1	6	SPE-LC-MS/MS	3.2/9.6	NA	<LOQ	19	[22]
							86.9(100)	274.6(1153.0)	596.7(2310.0)	
Ciprofloxacin (CIP)	Asia	NA	NA	NA	NA	NA	NA	NA	2050	[75]
	Belgium	2013	TP/2	10	SPE-ESI-HRMS-LC	31/102	40	106	ND	[1]
	Czech Republic	2011/2012	TP/1	136	SPE-LC-MS/MS	NA/3	91	65	190	[15]
	EU	2010	GS/90	90	SPE-LC-MS/MS	NA/15	90	96.3	264	[68]
	Europe	NA	NA	NA	NA	NA	NA	NA	499	[75]
	France	2014/2015	GS/6	24	SPE-UHPLC-MS/MS	24.5/28.2	NA	NA	NA	[76]
	Greece	2011	FP/1	8	SPE-LC-MS/MS	5.4/16.3	100	1116	1437	[16]
	Greece	2013/2014	TP/1	24	SPE-LC-MS/MS	14.7/48.6	41.7	199	591	[25]
	India (industry)	2008	GS/1	NA	SPE-LC-MS	NA/10	NA	NA	14 000 000	[77]
	International	NA	NA	NA	NA	NA	NA	860	9000	[11]
	Italy	2007/2008	TP/4	24	SPE-LC-MS/MS	NA	NA	14.5	NA	[23]
	Italy	2010	CS/1	4	SPE-LC-MS/MS	NA	NA	208	NA	[70]
	Italy	2011	TP/2	6	SPE-LC-MS/MS	NA/7	100	155	499	[71]

Pharmaceutical	Country	Year	Sampling Type/Locations	Number Of Samples	Method	LOD/LOQ (ng L^{-1})	Frequency (Highest Value) (%)	Average (Highest Value) (ng L^{-1})	Maximum (Highest Value) (ng L^{-1})	Reference
	Italy	NA	TP/1	4	SPE-LC-MS/MS	2/NA	NA	630	NA	[17]
	Kenya	2012/2013	GS/3	3	SPE-LC-MS	NA/NA	0	0	0	[26]
	Korea	2007	GS/10	10	SPE-LC-MS/MS	NA	0	ND	ND	[27]
	Korea	2008	GS/12	30	SPE-LC-MS/MS	NA	9	728	2050	[28]
	NA	NA	NA	29	NA	NA	91	72	140	[19]
	Portugal	2007	TP/1	2	SPE-LC-FD	17/NA	100	156.2	100.8	[29]
	Portugal	2009	GS/2	2	SPE-LC-MS/MS	1/4	0	ND	ND	[6]
	Portugal	2011	TP/1	7	SPE-LC-MS/MS	NA	100	369	1396	[7]
	Portugal	2013/2014	FP/2	18	SPE-UHPLC-MS/MS	20.2/61.2	27.8	73	179	[20]
	Spain	2008/2009	TP/5	33	SPE-LC-MS/MS	3.1/10.3	94	911	5692	[73]
	Spain	2009	TP/3	14	SPE-LC-MS/MS	NA/50	100	700	1080	[9]
	Spain	2011	GS/3	3	SPE-UPLC-MS/MS	5.6/18.5	66	101	147	[21]
	Spain	2016/2017	GS/3	45	SPE-LC-MS/MS	NA/1.8	93.3	321	4719	[78]
	Spain	NA	NA/1	12	SPE-LC-MS/MS	NA	NA	2378	5692	[30]
	Sweden	2002/2003	FP/5	10	SPE-LC-MS	NA/6	90	20	60	[31]
	Sweden	2013	FP/1	7	SPE-LC-MS/MS	NA	100	61	NA	[13]

Pharmaceutical	Country	Year	Sampling Type/Locations	Number Of Samples	Method	LOD/LOQ (ng L^{-1})	Frequency (Highest Value) (%)	Average (Highest Value) (ng L^{-1})	Maximum (Highest Value) (ng L^{-1})	Reference
	Taiwan	NA	GS/1	NA	SPE-LC-MS/MS	0.5/NA	0	ND	ND	[24]
	USA	2010	FP/1	6	SPE-LC-MS/MS	3.3/6.7	0	ND	ND	[22]
	USA	2011	TP/50	50	SPE-LC-MS/MS	10/NA	61	67	260	[69]
	USA	NA	CS/7	10	SPE-LC-MS	NA/50	40	60	140	[32]
							59.8(100)	326.4(2378.0)	501 292.4(14 000 000)	
Erythromycin (ERY)	Croatia	NA	NA/5	5	SPE-LC-MS/MS	6/20	NA	ND	ND	[14]
	Czech Republic	2011/2012	TP/1	136	SPE-LC-MS/MS	NA/6	100	77	300	[15]
	Greece	2011	FP/1	8	SPE-LC-MS/MS	155/470	0	ND	ND	[16]
	Greece	2013/2014	TP/1	24	SPE-LC-MS/MS	24.3/80.2	0	ND	ND	[25]
	International	NA	NA	NA	NA	NA	NA	NA	2840	[33]
	International	NA	NA	NA	NA	NA	NA	730	9000	[11]
	Italy	2007/2008	TP/4	24	SPE-LC-MS/MS	NA	NA	32.4	NA	[23]
	Italy	2010	CS/1	4	SPE-LC-MS/MS	NA	0	ND	ND	[70]
	Italy	NA	TP/1	4	SPE-LC-MS/MS	8/NA	NA	15	NA	[17]
	Japan	2007/2009	NA/9	9	NA	NA	NA	208	NA	[18]
	Korea	2004/2005	GS/7	7	SPE-LC-MS/MS	NA	71	130	294	[79]
	Korea	2007	GS/10	10	SPE-LC-MS/MS	NA	NA	NA	300	[27]

Pharmaceutical	Country	Year	Sampling Type/Locations	Number Of Samples	Method	LOD/LOQ (ng L^{-1})	Frequency (Highest Value) (%)	Average (Highest Value) (ng L^{-1})	Maximum (Highest Value) (ng L^{-1})	Reference
	Korea	2008	GS/12	30	SPE-LC-MS/MS	NA	3	146	146	[28]
	NA	NA	NA	3	NA	NA	100	212	290	[19]
	Portugal	2011	TP/1	7	SPE-LC-MS/MS	NA	100	71.2	134	[7]
	Scotland	2005/2006	GS/2	40	SPE-LC-MS/MS	0.1/0.5	55	381	2550	[80]
	Serbia	NA	GS/1	2	SPE-LC-MS/MS	1.65/5.48	0	ND	ND	[72]
	South Africa	2013	GS/3	3	SPE-LC-MS/MS	3/12	NA	240	NA	[34]
	Spain	2008/2009	TP/5	33	SPE-LC-MS/MS	29.7/98.9	83	679	6316	[73]
	Spain	2009	TP/3	14	SPE-LC-MS/MS	NA/8	100	80	120	[9]
	Spain	NA	NA/1	12	SPE-LC-MS/MS	NA	NA	331	760	[30]
	Sweden	2002/2003	FP/5	10	SPE-LC-MS	NA/160	0	ND	ND	[31]
	Taiwan	NA	GS/1	NA	SPE-LC-MS/MS	0.1/NA	NA	43.9	NA	[24]
	UK	2007	GS/2	20	SPE-LC-MS/MS	NA	100	1041	2841	[35]
	UK	NA	GS/1	3	SPE-LC-MS/MS	10/NA	100	163	180	[81]
	UK	NA	NA	NA	NA	NA	NA	NA	1385	[36]
	USA	2010	TP/1	6	SPE-LC-MS/MS	86.5/288.3	0	ND	ND	[37]
	USA	2010	FP/1	6	SPE-LC-MS/MS	2.7/8.2	100	180	340	[22]
	USA	2014/2015	GS/2	6	SPE-UPLC-MS/MS	NA	NA	7.8	15.49	[74]

Pharmaceutical	Country	Year	Sampling Type/Locations	Number Of Samples	Method	LOD/LOQ (ng L^{-1})	Frequency (Highest Value) (%)	Average (Highest Value) (ng L^{-1})	Maximum (Highest Value) (ng L^{-1})	Reference
	USA	NA	CS/7	10	SPE-LC-MS	NA/50	30	270	300	[32]
							52.3(100)	186.6(1041.0)	1124.5(9000.0)	
Total							66.0(100)	269.5(2378.0)	185 371.0(14 000 000)	
Lipid regulators										
Bezafibrate (BEZ)	Brazil	1997	TP/1	6	SPE-GC-MS	NA	NA	600	1100	[38]
	China	2009/2010	GS/2	24	SPE-UPLC-MS/MS	NA	91	16.1	51.4	[39]
	Croatia	NA	NA/5	5	SPE-LC-MS/MS	2/6	NA	NA	10	[14]
	EU	2010	GS/90	90	SPE-LC-MS/MS	NA/1	60	25.4	343	[68]
	France	2014/2015	GS/6	24	SPE-UHPLC-MS/MS	31/38.4	NA	25.05	57.6	[76]
	Greece	2010/2011	TP/8	32	SPE-LC-MS/MS	NA	47	39	344	[40]
	Greece	2013/2014	TP/1	24	SPE-LC-MS/MS	5.3/17.4	8.3	<LOD	<LOD	[25]
	International	NA	NA	NA	NA	NA	NA	NA	670	[33]
	International	NA	NA	NA	NA	NA	NA	900	8000	[11]
	Italy	2010	CS/1	4	SPE-LC-MS/MS	NA	NA	126	NA	[70]
	Italy	2011	TP/2	6	SPE-LC-MS/MS	NA/3	17	3	3	[71]
	Mexico	2015/2016	TP/2	8	SPE- LC-MS/MS	0.2/0.8	100	529	950	[41]
	NA	NA	NA	21	NA	NA	78	816	4800	[19]
	Portugal	2009	GS/2	2	SPE-LC-MS/MS	5/17	100	1070	1797	[6]

Pharmaceutical	Country	Year	Sampling Type/Locations	Number Of Samples	Method	LOD/LOQ (ng L^{-1})	Frequency (Highest Value) (%)	Average (Highest Value) (ng L^{-1})	Maximum (Highest Value) (ng L^{-1})	Reference
	Portugal	2011	TP/1	7	SPE-LC-MS/MS	NA	100	409	635	[7]
	Spain	2007	TP/1	5	SPE-LC-MS/MS	NA/3	100	97	130	[82]
	Spain	2007/2009	CS/3	24	SPE-LC-MS/MS	0.1/NA	NA	190	NA	[12]
	Spain	2008/2009	TP/5	33	SPE-LC-MS/MS	2.5/8.3	99	221	926	[73]
	Spain	2008/2009	TP/3	42	SPE-LC-MS/MS	NA/10	100	70	390	[9]
	Spain	2009	TP/3	14	SPE-LC-MS/MS	NA/10	100	60	80	[9]
	Spain	2012	GS./3	21	SPE-UPLC-MS/MS	NA/1.0	100	17	39.9	[42]
	Spain	2016/2017	GS/3	45	SPE-LC-MS/MS	NA/1.8	93.3	58	132	[78]
	Spain	NA	NA/1	12	SPE-LC-MS/MS	NA	NA	128	280	[30]
	UK	2007	GS/2	20	SPE-LC-MS/MS	NA	92	231	667	[35]
	UK	NA	NA	NA	NA	NA	NA	NA	418	[43]
							80.4(100)	255.9(1070.0)	948.9(8000.0)	
Gemfibrozil (GEM)	Brazil	1997	TP/1	6	SPE-GC-MS	NA	NA	200	1800	[38]
	Canada	2004	TP/8	8	SPE-GC-MS/MS	10/NA	100	190	2090	[44]
	Canada	2005	GS/2	4	SPE-GC-MS	0.3/NA	100	266.3	478.2	[83]
	China	2009/2010	GS/2	24	SPE-UPLC-MS/MS	NA	77	34.3	326	[39]
	China	2012/2013	GS/1	4	SPE-LC-MS/MS	0.1/NA	NA	20	NA	[45]

Pharmaceutical	Country	Year	Sampling Type/Locations	Number Of Samples	Method	LOD/LOQ (ng L^{-1})	Frequency (Highest Value) (%)	Average (Highest Value) (ng L^{-1})	Maximum (Highest Value) (ng L^{-1})	Reference
	China	2014	GS/3	12	SPE-LC-MS/MS	0.1/NA	75	1.0	2.1	[46]
	Croatia	NA	NA/5	5	SPE-LC-MS/MS	1/3	NA	120	320	[14]
	EU	2010	GS/90	90	SPE-LC-MS/MS	NA/1	60	138	3619	[68]
	Greece	2010/2011	TP/8	32	SPE-LC-MS/MS	NA	50	21	356	[40]
	Greece	2011	FP/1	8	SPE-LC-MS/MS	2.6/7.9	88	12.3	35	[16]
	Greece	2013/2014	TP/1	24	SPE-LC-MS/MS	35/105	0	ND	ND	[25]
	International	NA	NA	NA	NA	NA	NA	NA	5240	[33]
	International	NA	NA	NA	NA	NA	NA	930	7000	[11]
	Ireland	2011/2012	TP/2	12	SPE-LC-MS/MS	35/NA	83	365	650	[84]
	Italy	2010	CS/1	4	SPE-LC-MS/MS	NA	NA	84	NA	[70]
	Korea	2004/2005	GS/7	7	SPE-LC-MS/MS	NA	43	11.2	17	[79]
	Korea	2007	GS/10	10	SPE-LC-MS/MS	NA	0	ND	ND	[27]
	Korea	2010	NA/5	5	SPE-LC-MS/MS	NA	100	17	26	[47]
	Mexico	2015/2016	TP/2	8	SPE- LC-MS/MS	6.7/22	100	139	380	[41]
	NA	NA	NA	21	NA	NA	70	564	1340	[19]
	NA	NA	NA	NA	NA	NA	NA	NA	1500	[85]
	Portugal	2009	GS/2	2	SPE-LC-MS/MS	10/36	100	968	1183	[6]
	Portugal	2011	TP/1	7	SPE-LC-MS/MS	NA	NA	<LOQ	<LOQ	[7]

Pharmaceutical	Country	Year	Sampling Type/Locations	Number Of Samples	Method	LOD/LOQ (ng L^{-1})	Frequency (Highest Value) (%)	Average (Highest Value) (ng L^{-1})	Maximum (Highest Value) (ng L^{-1})	Reference
	Spain	2007	TP/1	5	SPE-LC-MS/MS	NA/4	100	2123	3090	[82]
	Spain	2007/2009	CS/3	24	SPE-LC-MS/MS	0.3/NA	NA	183	NA	[12]
	Spain	2008/2009	TP/5	33	SPE-LC-MS/MS	0.1/0.2	98	3903	36 364	[73]
	Spain	2008/2009	TP/3	42	SPE-LC-MS/MS	NA/20	100	540	1240	[9]
	Spain	2009	TP/3	14	SPE-LC-MS/MS	NA/4	100	490	910	[9]
	Spain	2012	GS/3	21	SPE-UPLC-MS/MS	NA/1.5	100	460	826.2	[42]
	Spain	NA	NA/1	12	SPE-LC-MS/MS	NA	NA	845	5233	[30]
	Sweden	2002	FP/1	1	SPE-GC/LC-MS/MS	NA	100	180	180	[48]
	USA	2010	FP/1	6	SPE-LC-MS/MS	1.6/4.8	100	170	1100	[22]
	USA	2011	TP/50	50	SPE-LC-MS/MS	10/NA	76	420	2300	[69]
	USA	2014/2015	GS/2	6	SPE-UPLC-MS/MS	NA	NA	755.63	1584.03	[74]
							74.8(100)	442.2(3903.0)	2554.5(36 364)	
Simvastatin (SIM)	Canada	2002	NA/1	3	SPE-LC-MS/MS	0.2/NA	NA	1	NA	[49]
	Greece	2010/2011	TP/8	32	SPE-LC-MS/MS	NA	9	<LOQ	<LOQ	[40]
	Greece	2011	FP/1	8	SPE-LC-MS/MS	28/84.2	25	ND	39.1	[16]
	Greece	2013/2014	TP/1	24	SPE-LC-MS/MS	9.7/32.1	37.5	205	1738	[25]

Pharmaceutical	Country	Year	Sampling Type/Locations	Number Of Samples	Method	LOD/LOQ (ng L-1)	Frequency (Highest Value) (%)	Average (Highest Value) (ng L-1)	Maximum (Highest Value) (ng L-1)	Reference
	International	NA	NA	NA	NA	NA	NA	2	2	[11]
	Malaysia	2013	GS/4	12	SPE-LC-MS/MS	NA/4	67	12	22	[50]
	Portugal	2009	GS/2	2	SPE-LC-MS/MS	10/35	100	726	1255	[6]
	Spain	2008/2009	TP/5	33	SPE-LC-MS/MS	1.8/6.1	0	ND	ND	[73]
	Spain	2009	TP/3	14	SPE-LC-MS/MS	NA/20	0	ND	ND	[9]
	UK	2007	GS/2	20	SPE-LC-MS/MS	NA	38	5	20	[35]
	UK	NA	NA	NA	NA	NA	NA	NA	5	[43]
	USA	2011	TP/50	50	SPE-LC-MS/MS	41/NA	24	<LOQ	<LOQ	[69]
							33.4(100)	86.5(726.0)	280.1(1738.0)	
Total							68.9(100)	319.0(3903.0)	1601.5(36 364)	
Antiepileptic										
Carbamazepine (CAR)	America	NA	NA	NA	NA	NA	NA	NA	1550	[75]
	Asia	NA	NA	NA	NA	NA	NA	NA	21 000	[75]
	Belgium	2013	TP/2	10	SPE-ESI-HRMS-LC	97/323	100	637	ND	[1]
	Canada	NA	TP/1	NA	SPE-LDTD-APCI-MS/MS	54/108	NA	274	NA	[51]
	China	2009/2010	GS/2	24	SPE-UPLC-MS/MS	NA	100	88.2	277	[39]
	China	2011	GS/3	16	SPE-LC-MS/MS	NA/0.5	100	NA	21.6	[3]
	China	2011	GS/3	3	SPE-LC-MS/MS	NA	100	17.3	22	[2]
	China	2012/2013	GS/1	4	SPE-LC-MS/MS	0.1/NA	NA	60	NA	[45]

Pharmaceutical	Country	Year	Sampling Type/Locations	Number Of Samples	Method	LOD/LOQ (ng L-1)	Frequency (Highest Value) (%)	Average (Highest Value) (ng L-1)	Maximum (Highest Value) (ng L-1)	Reference
	China	2014	GS/3	12	SPE-LC-MS/MS	0.1/NA	83	6.4	19.5	[46]
	China	2014	TP/5	5	SPE-LC–MS/MS	NA/1.6	100	34.5	47.3	[4]
	Croatia	NA	NA/5	5	SPE-LC-MS/MS	10/40	NA	410	630	[14]
	Czech Republic	2011/2012	TP/1	136	SPE-LC-MS/MS	NA/8	100	510	730	[15]
	EU	2010	GS/90	90	SPE-LC-MS/MS	NA/1	90	832	4609	[68]
	Europe	NA	NA	NA	NA	NA	NA	NA	150	[75]
	France	2014/2015	GS/6	24	SPE-UHPLC-MS/MS	0.03/0.1	NA	706.73	1007	[76]
	Greece	2010/2011	TP/8	32	SPE-LC-MS/MS	NA	94	126	417	[40]
	Greece	2011	FP/1	8	SPE-LC-MS/MS	21/64.2	100	461	501	[16]
	Greece	2013/2014	TP/1	24	SPE-LC-MS/MS	1.4/4.6	91.7	74.2	125	[25]
	India	2013	GS/2	7	SPE-LC-MS/MS	NA/NA	100	530	770	[5]
	International	NA	NA	NA	NA	NA	NA	NA	4600	[33]
	International	NA	NA	NA	NA	NA	NA	1040	13 000	[11]
	Ireland	2011/2012	TP/2	12	SPE-LC-MS/MS	15/NA	100	1567	3160	[84]
	Italy	2010	CS/1	4	SPE-LC-MS/MS	NA	NA	387	NA	[70]
	Italy	2011	TP/2	6	SPE-LC-MS/MS	NA/10	100	183	265	[71]
	Italy	NA	TP/1	4	SPE-LC-MS/MS	5/NA	NA	370	NA	[17]

Pharmaceutical	Country	Year	Sampling Type/Locations	Number Of Samples	Method	LOD/LOQ (ng L^{-1})	Frequency (Highest Value) (%)	Average (Highest Value) (ng L^{-1})	Maximum (Highest Value) (ng L^{-1})	Reference
	Japan	2001/2003	TP/5	12	SPE-GC-MS	NA	NA	NA	1200	[52]
	Kenya	2012/2013	GS/3	3	SPE-LC-MS	NA/NA	33.3	106.6	320	[26]
	Korea	2004/2005	GS/7	7	SPE-LC-MS/MS	NA	86	226	729	[79]
	Korea	2007	GS/10	10	SPE-LC-MS/MS	NA	NA	NA	250	[27]
	Korea	2008	GS/12	30	SPE-LC-MS/MS	NA	100	1750	21 000	[28]
	Korea	2010	NA/5	5	SPE-LC-MS/MS	NA	100	55	74	[47]
	Malaysia	2013	GS/4	12	SPE-LC-MS/MS	NA/4	83.3	27	53	[50]
	Mexico	2015/2016	TP/2	8	SPE- LC-MS/MS	0.7/2.3	100	284.5	476	[41]
	NA	NA	NA	63	NA	NA	100	674	2300	[19]
	Portugal	2011	TP/1	7	SPE-LC-MS/MS	NA	100	460	196	[7]
	Portugal	2013/2014	FP/2	18	SPE-UHPLC-MS/MS	0.12/0.34	100	117	245	[20]
	Portugal	NA	GS/5	9	SPE-LC-MS	2/7	10	NA	238	[53]
	Serbia	NA	GS/1	2	SPE-LC-MS/MS	0.27/0.9	100	14	15	[72]
	South Africa	2013	GS/3	3	SPE-LC-MS/MS	268/891	NA	1460	NA	[34]
	Spain	2003/2004	TP/1	10	SPE-LC-MS/MS	30/NA	100	130	230	[54]
	Spain	2007/2009	CS/3	24	SPE-LC-MS/MS	0.6/NA	NA	472	NA	[12]

Pharmaceutical	Country	Year	Sampling Type/Locations	Number Of Samples	Method	LOD/LOQ (ng L^{-1})	Frequency (Highest Value) (%)	Average (Highest Value) (ng L^{-1})	Maximum (Highest Value) (ng L^{-1})	Reference
	Spain	2008/2009	TP/5	33	SPE-LC-MS/MS	0.5/1.2	99	176	1593	[73]
	Spain	2016/2017	GS/3	45	SPE-LC-MS/MS	NA/1.8	100	64	390	[78]
	Spain	NA	NA/1	12	SPE-LC-MS/MS	NA	NA	117	173	[30]
	Sweden	2002	FP/1	1	SPE-GC/LC-MS/MS	NA	100	1180	1180	[48]
	Sweden	2007/2008	GS/1	10	SPE-LC-MS/MS	NA	100	437	NA	[86]
	Sweden	2013	FP/1	7	SPE-LC-MS/MS	NA	100	305	NA	[13]
	Taiwan	NA	GS/1	NA	SPE-LC-MS/MS	0.1	NA	0.9	NA	[24]
	UK	2007	GS/2	20	SPE-LC-MS/MS	NA	100	1663	4596	[35]
	UK	NA	NA	NA	NA	NA	NA	NA	3117	[43]
	USA	2010	TP/1	6	SPE-LC-MS/MS	2.2/7.3	NA	7	NA	[37]
	USA	2010	FP/1	6	SPE-LC-MS/MS	2.7/8.2	100	180	340	[22]
	USA	2011	TP/50	50	SPE-LC-MS/MS	4.4/NA	96	97	240	[69]
	USA	2012/2013	GS/4	28	SPE-UPLC-MS/MS	0.03/NA	71	0.61	2.04	[87]
	USA	2013	TP/2	14	SPE-LC-MS/MS	NA	100	289	731	[10]
	USA	2014/2015	GS/2	6	SPE-UPLC-MS/MS	NA	NA	118.83	158.35	[74]
							92.7(100)	398.4(1750.0)	2016.3(21 000)	
Selective serotonin reuptake inhibitors										

Pharmaceutical	Country	Year	Sampling Type/Locations	Number Of Samples	Method	LOD/LOQ (ng L^{-1})	Frequency (Highest Value) (%)	Average (Highest Value) (ng L^{-1})	Maximum (Highest Value) (ng L^{-1})	Reference
(SSRIs)										
Citalopram (CIT)	Canada	2007	FP/1	6	SPE-LC-MS/MS	0.077/NA	100	52.3	57.8	[55]
	China	2011	GS/3	19	SPE-LC-MS/MS	NA/0.2	100	NA	5.0	[3]
	China	2011	GS/3	3	SPE-LC-MS/MS	NA	100	11	5	[2]
	Czech Republic	2011/2012	TP/1	136	SPE-LC-MS/MS	NA/5	100	73	120	[15]
	EU	2010	GS/90	90	SPE-LC-MS/MS	NA/5	83	33.8	189	[68]
	India (industry)	2008	GS/1	NA	SPE-LC-MS	NA/10	NA	NA	430 000	[77]
	Norway	2005	FP/3	3	SPE-LC-MS/MS	NA/0.16	100	151	382	[56]
	Norway	2007	FP/2	8	LPME-LC-MS/MS	0.017/0.057	100	75	238.4	[57]
	Portugal	2011	TP/1	7	SPE-LC-MS/MS	NA	100	34.0	49.1	[7]
	Portugal	2013/2014	FP/2	18	SPE-UHPLC-MS/MS	0.70/2.12	44.4	25.05	67.4	[20]
	Spain	2008/2009	TP/5	33	SPE-LC-MS/MS	1.3/4.4	23	108	228	[73]
	Sweden	2013	FP/1	7	SPE-LC-MS/MS	NA	100	268	NA	[13]
	USA	2013	TP/2	14	SPE-LC-MS/MS	NA	100	215	414	[10]
							87.5(100)	95.1(268.0)	35 979.6(430 000)	
Desmethylcitalopram	Norway	2007	FP/2	8	LPME-LC-MS/MS	0.229/0.762	100	114	300.5	[57]

Pharmaceutical	Country	Year	Sampling Type/Locations	Number Of Samples	Method	LOD/LOQ (ng L^{-1})	Frequency (Highest Value) (%)	Average (Highest Value) (ng L^{-1})	Maximum (Highest Value) (ng L^{-1})	Reference
(N-CIT)	USA	2013	TP/2	14	SPE-LC-MS/MS	NA	93	99	310	[10]
							96.5(100)	106.5(114.0)	305.3(310.0)	
Fluoxetine (FLU)	Belgium	2013	TP/2	10	SPE-ESI-HRMS-LC	11/37	0	ND	ND	[1]
	Canada	2007	FP/1	6	SPE-LC-MS/MS	0.050/NA	100	2.9	3.7	[55]
	China	2011	GS/3	19	SPE-LC-MS/MS	NA/10	100	<LOQ	<LOQ	[3]
	China	2011	GS/3	3	SPE-LC-MS/MS	NA	0	ND	ND	[2]
	China	2012/2013	GS/1	4	SPE-LC-MS/MS	0.1/NA	0	ND	ND	[45]
	China	2014	TP/5	5	SPE-LC–MS/MS	NA/0.2	100	1.4	2.2	[4]
	China	2014	GS/3	12	SPE-LC-MS/MS	0.1/NA	0	ND	ND	[46]
	Croatia	NA	NA/5	5	SPE-LC-MS/MS	20/70	NA	ND	ND	[14]
	England	NA	GS/7	81	SPE-LC-MS/MS	NA	57	16.2	44.9	[43]
	EU	2010	GS/90	90	SPE-LC-MS/MS	NA/5	2	2.1	21.5	[68]
	International	NA	NA	NA	NA	NA	NA	240	12 000	[11]
	Italy	2010	CS/1	4	SPE-LC-MS/MS	NA	NA	44	NA	[70]
	Korea	2004/2005	GS/7	7	SPE-LC-MS/MS	NA	14	1.7	1.7	[79]
	NA	NA	NA	NA	NA	NA	NA	NA	841	[85]
	Norway	2005	FP/3	3	SPE-LC-MS/MS	NA/0.12	67	1.3	1.3	[56]

Pharmaceutical	Country	Year	Sampling Type/Locations	Number Of Samples	Method	LOD/LOQ (ng L^{-1})	Frequency (Highest Value) (%)	Average (Highest Value) (ng L^{-1})	Maximum (Highest Value) (ng L^{-1})	Reference
	Norway	2007	FP/2	8	LPME-LC-MS/MS	0.150/0.490	100	3.6	8.4	[57]
	Portugal	2009	GS/2	2	SPE-LC-MS/MS	1/4	0	ND	ND	[6]
	Portugal	2011	TP/1	7	SPE-LC-MS/MS	NA	NA	<LOQ	<LOQ	[7]
	Portugal	2013/2014	FP/2	18	SPE-UHPLC-MS/MS	0.04/0.12	100	14.8	34	[20]
	Portugal	NA	GS/5	9	SPE-LC-MS	15/57	0	ND	ND	[53]
	Spain	2008/2009	TP/5	33	SPE-LC-MS/MS	0.9/2.9	48	178	1972	[73]
	Spain	NA	NA/1	12	SPE-LC-MS/MS	NA	NA	223	929	[30]
	Sweden	2013	FP/1	7	SPE-LC-MS/MS	NA	43	9	NA	[13]
	UK	NA	NA	NA	NA	NA	NA	NA	29	[36]
	USA	2010	FP/1	6	SPE-LC-MS/MS	3.5/10.5	NA	28	96	[22]
	USA	2011	TP/50	50	SPE-LC-MS/MS	2.8/NA	38	8.7	31	[69]
	USA	2014/2015	GS/2	6	SPE-UPLC-MS/MS	NA	NA	399.89	1187.40	[74]
							42.7(100)	47.0(399.9)	688.1(12 000)	
Norfluoxetine (Nor-FLU)	Canada	2007	FP/1	6	SPE-LC-MS/MS	0.087/NA	100	1.8	1.8	[55]
	England	NA	GS/7	81	SPE-LC-MS/MS	NA	36	5.8	20.2	[43]
	International	NA	NA	NA	NA	NA	NA	10	10	[11]
	Norway	2007	FP/2	8	LPME-LC-MS/MS	0.1600.540	75	1.9	46.2	[57]

Pharmaceutical	Country	Year	Sampling Type/Locations	Number Of Samples	Method	LOD/LOQ (ng L^{-1})	Frequency (Highest Value) (%)	Average (Highest Value) (ng L^{-1})	Maximum (Highest Value) (ng L^{-1})	Reference
	Portugal	2011	TP/1	7	SPE-LC-MS/MS	NA	NA	36.1	99.6	[7]
	Portugal	2013/2014	FP/2	18	SPE-UHPLC-MS/MS	0.34/1.04	0	0	0	[20]
	UK	NA	NA	NA	NA	NA	NA	NA	13	[36]
	USA	2011	TP/50	50	SPE-LC-MS/MS	7.2/NA	17	7.7	15	[69]
							45.6(100)	9.0(36.1)	25.7(99.6)	
Paroxetine (PAR)	Belgium	2013	TP/2	10	SPE-ESI-HRMS-LC	74/248	0	ND	ND	[1]
	Canada	2007	FP/1	6	SPE-LC-MS/MS	0.096/NA	100	4.8	5.2	[55]
	China	2011	GS/3	19	SPE-LC-MS/MS	NA/80	88	<LOQ	<LOQ	[3]
	China	2011	GS/3	3	SPE-LC-MS/MS	NA	0	ND	ND	[2]
	Croatia	NA	NA/5	5	SPE-LC-MS/MS	7/26	NA	ND	ND	[14]
	EU	2010	GS/90	90	SPE-LC-MS/MS	NA	0	ND	ND	[68]
	International	NA	NA	NA	NA	NA	NA	7	7	[11]
	Italy	2010	CS/1	4	SPE-LC-MS/MS	NA	0	ND	ND	[70]
	Norway	2005	FP/3	3	SPE-LC-MS/MS	NA/0.12	100	0.9	1.6	[56]
	Norway	2007	FP/2	8	LPME-LC-MS/MS	0.053/0.205	100	4.8	11.7	[57]
	Portugal	2009	GS/2	2	SPE-LC-MS/MS	1/3	100	150	240	[6]
	Portugal	2011	TP/1	7	SPE-LC-MS/MS	NA	0	ND	ND	[7]

Pharmaceutical	Country	Year	Sampling Type/Locations	Number Of Samples	Method	LOD/LOQ (ng L^{-1})	Frequency (Highest Value) (%)	Average (Highest Value) (ng L^{-1})	Maximum (Highest Value) (ng L^{-1})	Reference
	Portugal	2013/2014	FP/2	18	SPE-UHPLC-MS/MS	9.26/28.0	5.55	13.25	26.5	[20]
	Portugal	NA	GS/5	9	SPE-LC-MS	27/89	33	NA	3367	[53]
	Spain	2008/2009	TP/5	33	SPE-LC-MS/MS	4.3/14.5	2	206	381	[73]
	Spain	2008/2009	TP/3	42	SPE-LC-MS/MS	NA/40	0	ND	ND	[9]
	Spain	2009	TP/3	14	SPE-LC-MS/MS	NA/170	0	ND	ND	[9]
	USA	2011	TP/50	50	SPE-LC-MS/MS	9.1/NA	0	ND	ND	[69]
	USA	2014/2015	GS/2	6	SPE-UPLC-MS/MS	NA	NA	3.42	5.24	[74]
							33.0(100)	21.7(206.0)	212.9(3367.0)	
Sertraline (SER)	Canada	2007	FP/1	6	SPE-LC-MS/MS	0.048/NA	100	5.5	5.8	[55]
	China	2011	GS/3	19	SPE-LC-MS/MS	NA/12	100	<LOQ	<LOQ	[3]
	China	2011	GS/3	3	SPE-LC-MS/MS	NA	0	ND	ND	[2]
	Czech Republic	2011/2012	TP/1	136	SPE-LC-MS/MS	NA/3	37	3	6	[15]
	EU	2010	GS/90	90	SPE-LC-MS/MS	NA/10	12	2.1	37.5	[68]
	Greece	2013/2014	TP/1	24	SPE-LC-MS/MS	1/3.3	45.8	<LOD	<LOD	[25]
	India	2013	GS/2	7	SPE-LC-MS/MS	NA/NA	78.5	8.85	21	[5]
	Norway	2005	FP/3	3	SPE-LC-MS/MS	NA/0.29	100	4.5	2.0	[56]

Pharmaceutical	Country	Year	Sampling Type/Locations	Number Of Samples	Method	LOD/LOQ (ng L^{-1})	Frequency (Highest Value) (%)	Average (Highest Value) (ng L^{-1})	Maximum (Highest Value) (ng L^{-1})	Reference
	Norway	2007	FP/2	8	LPME-LC-MS/MS	0.160/0.520	100	8.7	14.6	[57]
	Portugal	2011	TP/1	7	SPE-LC-MS/MS	NA	0	ND	ND	[7]
	Portugal	2013/2014	FP/2	18	SPE-UHPLC-MS/MS	0.04/0.14	55.6	NA	NA	[20]
	Sweden	2013	FP/1	7	SPE-LC-MS/MS	NA	100	28	NA	[13]
	USA	2011	TP/50	50	SPE-LC-MS/MS	5/NA	64	21	71	[69]
	USA	2013	TP/2	14	SPE-LC-MS/MS	NA	100	44	73	[10]
							63.8(100)	9.7(44.0)	19.2(73.0)	
Desmethylsertraline (Nor-SER)	Canada	2007	FP/1	6	SPE-LC-MS/MS	0.072/NA	100	4.2	4.7	[55]
	Norway	2007	FP/2	8	LPME-LC-MS/MS	0.618/4.1	63	8.8	10.6	[57]
	USA	2011	TP/50	50	SPE-LC-MS/MS	9.4/NA	18	9.9	24	[69]
	USA	2013	TP/2	14	SPE-LC-MS/MS	NA	93	64	83.6	[10]
							68.5(100)	21.7(64.0)	30.7(83.6)	
Total							55.4(100)	38.7(399.9)	5538.7(430 000)	
Anti-inflammatories										
Diclofenac (DIC)	America	NA	NA	NA	NA	NA	NA	NA	4200	[75]
	Asia	NA	NA	NA	NA	NA	NA	NA	1760	[75]
	Belgium	2013	TP/2	10	SPE-ESI-HRMS-LC	17/57	100	1051	ND	[1]
	Brazil	1997	TP/1	6	SPE-GC-MS	NA	NA	200	1500	[38]

Pharmaceutical	Country	Year	Sampling Type/Locations	Number Of Samples	Method	LOD/LOQ (ng L^{-1})	Frequency (Highest Value) (%)	Average (Highest Value) (ng L^{-1})	Maximum (Highest Value) (ng L^{-1})	Reference
	Canada	2004	TP/8	8	SPE-GC-MS/MS	10/NA	100	110	250	[44]
	Canada	2005	GS/2	4	SPE-GC-MS	1.0/NA	100	244.8	32	[83]
	Canada	NA	TP/1	NA	SPE-LDTD-APCI-MS/MS	70/196	NA	200	NA	[51]
	China	2009/2010	GS/2	24	SPE-UPLC-MS/MS	NA	100	185	463	[39]
	China	2012/2013	GS/1	4	SPE-LC-MS/MS	0.1/NA	NA	80	NA	[45]
	China	2014	GS/3	12	SPE-LC-MS/MS	0.1/NA	100	40	69.2	[46]
	Croatia	NA	NA/5	5	SPE-LC-MS/MS	10/30	NA	215	390	[14]
	EU	2010	GS/90	90	SPE-LC-MS/MS	NA/1	89	49.5	174	[68]
	Europe	NA	NA	NA	NA	NA	NA	NA	740	[75]
	France	2014/2015	GS/6	24	SPE-UHPLC-MS/MS	130/171	NA	1241	2476.3	[76]
	Greece	1998/1999	NA/4	11	SPE-GC-MS	1/2	0	ND	ND	[58]
	Greece	2009	FP/2	18	SPE-GC-MS	NA	NA	605	1070	[59]
	Greece	2010/2011	TP/8	32	SPE-LC-MS/MS	NA	72	97	383	[40]
	Greece	2011	FP/1	8	SPE-LC-MS/MS	21/63.6	100	874	987	[16]
	Greece	2013/2014	TP/1	24	SPE-LC-MS/MS	16.7/50.1	87.5	961	266.8	[25]
	International	NA	NA	NA	NA	NA	NA	NA	690	[33]

Pharmaceutical	Country	Year	Sampling Type/Locations	Number Of Samples	Method	LOD/LOQ (ng L^{-1})	Frequency (Highest Value) (%)	Average (Highest Value) (ng L^{-1})	Maximum (Highest Value) (ng L^{-1})	Reference
	Ireland	2011/2012	TP/2	12	SPE-LC-MS/MS	225/NA	100	880	1690	[84]
	Italy	2010	CS/1	4	SPE-LC-MS/MS	NA	NA	271	NA	[70]
	Italy	2011	TP/2	6	SPE-LC-MS/MS	NA/13	100	502	800	[71]
	Kenya	2012/2013	GS/3	3	SPE-LC-MS	NA/NA	100	40.3	61	[26]
	Korea	2004/2005	GS/7	7	SPE-LC-MS/MS	NA	100	40	127	[79]
	Korea	2007	GS/10	10	SPE-LC-MS/MS	NA	0	ND	ND	[27]
	Korea	2008	GS/12	30	SPE-LC-MS/MS	NA	65	191	1760	[28]
	Korea	2010	NA/5	5	SPE-LC-MS/MS	NA	100	24	49	[47]
	Luxembourg	2007/2008	TP/1	24	SPE-LC-MS/MS	0.3/1.0	NA	NA	78	[60]
	Malaysia	2013	GS/4	12	SPE-LC-MS/MS	NA/46	0	ND	ND	[50]
	Mexico	2015/2016	TP/2	8	SPE- LC-MS/MS	1.5/4.9	100	1311.5	2180	[41]
	NA	NA	NA	101	NA	NA	85	680	1950	[19]
	NA	NA	NA	NA	NA	NA	NA	NA	33 900	[85]
	Portugal	2009	GS/2	2	SPE-LC-MS/MS	17/79	100	1268	1429	[6]
	Portugal	2011	TP/1	7	SPE-LC-MS/MS	NA	100	42.9	83.1	[7]
	Portugal	2013/2014	FP/2	18	SPE-UHPLC-MS/MS	1.40/4.20	38.9	236	724	[20]

Pharmaceutical	Country	Year	Sampling Type/Locations	Number Of Samples	Method	LOD/LOQ (ng L^{-1})	Frequency (Highest Value) (%)	Average (Highest Value) (ng L^{-1})	Maximum (Highest Value) (ng L^{-1})	Reference
	Portugal	NA	GS/5	9	SPE-LC-MS	7/24	22	NA	1612	[53]
	Scotland	2005/2006	GS/2	40	SPE-LC-MS/MS	0.2/0.5	100	34	203	[80]
	Serbia	NA	GS/1	2	SPE-LC-MS/MS	0.15/0.49	0	ND	ND	[72]
	Spain	2003/2004	TP/1	10	SPE-LC-MS/MS	100/NA	NA	900	2200	[54]
	Spain	2007	TP/1	5	SPE-LC-MS/MS	NA/4	100	1293	1440	[82]
	Spain	2007/2009	CS/3	24	SPE-LC-MS/MS	1.2/NA	NA	526	NA	[12]
	Spain	2008/2009	TP/5	33	SPE-LC-MS/MS	0.4/1.2	21	3968	25 552	[73]
	Spain	2008/2009	TP/3	42	SPE-LC-MS/MS	NA/50	100	330	740	[9]
	Spain	2009	TP/3	14	SPE-LC-MS/MS	NA/50	100	340	620	[9]
	Spain	2012	GS/3	21	SPE-UPLC-MS/MS	NA/4.0	100	233	293.7	[42]
	Spain	2016/2017	GS/3	45	SPE-LC-MS/MS	NA/1.8	86.6	701	1932	[78]
	Spain	NA	NA/1	12	SPE-LC-MS/MS	NA	NA	220	431	[30]
	Spain/Belgium/Germany	NA	TP/NA	25	SPE-GC/LC-MS/MS	7/20	NA	NA	1420	[62]
	Sweden	2002	FP/1	1	SPE-GC/LC-MS/MS	NA	100	120	120	[48]
	Sweden	2013	FP/1	7	SPE-LC-MS/MS	NA	100	536	NA	[13]

Pharmaceutical	Country	Year	Sampling Type/Locations	Number Of Samples	Method	LOD/LOQ (ng L^{-1})	Frequency (Highest Value) (%)	Average (Highest Value) (ng L^{-1})	Maximum (Highest Value) (ng L^{-1})	Reference
	Switzerland	2003/2004	FP/5	44	SPE-GC-MS	6/20	100	1350	1900	[63]
	Taiwan	NA	GS/1	NA	SPE-LC-MS/MS	1	NA	12.6	NA	[24]
	UK	2007	GS/2	20	SPE-LC-MS/MS	NA	100	139	496	[35]
	UK	NA	GS/1	3	SPE-LC-MS/MS	20/NA	100	407	460	[81]
	UK	NA	NA	NA	NA	NA	NA	NA	599	[36]
							80.2(100)	484.0(3968.0)	2006.0(33 900)	
Ibuprofen (IBU)	America	NA	NA	NA	NA	NA	NA	NA	11 900	[75]
	Asia	NA	NA	NA	NA	NA	NA	NA	1600	[75]
	Belgium	2013	TP/2	10	SPE-ESI-HRMS-LC	140/466	0	ND	ND	[1]
	Brazil	1997	TP/1	6	SPE-GC-MS	NA	NA	100	3700	[38]
	Canada	2004	TP/8	8	SPE-GC-MS/MS	10/NA	100	310	2170	[44]
	Canada	2005	GS/2	4	SPE-GC-MS	0.8/NA	100	4766	67 118	[83]
	Canada	2010	GS/9	9	SPE-GC-MS/MS	7/NA	NA	NA	5200	[88]
	China	2012/2013	GS/1	4	SPE-LC-MS/MS	0.1/NA	NA	180	NA	[45]
	China	2014	GS/3	12	SPE-LC-MS/MS	0.1/NA	92	50	99.4	[46]
	Croatia	NA	NA/5	5	SPE-LC-MS/MS	12/20	100	266	800	[14]
	EU	2010	GS/90	90	SPE-LC-MS/MS	NA/2	57	80.5	2129	[68]
	Europe	NA	NA	NA	NA	NA	NA	NA	8000	[75]

Pharmaceutical	Country	Year	Sampling Type/Locations	Number Of Samples	Method	LOD/LOQ (ng L^{-1})	Frequency (Highest Value) (%)	Average (Highest Value) (ng L^{-1})	Maximum (Highest Value) (ng L^{-1})	Reference
	France	2014/2015	GS/6	24	SPE-UHPLC-MS/MS	4.8/9.8	NA	163.2	526.7	[76]
	Greece	1998/1999	NA/4	11	SPE-GC-MS	0.6/1.6	100	ND	365	[58]
	Greece	2009	FP/2	18	SPE-GC-MS	NA	0	ND	ND	[59]
	Greece	2010/2011	TP/8	32	SPE-LC-MS/MS	NA	25	<LOQ	301	[40]
	Greece	2011	FP/1	8	SPE-LC-MS/MS	16/47	0	ND	ND	[16]
	India	2013	GS/2	7	SPE-LC-MS/MS	NA/NA	100	805	1940	[5]
	International	NA	NA	NA	NA	NA	NA	NA	55 000	[33]
	Italy	2010	CS/1	4	SPE-LC-MS/MS	NA	NA	58	NA	[70]
	Japan	2001/2003	TP/5	12	SPE-GC-MS	NA	NA	NA	200	[52]
	Kenya	2012/2013	GS/3	3	SPE-LC-MS	NA/NA	66.6	1326.6	2070	[26]
	Korea	2004/2005	GS/7	7	SPE-LC-MS/MS	NA	71	65	137	[79]
	Korea	2007	GS/10	10	SPE-LC-MS/MS	NA	NA	NA	40	[27]
	Korea	2008	GS/12	30	SPE-LC-MS/MS	NA	0	ND	ND	[28]
	Korea	2010	NA/5	5	SPE-LC-MS/MS	NA	100	40	75	[47]
	Luxembourg	2007/2008	TP/1	24	SPE-LC-MS/MS	0.3/1.0	100	NA	359	[60]
	Mexico	2015/2016	TP/2	8	SPE- LC-MS/MS	2.3/7.6	0	0	0	[41]

Pharmaceutical	Country	Year	Sampling Type/Locations	Number Of Samples	Method	LOD/LOQ (ng L^{-1})	Frequency (Highest Value) (%)	Average (Highest Value) (ng L^{-1})	Maximum (Highest Value) (ng L^{-1})	Reference
	NA	NA	NA	109	NA	NA	93	1960	24 600	[19]
	NA	NA	NA	NA	NA	NA	NA	NA	850	[85]
	Portugal	2009	GS/2	2	SPE-LC-MS/MS	62/208	100	1308	1527	[6]
	Portugal	2011	TP/1	7	SPE-LC-MS/MS	NA	NA	119	369	[7]
	Portugal	2013/2014	FP/2	18	SPE-UHPLC-MS/MS	27.8/92.7	100	1406	3304	[20]
	Portugal	NA	GS/5	9	SPE-LC-MS	14/46	89	NA	43 653	[53]
	Scotland	2005/2006	GS/2	40	SPE-LC-MS/MS	1.1/3.7	98	278	2206	[80]
	Serbia	NA	GS/1	2	SPE-LC-MS/MS	3.28/10.93	0	ND	ND	[72]
	South Africa	2013	GS/3	3	SPE-LC-MS/MS	244/813	NA	12 940	NA	[34]
	Spain	2003/2004	TP/1	10	SPE-LC-MS/MS	23/NA	100	7100	28 000	[54]
	Spain	2007	TP/1	5	SPE-LC-MS/MS	NA/13	100	180	340	[82]
	Spain	2008/2009	TP/5	33	SPE-LC-MS/MS	1.1/3.7	13	618	2351	[73]
	Spain	2008/2009	TP/3	42	SPE-LC-MS/MS	NA/250	33	<LOQ	<LOQ	[9]
	Spain	2009	TP/3	14	SPE-LC-MS/MS	NA/150	0	ND	ND	[9]
	Spain	2011/2012	NA/1	5	SPE-LC-MS/MS	2.1/6.9	100	1112	1900	[64]
	Spain	2012	GS/3	21	SPE-UPLC-MS/MS	NA/20	0	ND	ND	[42]

Pharmaceutical	Country	Year	Sampling Type/Locations	Number Of Samples	Method	LOD/LOQ (ng L^{-1})	Frequency (Highest Value) (%)	Average (Highest Value) (ng L^{-1})	Maximum (Highest Value) (ng L^{-1})	Reference
	Spain	NA	NA/1	12	SPE-LC-MS/MS	NA	NA	135	653	[30]
	Spain/Belgium/Germany	NA	TP/NA	14	SPE-GC/LC-MS/MS	12/42	NA	NA	6900	[62]
	Spain/Belgium/Germany	NA	TP/NA	14	SPE-GC/LC-MS/MS	26/75	NA	NA	455	[62]
	Sweden	2002	FP/1	1	SPE-GC/LC-MS/MS	NA	100	150	150	[48]
	Sweden	2013	FP/1	7	SPE-LC-MS/MS	NA	0	ND	ND	[13]
	Switzerland	2003/2004	FP/4	44	SPE-GC-MS	8/30	100	1350	2500	[63]
	Taiwan	NA	GS/1	NA	SPE-LC-MS/MS	5	NA	228	NA	[24]
	UK	2007	GS/2	20	SPE-LC-MS/MS	NA	100	203	491	[35]
	UK	NA	GS/1	3	SPE-LC-MS/MS	50/NA	100	2433	3800	[81]
	UK	NA	NA	NA	NA	NA	NA	NA	4239	[36]
	USA	2010	FP/1	6	SPE-LC-MS/MS	4.7/14	0	ND	ND	[22]
	USA	2011	TP/50	50	SPE-LC-MS/MS	12/NA	46	460	4200	[69]
	USA	2012/2013	GS/4	28	SPE-UPLC-MS/MS	0.085/NA	68	927	10 600	[87]
	USA	2014/2015	GS/2	6	SPE-UPLC-MS/MS	NA	NA	286.06	536.72	[74]
							61.9(100)	920.1(12 940)	5691.8(67 118)	
Naproxen	America	NA	NA	NA	NA	NA	NA	NA	1550	[75]

Pharmaceutical	Country	Year	Sampling Type/Locations	Number Of Samples	Method	LOD/LOQ (ng L^-1^)	Frequency (Highest Value) (%)	Average (Highest Value) (ng L^-1^)	Maximum (Highest Value) (ng L^-1^)	Reference
(NAP)	Belgium	2013	TP/2	10	SPE-ESI-HRMS-LC	345/1149	0	ND	ND	[1]
	Brazil	1997	TP/1	6	SPE-GC-MS	NA	NA	100	3000	[38]
	Canada	2004	TP/8	8	SPE-GC-MS/MS	10/NA	100	820	2540	[44]
	Canada	2005	GS/2	4	SPE-GC-MS	0.5/NA	100	4184	7962	[83]
	Canada	2010	GS/9	9	SPE-GC-MS/MS	41/NA	NA	NA	3200	[88]
	China	2012/2013	GS/1	4	SPE-LC-MS/MS	0.1/NA	NA	5	NA	[45]
	China	2014	GS/3	12	SPE-LC-MS/MS	0.1/NA	42	4.5	13.8	[46]
	Croatia	NA	NA/5	5	SPE-LC-MS/MS	9/32	NA	108	160	[14]
	EU	2010	GS/90	90	SPE-LC-MS/MS	NA/5	66	26.7	958	[68]
	France	2014/2015	GS/6	24	SPE-UHPLC-MS/MS	12.7/19.2	NA	177.5	355.3	[76]
	Greece	2009	FP/2	18	SPE-GC-MS	NA	NA	45	110	[59]
	Greece	2010/2011	TP/8	32	SPE-LC-MS/MS	NA	50	158	1076	[40]
	Greece	2011	FP/1	8	SPE-LC-MS/MS	16/47	100	142	176	[16]
	Greece	2013/2014	TP/1	24	SPE-LC-MS/MS	18.1/54.3	0	ND	ND	[25]
	International	NA	NA	NA	NA	NA	NA	NA	5090	[33]
	Italy	2010	CS/1	4	SPE-LC-MS/MS	NA	NA	114	NA	[70]

Pharmaceutical	Country	Year	Sampling Type/Locations	Number Of Samples	Method	LOD/LOQ (ng L^{-1})	Frequency (Highest Value) (%)	Average (Highest Value) (ng L^{-1})	Maximum (Highest Value) (ng L^{-1})	Reference
	Italy	2011	TP/2	6	SPE-LC-MS/MS	NA/13	17	21	21	[71]
	Japan	2001/2003	TP/5	12	SPE-GC-MS	NA	NA	NA	200	[52]
	Korea	2004/2005	GS/7	7	SPE-LC-MS/MS	NA	100	128	483	[79]
	Korea	2007	GS/10	10	SPE-LC-MS/MS	NA	NA	NA	250	[27]
	Korea	2008	GS/12	30	SPE-LC-MS/MS	NA	26	250	740	[28]
	Korea	2010	NA/5	5	SPE-LC-MS/MS	NA	100	111	166	[47]
	Mexico	2015/2016	TP/2	8	SPE- LC-MS/MS	1.2/4.2	100	168.5	392	[41]
	NA	NA	NA	53	NA	NA	87	1890	33900	[19]
	NA	NA	NA	NA	NA	NA	NA	NA	520	[85]
	Portugal	2009	GS/2	2	SPE-LC-MS/MS	14/49	100	1575	2748	[6]
	Portugal	2011	TP/1	7	SPE-LC-MS/MS	NA	NA	303	774	[7]
	Portugal	2013/2014	FP/2	18	SPE-UHPLC-MS/MS	0.10/0.30	55.55	44	270	[20]
	Portugal	NA	GS/5	9	SPE-LC-MS	18/59	0	ND	ND	[53]
	Spain	2007	TP/1	5	SPE-LC-MS/MS	NA/18	100	717	1150	[82]
	Spain	2007/2009	CS/3	24	SPE-LC-MS/MS	0.9/NA	NA	562	NA	[12]
	Spain	2008/2009	TP/5	33	SPE-LC-MS/MS	7.2/24.1	65	1636	32241	[73]

Pharmaceutical	Country	Year	Sampling Type/Locations	Number Of Samples	Method	LOD/LOQ (ng L^{-1})	Frequency (Highest Value) (%)	Average (Highest Value) (ng L^{-1})	Maximum (Highest Value) (ng L^{-1})	Reference
	Spain	2008/2009	TP/3	42	SPE-LC-MS/MS	NA/30	100	170	720	[9]
	Spain	2009	TP/3	14	SPE-LC-MS/MS	NA/20	100	130	280	[9]
	Spain	2012	GS/3	21	SPE-UPLC-MS/MS	NA/2	100	102	306.6	[42]
	Spain	NA	NA/1	12	SPE-LC-MS/MS	NA	NA	923	2208	[30]
	Spain/Belgium/Germany	NA	TP/NA	14	SPE-GC/LC-MS/MS	26/75	NA	NA	625	[62]
	Sweden	2002	FP/1	1	SPE-GC/LC-MS/MS	NA	100	250	250	[48]
	Sweden	2013	FP/1	7	SPE-LC-MS/MS	NA	NA	785	NA	[13]
	Taiwan	NA	GS/1	NA	SPE-LC-MS/MS	5	NA	15.7	NA	[24]
	UK	2007	GS/2	20	SPE-LC-MS/MS	NA	96	270	703	[35]
	UK	NA	NA	NA	NA	NA	NA	NA	370	[36]
	USA	2010	FP/1	6	SPE-LC-MS/MS	1.0/2.9	100	140	580	[22]
	USA	2014/2015	GS/2	6	SPE-UPLC-MS/MS	NA	NA	166.08	346.18	[74]
							72.2(100)	439.0(4184.0)	2660.9(33 900)	
Paracetamol (PARA)	Belgium	2013	TP/2	10	SPE-ESI-HRMS-LC	60/200	0	ND	ND	[1]
	China	2012/2013	GS/1	4	SPE-LC-MS/MS	0.1/NA	NA	10	NA	[45]
	China	2014	GS/3	12	SPE-LC-MS/MS	0.1/NA	8.3	0.9	11.1	[46]

Pharmaceutical	Country	Year	Sampling Type/Locations	Number Of Samples	Method	LOD/LOQ (ng L^{-1})	Frequency (Highest Value) (%)	Average (Highest Value) (ng L^{-1})	Maximum (Highest Value) (ng L^{-1})	Reference
	Croatia	NA	NA/5	5	SPE-LC-MS/MS	10/58	NA	2102	5990	[14]
	France	2014/2015	GS/6	24	SPE-UHPLC-MS/MS	75.9/130.3	NA	NA	NA	[76]
	Greece	2010/2011	TP/8	32	SPE-LC-MS/MS	NA	78	154	1060	[40]
	Greece	2011	FP/1	8	SPE-LC-MS/MS	23/64.2	100	1926	7420	[16]
	Greece	2013/2014	TP/1	24	SPE-LC-MS/MS	10.1/33.3	8.3	<LOD	305	[25]
	International	NA	NA	NA	NA	NA	NA	NA	30	[33]
	Italy	2010	CS/1	4	SPE-LC-MS/MS	NA	NA	16	NA	[70]
	Kenya	2012/2013	GS/3	3	SPE-LC-MS	NA/NA	33.3	33.3	100	[26]
	Korea	2004/2005	GS/7	7	SPE-LC-MS/MS	NA	43	9.5	19	[79]
	Korea	2007	GS/10	10	SPE-LC-MS/MS	NA	0	ND	ND	[27]
	Korea	2008	GS/12	30	SPE-LC-MS/MS	NA	3	652	652	[28]
	Korea	2010	NA/5	5	SPE-LC-MS/MS	NA	NA	10	27	[47]
	NA	NA	NA	5	NA	NA	0	ND	ND	[19]
	Portugal	2009	GS/2	2	SPE-LC-MS/MS	6/22	0	ND	ND	[6]
	Portugal	2011	TP/1	7	SPE-LC-MS/MS	NA	100	96.1	106	[7]
	Scotland	2005/2006	GS/2	40	SPE-LC-MS/MS	10.9/36.7	75	762	22782	[80]

Pharmaceutical	Country	Year	Sampling Type/Locations	Number Of Samples	Method	LOD/LOQ (ng L^{-1})	Frequency (Highest Value) (%)	Average (Highest Value) (ng L^{-1})	Maximum (Highest Value) (ng L^{-1})	Reference
	Serbia	NA	GS/1	2	SPE-LC-MS/MS	0.50/1.67	0	ND	ND	[72]
	South Africa	2013	GS/3	3	SPE-LC-MS/MS	8.1/273	NA	3270	NA	[34]
	Spain	2003/2004	TP/1	10	SPE-LC-MS/MS	32/NA	NA	220	4300	[54]
	Spain	2008/2009	TP/5	33	SPE-LC-MS/MS	9.8/32.7	98	103	2308	[73]
	Spain	2008/2009	TP/3	42	SPE-LC-MS/MS	NA/90	0	ND	ND	[9]
	Spain	2009	TP/3	14	SPE-LC-MS/MS	NA/90	0	ND	ND	[9]
	Spain	NA	NA/1	12	SPE-LC-MS/MS	NA	0	ND	ND	[30]
	Sweden	2013	FP/1	7	SPE-LC-MS/MS	NA	14	89	NA	[13]
	Taiwan	NA	GS/1	NA	SPE-LC-MS/MS	0.1	NA	6.9	NA	[24]
	UK	2007	GS/2	20	SPE-LC-MS/MS	NA	93	309	1575	[35]
	UK	NA	NA	NA	NA	NA	NA	NA	11733	[43]
	USA	2010	TP/1	6	SPE-LC-MS/MS	2.6/8.7	NA	6	NA	[37]
	USA	2010	FP/1	6	SPE-LC-MS/MS	2.5/7.5	NA	39	650	[22]
	USA	2011	TP/50	50	SPE-LC-MS/MS	5/NA	14	79	1560	[69]
	USA	2014/2015	GS/2	6	SPE-UPLC-MS/MS	NA	NA	9.5	22.2	[74]
							31.8(100)	319.5(3270.0)	2246.3(22 782)	
Total							64.4(100)	564.4(12 940)	3361.1(67 118)	
Hormones										

Pharmaceutical	Country	Year	Sampling Type/Locations	Number Of Samples	Method	LOD/LOQ (ng L^{-1})	Frequency (Highest Value) (%)	Average (Highest Value) (ng L^{-1})	Maximum (Highest Value) (ng L^{-1})	Reference
Estrone (E1)	Canada	2004	TP/8	8	SPE-GC-MS/MS	1/NA	87.5	5	54	[44]
	EU	2010	GS/90	90	SPE-LC-MS/MS	NA	0	ND	ND	[68]
	France	2014/2015	GS/6	24	SPE-UHPLC-MS/MS	0.4/1.2	NA	14.8	44.2	[76]
	Germany	2002	NA/1	18	SPE-LC-MS/MS	NA/0.2	100	12.6	NA	[65]
	International	NA	7	70	NA	NA	NA	16.4	82	[66]
	International	NA	NA	NA	NA	NA	NA	30	100	[11]
	Italy	2002	FP/1	7	SPE-LC-MS/MS	0.8/NA	100	16	30	[67]
	Japan	2001	GS/2	2	SPE-GC-MS/MS	0.1	100	18.3	34	[89]
	Japan	2001/2003	TP/5	12	SPE-GC-MS	NA	NA	NA	110	[52]
	Korea	2004/2005	GS/7	7	SPE-LC-MS/MS	NA	71	14	36	[79]
	Korea	2010	NA/5	5	SPE-LC-MS/MS	NA	NA	6	24	[47]
	Korea	NA	NA	NA	NA	NA	NA	NA	36	[75]
	Luxembourg	2007/2008	TP/1	24	SPE-LC-MS/MS	0.3/1.0	NA	NA	14	[60]
	NA	NA	NA	79	NA	NA	93	20.9	95	[19]
	Portugal	NA	GS/5	9	SPE-LC-MS	18/60	11	NA	35	[53]
	Sweden	NA	NA	NA	NA	NA	NA	NA	70	[75]
	UK	NA	NA	NA	NA	NA	NA	NA	12	[43]
	USA	2010	FP/1	6	SPE-LC-MS/MS	2.2/6.7	0	ND	ND	[22]
	USA	2012/2013	GS/4	28	SPE-UPLC-MS/MS	6.48/NA	4	NA	11.3	[87]

Pharmaceutical	Country	Year	Sampling Type/Locations	Number Of Samples	Method	LOD/LOQ (ng L^{-1})	Frequency (Highest Value) (%)	Average (Highest Value) (ng L^{-1})	Maximum (Highest Value) (ng L^{-1})	Reference
							56.7(100)	12.8(30.0)	43.8(110.0)	
17β-estradiol (E2)	Canada	2004	TP/8	8	SPE-GC-MS/MS	1/NA	50	2	2	[44]
	Canada	NA	TP/1	NA	SPE-LDTD-APCI-MS/MS	162/195	NA	290	NA	[51]
	EU	2010	GS/90	90	SPE-LC-MS/MS	NA	0	ND	ND	[68]
	France	2014/2015	GS/6	24	SPE-UHPLC-MS/MS	2.2/7.1	NA	NA	NA	[76]
	Germany	2002	NA/1	18	SPE-LC-MS/MS	NA/0.4	61	0.8	NA	[65]
	International	NA	7	70	NA	NA	NA	1.3	3.5	[66]
	International	NA	NA	NA	NA	NA	NA	10	80	[11]
	Italy	2002	FP/1	7	SPE-LC-MS/MS	0.8/NA	100	6	8	[67]
	Japan	2001	GS/2	2	SPE-GC-MS/MS	0.2	100	1.4	2.5	[89]
	Japan	2001/2003	TP/5	12	SPE-GC-MS	NA	NA	NA	20	[52]
	Korea	2010	NA/5	5	SPE-LC-MS/MS	NA	0	ND	ND	[47]
	Korea	NA	NA	NA	NA	NA	NA	NA	<1	[75]
	Luxembourg	2007/2008	TP/1	24	SPE-LC-MS/MS	1.0/3.0	NA	NA	85	[60]
	NA	NA	NA	63	NA	NA	74	2.8	30	[19]
	Portugal	NA	GS/5	9	SPE-LC-MS	4/12	0	ND	ND	[53]
	Sweden	NA	NA	NA	NA	NA	NA	NA	9.2	[75]
	UK	NA	NA	NA	NA	NA	NA	NA	1.4	[43]
	USA	2012/2013	GS/4	28	SPE-UPLC-MS/MS	6.48/NA	4	NA	38.3	[87]

Pharmaceutical	Country	Year	Sampling Type/Locations	Number Of Samples	Method	LOD/LOQ (ng L^{-1})	Frequency (Highest Value) (%)	Average (Highest Value) (ng L^{-1})	Maximum (Highest Value) (ng L^{-1})	Reference
	USA	2014/2015	GS/2	6	SPE-UPLC-MS/MS	NA	NA	ND	ND	[74]
							43.2(100)	26.2(290.0)	18.7(85.0)	
17α-estradiol (α-E2)	France	2014/2015	GS/6	24	SPE-UHPLC-MS/MS	2.2/7.1	NA	NA	NA	[76]
	NA	NA	NA	9	NA	NA	64	0.8	3.1	[19]
	USA	2010	FP/1	6	SPE-LC-MS/MS	1.2/3.5	NA	<LOQ	4700	[22]
							64.0(64.0)	0.4(0.8)	2351.6(4700.0)	
17α-ethinylestradiol (EE2)	Canada	NA	TP/1	NA	SPE-LDTD-APCI-MS/MS	140/203	NA	380	NA	[51]
	EU	2010	GS/90	90	SPE-LC-MS/MS	NA	0	ND	ND	[68]
	Germany	2002	NA/1	18	SPE-LC-MS/MS	NA/0.4	100	1.7	NA	[65]
	International	NA	7	70	NA	NA	NA	0.5	1.9	[66]
	International	NA	NA	NA	NA	NA	NA	3	10	[11]
	Korea	2004/2005	GS/7	7	SPE-LC-MS/MS	NA	14	1.3	1.3	[79]
	Korea	NA	NA	NA	NA	NA	NA	NA	1.3	[75]
	Luxembourg	2007/2008	TP/1	24	SPE-LC-MS/MS	2.0/6.0	0	ND	ND	[60]
	NA	NA	NA	33	NA	NA	59	0.9	5	[19]
	Portugal	NA	GS/5	9	SPE-LC-MS	21/69	0	ND	ND	[53]
	UK	NA	NA	NA	NA	NA	NA	NA	0.47	[43]
	USA	2010	FP/1	6	SPE-LC-MS/MS	1.3/3.8	NA	<LOQ	2.8	[22]
	USA	2012/2013	GS/4	28	SPE-UPLC-MS/MS	6.48/NA	4	NA	11.9	[87]

Pharmaceutical	Country	Year	Sampling Type/Locations	Number Of Samples	Method	LOD/LOQ (ng L^{-1})	Frequency (Highest Value) (%)	Average (Highest Value) (ng L^{-1})	Maximum (Highest Value) (ng L^{-1})	Reference
	USA	2014/2015	GS/2	6	SPE-UPLC-MS/MS	NA	NA	ND	ND	[74]
							25.3(100)	35.2(380.0)	2.9(11.9)	
Total							44.3(100)	23.1(380.0)	123.5(4700.0)	

CS - Composite sampling
ESI- Electrospray ionization
FP - Flow proportional sampling
FD - Fluorescence detection
GC - Gas chromatography
GS - Grab sampling
HPLC - High performance liquid chromatography
HRMS - High resolution mass spectrum
LC - Liquid chromatography
LDTD-APCI - Laser diode thermal desorption atmospheric pressure chemical ionization
LPME - Liquid-phase microextraction
LOQ - Limit of quantification
MS - Mass spectrometry
NA - Not available
ND - Not detected
SPE - Solid phase extraction
TF - TurboFlow
TOF - Time-of-flight
TP - Time proportional sampling
UPLC - Ultra performance liquid chromatography
UHPLC - Ultra high performance liquid chromatography

Table S4. Occurrence of pharmaceuticals in surface waters (SWs).

Pharmaceutical	Country	Year	Sampling type/locations	Number of samples	Method	LOD/LOQ (ng L^{-1})	Frequency (highest value) (%)	Average (highest value) (ng L^{-1})	Maximum (highest value) (ng L^{-1})	Reference
Anxiolytics										
Alprazolam (ALP)	China	2014	GS/12	12	SPE-LC–MS/MS	NA/0.1	100	2.5	4.3	[4]
	Portugal	2009	GS/6	12	SPE-LC-MS/MS	3/10	0	ND	ND	[90]
	Portugal	2016	GS/31	31	UHPLC-TOF-MS	NA/NA	3.2	NA	NA	[91]
							34.4(100)	1.3(2.5)	2.2(4.3)	
Lorazepam (LOR)	China	2015	GS/12	12	SPE-LC–MS/MS	NA/4.5	100	4.0	5.7	[4]
	France	2007/2008	GS/8	24	SPE-LC-MS/MS	NA/12	29	NA	1.7	[92]
	Italy	2011	CS/2	6	SPE-LC-MS/MS	NA	NA	NA	50	[71]
	Portugal	2009	GS/6	12	SPE-LC-MS/MS	4/13	35	34.3	49.1	[90]
	Serbia	NA	GS/14	14	SPE-LC-MS/MS	0.55/1.82	0	ND	ND	[72]
	Spain	2010	NA/10	10	TF-LC-MS/MS	2.8/9.3	NA	NA	50.2	[93]
	Spain	NA	NA/5	5	SPE-UPLC-MS/MS	0.8/2.6	0	ND	ND	[94]
							32.8(100)	9.6(34.3)	22.4(50.2)	
Zolpidem (ZOL)	Portugal	2009	GS/6	12	SPE-LC-MS/MS	2/5	0	ND	ND	[90]
							0.0(0)	0.0(0)	0.0(0)	
Total							29.7(100)	5.8(34.3)	16.1(50.2)	
Antibiotics										
Azithromycin (AZI)	China	2009	GS/10	23	SPE-LC-MS/MS	NA/0.26	91	22.3	88	[95]

Pharmaceutical	Country	Year	Sampling type/locations	Number of samples	Method	LOD/LOQ (ng L^{-1})	Frequency (highest value) (%)	Average (highest value) (ng L^{-1})	Maximum (highest value) (ng L^{-1})	Reference
	China	NA	NA	NA	NA	NA	NA	NA	4.7	[96]
	Croatia	2012/2014	GS/38	148	SPE-LC-ESI-MS/MS	NA/200	NA	NA	1100	[97]
	Germany	2001/2002	NA/8	8	SPE-LC-MS/MS	NA/1	50	4	13	[98]
	Ghana	2010/2011	GS/19	26	SPE-LC-MS/MS	NA/NA	8	14	27	[99]
	International	NA	NA	NA	NA	NA	40.8	188	1547	[100]
	Italy	2011	CS/2	6	SPE-LC-MS/MS	NA	NA	NA	7	[71]
	Italy	NA	CP/1	4	SPE-LC-MS/MS	1/NA	NA	7	NA	[17]
	Japan	2005	NA/7	7	SPE-LC-MS/MS	NA/0.5	14	208	448	[101]
	Japan	2006/2007	GS/37	37	SPE-LC-MS/MS	NA/0.03	49	30	138	[18]
	Kenya	2011	GS/12	12	SPE-LC-MS/MS	NA/NA	0	ND	ND	[99]
	Mozambique	2013	GS/7	7	SPE-LC-MS/MS	NA/NA	14	6	6	[99]
	Pakistan	2012	GS/19	40	LC-MS/MS	NA/NA	23	11.4	21	[102]
	Pakistan (near drug facilities)	2012	GS/4	4	LC-MS/MS	NA/NA	100	13.8	35	[102]
	Portugal	2009	GS/6	12	SPE-LC-MS/MS	4/13	33	135.2	163	[90]
	Portugal	2016	GS/31	31	UHPLC-TOF-MS	NA/NA	9.7	NA	NA	[91]
	Serbia	NA	GS/14	14	SPE-LC-MS/MS	2.58/8.59	21	57	81	[72]
	South Africa	2012	GS/16	16	SPE-LC-MS/MS	NA/NA	25	1.7	3.3	[99]

Pharmaceutical	Country	Year	Sampling type/locations	Number of samples	Method	LOD/LOQ (ng L^{-1})	Frequency (highest value) (%)	Average (highest value) (ng L^{-1})	Maximum (highest value) (ng L^{-1})	Reference
	Spain	2005/2006	NA/7	21	SPE-LC-MS/MS	1/NA	0	ND	ND	[103]
	Spain	2010	NA/10	10	TF-LC-MS/MS	3.2/10.6	NA	NA	41.1	[93]
	Spain	2011	GS/5	5	SPE-UPLC-MS/MS	0.6/2.1	NA	NA	130	[21]
	Portugal	2013/2014	GS/5	55	SPE-UHPLC-MS/MS	0.04/0.10	18.2	NA	29.6	[20]
	Spain	NA	NA/10	10	SPE-LC-MS/MS	1/3	NA	8	20	[14]
	Spain	NA	5	5	SPE-UPLC-MS/MS	0.2/0.5	0	ND	ND	[94]
	Spain	NA	5	5	SPE-UPLC-MS/MS	0.7/2.3	0	ND	ND	[94]
	USA	2001	NA/74	74	SPE-LC-MS/MS	23/NA	1.4	ND	29	[104]
	USA	2008/2009	GS/29	42	SPE-LC-MS/MS	2.5/3.6	52	382	2800	[105]
	USA	2008/2009	GS/7	42	SPE-LC-MS/MS	3.7/11.0	23.8	10.7	21.7	[106]
	USA	2014/2015	GS/2	12	SPE-UPLC-MS/MS	NA	NA	ND	ND	[74]
	Vietnam	2005	NA/4	4	SPE-LC-MS/MS	NA/0.5	0	ND	ND	[101]
	Vietnam	2007	NA/10	20	SPE-LC-MS/MS	NA/7.0	5	90.8	90.8	[107]
							25.2(100)	49.6(382.0)	236.0(2800.0)	
Clarithromycin (CLA)	China	2009	GS/10	23	SPE-LC-MS/MS	NA/0.25	87	5.0	32.9	[95]
	China	2012/2013	GS/20	20	SPE-LC-MS/MS	NA/NA	85	15.0	103	[108]

Pharmaceutical	Country	Year	Sampling type/locations	Number of samples	Method	LOD/LOQ (ng L^{-1})	Frequency (highest value) (%)	Average (highest value) (ng L^{-1})	Maximum (highest value) (ng L^{-1})	Reference
	China	NA	NA	NA	NA	NA	NA	NA	180	[96]
	Germany	2001/2002	NA/8	8	SPE-LC-MS/MS	NA/0.5	75	37	37	[98]
	Ghana	2010/2011	GS/19	26	SPE-LC-MS/MS	NA/NA	50	0.3	26	[99]
	International	NA	NA	NA	NA	NA	53.9	16.5	260	[100]
	Italy	2001	GS/8	8	SPE-LC-MS/MS	0.01/0.02	100	4.7	20.3	[109]
	Italy	2007/2008	TP/8	8	SPE-LC-MS/MS	NA/NA	100	13.6	44.8	[23]
	Italy	2011	CS/2	6	SPE-LC-MS/MS	NA	NA	NA	100	[71]
	Italy	NA	CP/1	4	SPE-LC-MS/MS	1/NA	NA	6	NA	[17]
	Japan	2005	NA/7	7	SPE-LC-MS/MS	NA/0.1	100	143.2	254	[101]
	Japan	2006/2007	GS/37	37	SPE-LC-MS/MS	NA/0.1	100	45	233	[18]
	Kenya	2011	GS/12	12	SPE-LC-MS/MS	NA/NA	83	87	439	[99]
	Korea	2006	NA/5	5	SPE-LC-MS/MS	1/4	60	202	443	[110]
	Korea	2016	GS/18	72	SPE-LC–MS/MS	NA/10.9	43.1	42.7	475.1	[111]
	Mozambique	2013	GS/7	7	SPE-LC-MS/MS	NA/NA	100	6.1	13	[99]
	Pakistan	2012	GS/19	40	LC-MS/MS	NA/NA	100	19.8	130	[102]
	Pakistan (near drug facilities)	2012	GS/4	4	LC-MS/MS	NA/NA	100	110	360	[102]
	Portugal	2013	GS/NA	NA	SPE-UPLC-MS/MS	NA/NA	NA	NA	32	[112]

Pharmaceutical	Country	Year	Sampling type/locations	Number of samples	Method	LOD/LOQ (ng L-1)	Frequency (highest value) (%)	Average (highest value) (ng L-1)	Maximum (highest value) (ng L-1)	Reference
	Portugal	2013/2014	GS/5	55	SPE-UHPLC-MS/MS	0.08/0.25	12.7	NA	26.8	[20]
	South Africa	2012	GS/16	16	SPE-LC-MS/MS	NA/NA	100	28	302	[99]
	Spain	2010	NA/10	10	TF-LC-MS/MS	4.9/16.3	100	NA	141	[93]
	Spain	2011	GS/5	5	SPE-UPLC-MS/MS	0.4/1.2	NA	NA	125	[21]
	Spain	NA	5	5	SPE-UPLC-MS/MS	0.3/0.9	80	16.8	32.8	[94]
	USA	2008/2009	GS/29	42	SPE-LC-MS/MS	2.6/4.1	14	117	370	[105]
	USA	2008/2009	GS/7	42	SPE-LC-MS/MS	3.2/9.6	0	ND	ND	[106]
	Vietnam	2005	NA/4	4	SPE-LC-MS/MS	NA/0.1	0	ND	ND	[101]
	Vietnam	2007	NA/10	20	SPE-LC-MS/MS	NA/0.2	40	205	778	[107]
							68.9(100)	50.9(205.0)	183.7(778.0)	
Ciprofloxacin (CIP)	America	NA	NA	NA	NA	NA	NA	NA	77	[75]
	Canada	2006/2007	GS/17	125	SPE-LC-MS/MS	NA	0	ND	ND	[113]
	China	2009	GS/10	23	SPE-LC-MS/MS	NA/3.3	91	101	346	[95]
	China	NA	NA	NA	NA	NA	NA	NA	459	[96]
	Germany	2001/2002	NA/8	8	SPE-LC-MS/MS	NA/5.0	12.5	9	9	[98]
	India (near drug facilities)	2008	GS/11	NA	SPE-LC-MS	NA/10	NA	NA	650 000	[77]
	International	NA	NA	NA	NA	NA	33.4	163 674	650 000	[100]

Pharmaceutical	Country	Year	Sampling type/locations	Number of samples	Method	LOD/LOQ (ng L^{-1})	Frequency (highest value) (%)	Average (highest value) (ng L^{-1})	Maximum (highest value) (ng L^{-1})	Reference
	Italy	2001	GS/8	8	SPE-LC-MS/MS	0.08/0.27	25	20.3	26.2	[109]
	Italy	2007/2008	TP/8	8	SPE-LC-MS/MS	NA/NA	100	13.9	15.5	[23]
	Italy	2011	CS/2	6	SPE-LC-MS/MS	NA	NA	NA	70	[71]
	Italy	NA	CS/1	4	SPE-LC-MS/MS	1/NA	NA	25	NA	[17]
	Korea	2007	GS/5	5	SPE-LC-MS/MS	NA	0	ND	ND	[27]
	Lebanon	2016	GS/18	18	SPE-LC-MS/MS	29.9/NA	5.5	108	NA	[114]
	Pakistan	2012	GS/19	40	LC-MS/MS	NA/NA	62	16.9	110	[102]
	Pakistan (near drug facilities)	2012	GS/4	4	LC-MS/MS	NA/NA	100	1265	6200	[102]
	Portugal	2006/2007	GS/22	22	SPE-LC-FD	ns/25	36	NA	119.2	[115]
	Portugal	2009	GS/6	12	SPE-LC-MS/MS	10/33	8	59.3	59.3	[90]
	Portugal	2013/2014	GS/5	55	SPE-UHPLC-MS/MS	1.20/3.64	1.82	88.7	88.7	[20]
	Portugal	2016	GS/31	31	UHPLC-TOF-MS	NA/NA	29.0	3.9	7.1	[91]
	Spain	2010	NA/10	10	TF-LC-MS/MS	7.0/23.4	NA	NA	<LOQ	[93]
	Spain	2011	GS/5	5	SPE-UPLC-MS/MS	1.7/5.7	NA	NA	250	[21]
	Spain	2016/2017	GS/23	321	SPE-LC-MS/MS	NA/0.3	21.25	29.25	540	[78]
	USA	1999/2000	CS/NA	115	SPE-UPLC-MS/MS	20/NA	2.6	20	30	[116]

**Pharmaceutical**	**Country**	**Year**	**Sampling type/locations**	**Number of samples**	**Method**	**LOD/LOQ (ng L^{-1})**	**Frequency (highest value) (%)**	**Average (highest value) (ng L^{-1})**	**Maximum (highest value) (ng L^{-1})**	**Reference**
	USA	2001	NA/74	74	SPE-LC-MS/MS	20/NA	1.4	ND	30	[104]
	USA	2008/2009	GS/7	42	SPE-LC-MS/MS	3.3/9.9	0	ND	ND	[106]
	USA	NA	NA	NA	NA	NA	NA	NA	30	[117]
							29.4(100)	9 190.8(163 674)	54 519.5(650 000)	
Erythromycin (ERY)	Canada	2006/2007	GS/17	125	SPE-LC-MS/MS	NA/10	10	0.4	145	[113]
	China	2009	GS/10	23	SPE-LC-MS/MS	NA/0.23	100	62.1	282	[95]
	China	2012/2013	GS/20	20	SPE-LC-MS/MS	NA/NA	95	239.6	808	[108]
	China	NA	NA	NA	NA	NA	NA	NA	1540	[96]
	Croatia	2012/2014	GS/38	148	SPE-LC-ESI-MS/MS	NA/200	NA	NA	5300	[97]
	Germany	2001/2002	NA/8	8	SPE-LC-MS/MS	NA/2.0	100	72.8	190	[98]
	Ghana	2010/2011	GS/19	26	SPE-LC-MS/MS	NA/NA	81	115	645	[99]
	International	NA	NA	NA	NA	NA	55.5	51	90 000	[100]
	Italy	2001	GS/8	8	SPE-LC-MS/MS	0.08/0.29	100	4.7	15.9	[109]
	Italy	2007/2008	TP/8	8	SPE-LC-MS/MS	NA/NA	100	10.8	30.5	[23]
	Italy	NA	CP/1	4	SPE-LC-MS/MS	4/NA	0	ND	ND	[17]
	Japan	2005	NA/7	7	SPE-LC-MS/MS	NA/0.6	14	76.8	120	[101]
	Japan	2006/2007	GS/37	37	SPE-LC-MS/MS	NA/0.02	95	20	123	[18]

Pharmaceutical	Country	Year	Sampling type/locations	Number of samples	Method	LOD/LOQ (ng L^{-1})	Frequency (highest value) (%)	Average (highest value) (ng L^{-1})	Maximum (highest value) (ng L^{-1})	Reference
	Japan	2006/2010	GS/8	27	SPE-LC-MS/MS	NA/10.0	0	ND	ND	[118]
	Japan	NA	NA	NA	NA	NA	NA	NA	450	[117]
	Kenya	2011	GS/12	12	SPE-LC-MS/MS	NA/NA	100	679	2800	[99]
	Korea	2004/2005	GS/3	8	SPE-LC-MS/MS	NA	63	3.4	4.8	[79]
	Korea	2006	NA/5	5	SPE-LC-MS/MS	1/4	80	65	137	[110]
	Korea	2007	GS/5	5	SPE-LC-MS/MS	NA	NA	NA	150	[27]
	Lebanon	2016	GS/18	18	SPE-LC-MS/MS	27/NA	16.6	603	2806	[114]
	Mozambique	2013	GS/7	7	SPE-LC-MS/MS	NA/NA	100	903	2700	[99]
	Netherlands	2009	GS/14	14	SPE-LC-MS/MS	NA	29	10	35	[119]
	Pakistan	2012	GS/19	40	LC-MS/MS	NA/NA	93	39.2	310	[102]
	Pakistan (near drug facilities)	2012	GS/4	4	LC-MS/MS	NA/NA	100	298	1100	[102]
	Portugal	2016	GS/31	31	UHPLC-TOF-MS	NA/NA	0.0	0.0	0.0	[91]
	Scotland	2005/2006	GS/4	40	SPE-LC-MS/MS	0.1/0.3	0	ND	ND	[80]
	Serbia	NA	GS/14	14	SPE-LC-MS/MS	1.65/5.48	0	ND	ND	[72]
	South Africa	2012	GS/16	16	SPE-LC-MS/MS	NA/NA	100	136	493	[99]
	South Africa	2013	GS/12	12	SPE-LC-MS/MS	0.4/0.4	42	130	260	[34]
	Spain	2005/2006	NA/7	21	SPE-LC-MS/MS	4/NA	60	30	70	[103]

Pharmaceutical	Country	Year	Sampling type/locations	Number of samples	Method	LOD/LOQ (ng L-1)	Frequency (highest value) (%)	Average (highest value) (ng L-1)	Maximum (highest value) (ng L-1)	Reference
	Spain	2006	GS/8	16	SPE-LC-MS/MS	NA	93	32.9	111.9	[120]
	Spain	2010	NA/10	10	TF-LC-MS/MS	7.0/23.4	NA	NA	1.0	[93]
	Spain	NA	NA/10	10	SPE-LC-MS/MS	4/14	NA	17	30	[14]
	Spain	NA	NA/7	7	SPE-LC-MS/MS	NA	86	21	46.7	[121]
	Spain	NA	5	5	SPE-UPLC-MS/MS	6.7/22.4	0	ND	ND	[94]
	Taiwan	2007/2008	GS/15	15	SPE-LC-MS/MS	1.0/NA	92	NA	75 500	[122]
	UK	2007	GS/2	40	SPE-LC-MS/MS	NA	51	18	131	[35]
	UK	NA	GS/2	2	SPE-LC-MS/MS	10/NA	100	529	1000	[81]
	UK	NA	NA	NA	NA	NA	NA	NA	1022	[36]
	USA	1999/2000	CS/NA	104	SPE-UPLC-MS/MS	50/NA	21.5	100	1700	[116]
	USA	2001	NA/74	73	SPE-LC-MS/MS	50/NA	8.1	ND	300	[104]
	USA	2014/2015	GS/2	12	SPE-UPLC-MS/MS	NA	NA	ND	ND	[74]
	Vietnam	2005	NA/4	4	SPE-LC-MS/MS	NA/0.6	100	26.9	41	[101]
	Vietnam	2007	NA/10	20	SPE-LC-MS/MS	NA/1.0	60	518	2246	[107]
							59.6(100)	130.1(903.0)	4378.3(90 000)	
Total							48.4(100)	1708.5(163 674)	12 200.9(650 000)	
Lipid regulators										

Pharmaceutical	Country	Year	Sampling type/locations	Number of samples	Method	LOD/LOQ (ng L^{-1})	Frequency (highest value) (%)	Average (highest value) (ng L^{-1})	Maximum (highest value) (ng L^{-1})	Reference
Bezafibrate (BEZ)	Brazil	1996	TP/17	17	SPE-GC-MS	35/NA	NA	NA	180	[38]
	Canada	2006/2007	GS/17	125	SPE-LC-MS/MS	NA/0.5	10	0.2	3.6	[113]
	China	NA	NA	NA	NA	NA	NA	NA	20	[96]
	France	2007/2008	GS/8	24	SPE-LC-MS/MS	NA/1	63	NA	12.4	[92]
	Germany	2007	TP/2	36	SPE-LC-MS/MS	NA/12	NA	71	280	[123]
	International	NA	NA	NA	NA	NA	58	85	15 060	[100]
	Italy	2001	GS/8	8	SPE-LC-MS/MS	0.02/0.05	100	8.8	57.2	[109]
	Italy	2011	CS/2	6	SPE-LC-MS/MS	NA	NA	NA	10	[71]
	Japan	2006/2010	GS/8	27	SPE-LC-MS/MS	NA/3.0	48.1	91.2	498	[118]
	Mexico	2015/2016	TP/4	16	SPE- LC-MS/MS	0.3/1.0	100	908.5	2100	[41]
	Netherlands	2009	GS/14	14	SPE-LC-MS/MS	NA	14	5	17	[119]
	Portugal	2009	GS/6	12	SPE-LC-MS/MS	3/10	67	265.9	770	[90]
	Portugal	2016	GS/31	31	UHPLC-TOF-MS	NA/NA	87.1	3.3	13.4	[91]
	Spain	2005/2006	NA/7	21	SPE-LC-MS/MS	1/NA	95	1020	15 060	[103]
	Spain	2006	GS/8	16	SPE-LC-MS/MS	NA	100	48.3	305.2	[120]
	Spain	2010	NA/10	10	TF-LC-MS/MS	0.2/0.6	NA	NA	51.3	[93]
	Spain	2012	GS/22	22	SPE-UPLC-MS/MS	NA/0.7	36	4	21	[42]

Pharmaceutical	Country	Year	Sampling type/locations	Number of samples	Method	LOD/LOQ (ng L^{-1})	Frequency (highest value) (%)	Average (highest value) (ng L^{-1})	Maximum (highest value) (ng L^{-1})	Reference
	Spain	2016/2017	GS/23	321	SPE-LC-MS/MS	NA/0.3	28.5	6.75	67	[78]
	Spain	NA	NA/10	10	SPE-LC-MS/MS	1/4	NA	8	10	[14]
	Spain	NA	NA/7	7	SPE-LC-MS/MS	NA	100	975	5960	[121]
	Spain	NA	NA/5	5	SPE-UPLC-MS/MS	0.1/0.4	80	4.3	7.2	[94]
	Sweden	2014	TP/8	8	SPE-UPLC-MS/MS	NA/0.04	100	20.4	110	[124]
	UK	NA	NA	NA	NA	NA	NA	NA	90	[43]
	UK	2007	GS/2	40	SPE-LC-MS/MS	NA	53	23	64	[35]
							67.0(100)	197.1(1020.0)	1698.6(15 060)	
Gemfibrozil (GEM)	Brazil	1996	TP/17	17	SPE-GC-MS	10/NA	0	ND	ND	[38]
	Canada	2005	GS/2	8	SPE-GC-MS	0.3/NA	55.6	14.5	35.3	[83]
	Canada	2006/2007	GS/17	125	SPE-LC-MS/MS	NA/1	33	0.7	9	[113]
	China	2007	GS/14	14	SPE-GC-MS	1.8/6.1	14	15.9	17.4	[125]
	China	2012/2013	GS/20	20	SPE-LC-MS/MS	NA/NA	0	ND	ND	[108]
	China	2013	GS/19	57	SPE-LC-MS/MS	0.01/NA	79	0.1	2.3	[126]
	China	NA	NA	NA	NA	NA	NA	NA	86.8	[96]
	Costa Rica	NA	NA	NA	NA	NA	NA	NA	17 036	[33]
	International	NA	NA	NA	NA	NA	45.3	103	7780	[100]
	Korea	2004/2005	GS/3	8	SPE-LC-MS/MS	NA	34	6.6	9.1	[79]

Pharmaceutical	Country	Year	Sampling type/locations	Number of samples	Method	LOD/LOQ (ng L^{-1})	Frequency (highest value) (%)	Average (highest value) (ng L^{-1})	Maximum (highest value) (ng L^{-1})	Reference
	Korea	2007	GS/5	5	SPE-LC-MS/MS	NA	0	ND	ND	[27]
	Mexico	2015/2016	TP/4	16	SPE- LC-MS/MS	0.2/0.5	100	55.3	368	[41]
	NA	NA	NA	NA	NA	NA	NA	NA	52	[85]
	Portugal	2009	GS/6	12	SPE-LC-MS/MS	10/33	50	57.8	91.2	[90]
	Portugal	2013	GS/NA	NA	SPE-UPLC-MS/MS	NA/NA	NA	NA	16	[112]
	Portugal	2016	GS/31	31	UHPLC-TOF-MS	NA/NA	67.7	23.1	77.0	[91]
	Spain	2005/2006	NA/7	21	SPE-LC-MS/MS	1/NA	100	1420	7780	[103]
	Spain	2006	GS/8	16	SPE-LC-MS/MS	NA	100	242.8	1014.1	[120]
	Spain	2010	NA/10	10	TF-LC-MS/MS	19.8/66.1	NA	NA	113	[93]
	Spain	2012	GS/22	22	SPE-UPLC-MS/MS	NA/1.3	100	77	934	[42]
	Spain	NA	NA/10	10	SPE-LC-MS/MS	1/3	NA	46	60	[14]
	Spain	NA	NA/7	7	SPE-LC-MS/MS	NA	100	1415	5610	[121]
	Spain	NA	NA/5	5	SPE-UPLC-MS/MS	0.6/2.0	80	148.4	286	[94]
	Taiwan	2007/2008	GS/15	15	SPE-LC-MS/MS	1.0/NA	91	NA	122	[122]
	UK	NA	NA	NA	NA	NA	NA	NA	790	[117]
	USA	1999/2000	CS/NA	84	SPE-UPLC-MS/MS	15/NA	3.6	48	790	[116]
	USA	2001	NA/74	74	SPE-LC-MS/MS	15/NA	0	ND	ND	[104]

Pharmaceutical	Country	Year	Sampling type/locations	Number of samples	Method	LOD/LOQ (ng L^{-1})	Frequency (highest value) (%)	Average (highest value) (ng L^{-1})	Maximum (highest value) (ng L^{-1})	Reference
	USA	2006/2007	GS/19	32	SPE-GC/LC-MS/MS	0.25/NA	58	2.2	24	[127]
	USA	2008/2009	GS/7	42	SPE-LC-MS/MS	1.6/4.8	0	ND	ND	[106]
	USA	2014/2015	GS/2	12	SPE-UPLC-MS/MS	NA	NA	15.90	190.80	[74]
	USA	2016	GS/72	72	SPE-UPLC-MS/MS	1.30/NA	56.8	21.2	1440.4	[128]
							50.8(100)	154.7(1420.0)	1443.0(17 036)	
Simvastatin (SIM)	Canada	2002	NA/1	3	SPE-LC-MS/MS	0.1/NA	0	ND	ND	[49]
	Malaysia	2013	GS/3	6	SPE-LC-MS/MS	NA/2	33	6	6	[50]
	Portugal	2009	GS/6	12	SPE-LC-MS/MS	3/10	8	42.9	42.9	[90]
	Portugal	2016	GS/31	31	UHPLC-TOF-MS	NA/NA	0.0	0.0	0.0	[91]
	UK	2007	GS/2	40	SPE-LC-MS/MS	NA	0	ND	ND	[35]
	UK	NA	NA	NA	NA	NA	0	ND	ND	[43]
							6.8(33.0)	8.2(42.9)	8.2(42.9)	
Total							51.1(100)	152.3(1420.0)	1402.5(17 036)	
Antiepileptic										
Carbamazepine (CAR)	America	NA	NA	NA	NA	NA	NA	NA	735	[75]
	Asia	NA	NA	NA	NA	NA	NA	NA	120	[75]
	Bangladesh	2017	GS/20	20	SPE-LC–MS/MS	0.01/0.02	65.0	1.51	8.8	[129]
	Canada	2006/2007	GS/17	125	SPE-LC-MS/MS	NA/1	50	3	749	[113]

Pharmaceutical	Country	Year	Sampling type/locations	Number of samples	Method	LOD/LOQ (ng L^{-1})	Frequency (highest value) (%)	Average (highest value) (ng L^{-1})	Maximum (highest value) (ng L^{-1})	Reference
	China	2012/2013	GS/20	20	SPE-LC-MS/MS	NA/NA	60	0.9	50	[108]
	China	2013	GS/19	57	SPE-LC-MS/MS	0.02/NA	79	0.6	24.5	[126]
	China	2014	GS/12	12	SPE-LC–MS/MS	NA/3.0	100	25.3	75.5	[4]
	Europe	NA	NA	NA	NA	NA	NA	NA	366	[75]
	France	2007/2008	GS/8	24	SPE-LC-MS/MS	NA/1	88	NA	41.6	[92]
	Germany	2007	TP/2	36	SPE-LC-MS/MS	NA	NA	400	730	[123]
	Germany	NA	NA	NA	NA	NA	NA	NA	1194	[33]
	Germany/Canada	NA	NA	NA	NA	NA	NA	NA	1300	[117]
	International	NA	NA	NA	NA	NA	85	174	11 561	[100]
	Italy	2011	CS/2	6	SPE-LC-MS/MS	NA	NA	NA	80	[71]
	Italy	NA	CP/1	4	SPE-LC-MS/MS	1/NA	NA	7	NA	[17]
	Japan	2006/2010	GS/8	27	SPE-LC-MS/MS	NA/0.4	48.1	22.2	100	[118]
	Kenya	2012/2013	GS/3	14	SPE-LC-MS	NA/NA	85.71	204.28	430	[26]
	Korea	2004/2005	GS/3	8	SPE-LC-MS/MS	NA	88	25	61	[79]
	Korea	2006	NA/5	5	SPE-LC-MS/MS	1/4	80	200	595	[110]
	Korea	2007	GS/5	5	SPE-LC-MS/MS	NA	NA	NA	100	[27]
	Korea	2016	GS/18	72	SPE-LC–MS/MS	NA/4.6	97.2	206.7	899.9	[111]
	Malaysia	2013	GS/2	6	SPE-LC-MS/MS	NA/1.6	67	15	24	[50]

Pharmaceutical	Country	Year	Sampling type/locations	Number of samples	Method	LOD/LOQ (ng L^{-1})	Frequency (highest value) (%)	Average (highest value) (ng L^{-1})	Maximum (highest value) (ng L^{-1})	Reference
	Mexico	2015/2016	TP/4	16	SPE- LC-MS/MS	0.8/2.6	100	56.3	276	[41]
	Netherlands	2002	NA	263	NA	NA	NA	NA	227	[130]
	Netherlands	2009	GS/14	14	SPE-LC-MS/MS	NA	86	59	121	[119]
	Portugal	2007/2008	GS/11	87	SPE-LC-MS/MS	0.03/0.4	100	NA	178	[131]
	Portugal	2013	GS/NA	NA	SPE-UPLC-MS/MS	NA/NA	NA	NA	17	[112]
	Portugal	2013/2014	GS/5	55	SPE-UHPLC-MS/MS	0.04/0.12	100	31.7	214	[20]
	Portugal	2016	GS/31	31	UHPLC-TOF-MS	NA/NA	0.0	0.0	0.0	[91]
	Serbia	NA	GS/14	14	SPE-LC-MS/MS	0.27/0.90	93	56	130	[72]
	South Africa	2013	GS/12	12	SPE-LC-MS/MS	89/297	100	428	1650	[34]
	Spain	2005/2006	NA/7	21	SPE-LC-MS/MS	2/NA	90	1070	3090	[103]
	Spain	2006	GS/8	16	SPE-LC-MS/MS	NA	100	64.0	178.7	[120]
	Spain	2010	NA/10	10	TF-LC-MS/MS	0.1/0.5	NA	NA	90.4	[93]
	Spain	2016/2017	GS/23	321	SPE-LC-MS/MS	NA/0.3	50.8	4.3	93	[78]
	Spain	NA	NA/10	10	SPE-LC-MS/MS	2/10	NA	30	110	[14]
	Spain	NA	NA/7	7	SPE-LC-MS/MS	NA	100	965	2680	[121]
	Spain	NA	NA/5	5	SPE-UPLC-MS/MS	0.2/0.8	80	19.7	38.6	[94]

Pharmaceutical	Country	Year	Sampling type/locations	Number of samples	Method	LOD/LOQ (ng L-1)	Frequency (highest value) (%)	Average (highest value) (ng L-1)	Maximum (highest value) (ng L-1)	Reference
	Sweden	2007/2008	GS/3	30	SPE-LC-MS/MS	NA	100	110	NA	[86]
	Sweden	2014	TP/8	8	SPE-UPLC-MS/MS	NA/0.04	100	88.1	310	[124]
	UK	2007	GS/2	40	SPE-LC-MS/MS	NA	95	133	647	[35]
	UK	NA	NA	NA	NA	NA	NA	NA	684	[43]
	USA	2006/2007	GS/19	32	SPE-GC/LC-MS/MS	0.25/NA	63	2.3	36	[127]
	USA	2008/2009	GS/7	42	SPE-LC-MS/MS	2.7/8.2	21.4	14.9	38.2	[106]
	USA	2012/2013	GS/4	28	SPE-UPLC-MS/MS	0.03/NA	7	0.10	0.17	[87]
	USA	2014/2015	GS/2	12	SPE-UPLC-MS/MS	NA	NA	2.04	2.96	[74]
	USA	2016	GS/72	72	SPE-UPLC-MS/MS	0.07/NA	100	6.1	542.6	[128]
							77.5(100)	130.2(1070.0)	680.0(11 561)	
Selective serotonin reuptake inhibitors (SSRIs)										
Citalopram (CIT)	Canada	2007	FP/1	4	SPE-LC-MS/MS	0.077/NA	100	4.6	11.5	[55]
	India (near drug facilities)	2008	GS/11	NA	SPE-LC-MS	NA/10	NA	NA	76 000	[77]
	International	NA	NA	NA	NA	NA	100	20	219	[100]
	Portugal	2013/2014	GS/5	55	SPE-UHPLC-MS/MS	0.08/0.24	10.9	0.86	28.9	[20]
	Spain	NA	NA/5	5	SPE-UPLC-MS/MS	0.3/1.1	20	0.9	4.7	[94]

Pharmaceutical	Country	Year	Sampling type/locations	Number of samples	Method	LOD/LOQ (ng L^{-1})	Frequency (highest value) (%)	Average (highest value) (ng L^{-1})	Maximum (highest value) (ng L^{-1})	Reference
							57.7(100)	6.6(20.0)	15 252.8(76 000)	
Fluoxetine (FLU)	Canada	2007	FP/1	4	SPE-LC-MS/MS	0.050/NA	100	0.9	1.3	[55]
	China	2013	GS/19	57	SPE-LC-MS/MS	0.04/NA	0	ND	ND	[126]
	China	2014	GS/12	12	SPE-LC–MS/MS	NA/0.3	100	0.4	1.3	[4]
	England	NA	GS/7	69	SPE-LC-MS/MS	NA	9	7.6	13.5	[43]
	France	2007/2008	GS/8	24	SPE-LC-MS/MS	NA/85	0	ND	ND	[92]
	International	NA	NA	NA	NA	NA	29.2	18	596	[100]
	Korea	2004/2005	GS/3	8	SPE-LC-MS/MS	NA	0	ND	ND	[79]
	NA	NA	NA	NA	NA	NA	NA	NA	30	[85]
	Portugal	2009	GS/6	12	SPE-LC-MS/MS	6/20	0	ND	ND	[90]
	Portugal	2013/2014	GS/5	55	SPE-UHPLC-MS/MS	0.02/0.08	100	3.25	19.5	[20]
	Portugal	2016	GS/31	31	UHPLC-TOF-MS	NA/NA	22.6	9.9	16.2	[91]
	Spain	2005/2006	NA/7	21	SPE-LC-MS/MS	20/NA	0	ND	ND	[103]
	Spain	2006	GS/8	16	SPE-LC-MS/MS	NA	7	4.2	4.2	[120]
	Spain	2010	NA/10	10	TF-LC-MS/MS	3.7/12.5	NA	NA	14.5	[93]
	Spain	NA	NA/10	10	SPE-LC-MS/MS	20/66	NA	ND	ND	[14]
	Spain	NA	NA/5	5	SPE-UPLC-MS/MS	0.8/2.6	0	ND	ND	[94]

Pharmaceutical	Country	Year	Sampling type/locations	Number of samples	Method	LOD/LOQ (ng L^{-1})	Frequency (highest value) (%)	Average (highest value) (ng L^{-1})	Maximum (highest value) (ng L^{-1})	Reference
	UK	NA	NA	NA	NA	NA	NA	NA	14	[36]
	USA	1999/2000	CS/NA	84	SPE-UPLC-MS/MS	18/NA	1.2	12	12	[116]
	USA	2001	NA/74	74	SPE-LC-MS/MS	18/NA	1.4	NA	<LOQ	[104]
	USA	2006/2007	GS/19	32	SPE-GC/LC-MS/MS	0.50/NA	16	0.8	3	[127]
	USA	2008/2009	GS/7	42	SPE-LC-MS/MS	3.5/10.5	9.5	23.2	91.6	[106]
	USA	2014/2015	GS/2	12	SPE-UPLC-MS/MS	NA	NA	1.44	17.31	[74]
							23.3(100)	4.5(23.2)	37.9(596.0)	
Norfluoxetine (Nor-FLU)	Canada	2007	FP/1	4	NA	NA	30	1.3	1.3	[55]
	England	NA	GS/7	69	0	ND	ND	1.9	3.5	[43]
	France	2007/2008	GS/8	24	7	4.2	4.2	ND	ND	[92]
	Portugal	2013/2014	GS/5	55	SPE-UHPLC-MS/MS	0.22/0.64	0	0	0	[20]
	UK	NA	NA	NA	NA	NA	14.5	NA	3.5	[36]
	USA	2006/2007	GS/19	32	0	ND	ND	ND	ND	[127]
							8.1(30)	0.6(1.9)	1.4(3.5)	
Paroxetine (PAR)	Canada	2007	FP/1	4	SPE-LC-MS/MS	0.096/NA	100	2.2	3.0	[55]
	Japan	2006/2010	GS/8	27	SPE-LC-MS/MS	NA/8.0	0	ND	ND	[118]
	Portugal	2009	GS/6	12	SPE-LC-MS/MS	3/10	0	ND	ND	[90]

Pharmaceutical	Country	Year	Sampling type/locations	Number of samples	Method	LOD/LOQ (ng L^{-1})	Frequency (highest value) (%)	Average (highest value) (ng L^{-1})	Maximum (highest value) (ng L^{-1})	Reference
	Portugal	2013/2014	GS/5	55	SPE-UHPLC-MS/MS	6.40/19.4	5.45	25.5	25.6	[20]
	Spain	2005/2006	NA/7	21	SPE-LC-MS/MS	8/NA	0	ND	ND	[103]
	Spain	2006	GS/8	16	SPE-LC-MS/MS	NA	0	ND	ND	[120]
	Spain	2010	NA/10	10	TF-LC-MS/MS	4.6/15.2	NA	NA	7.8	[93]
	Spain	NA	NA/10	10	SPE-LC-MS/MS	8/20	NA	ND	ND	[14]
	Spain	NA	NA/5	5	SPE-UPLC-MS/MS	0.6/2.0	0	ND	ND	[94]
	USA	2014/2015	GS/2	12	SPE-UPLC-MS/MS	NA	NA	0.64	2.48	[74]
							15.1(100)	3.1(25.5)	3.9(25.6)	
Sertraline (SER)	Canada	2007	FP/1	4	SPE-LC-MS/MS	0.048/NA	100	1.6	2.4	[55]
	Portugal	2013/2014	GS/5	55	SPE-UHPLC-MS/MS	0.02/0.06	27.3	0.01	NA	[20]
	Portugal	2016	GS/31	31	UHPLC-TOF-MS	NA/NA	6.5	202	304	[91]
	Spain	NA	NA/5	5	SPE-UPLC-MS/MS	1.7/5.7	0	ND	ND	[94]
							33.5(100)	67.9(202)	102.1(304)	
Desmethylsertraline (Nor-SER)	Canada	2007	FP/1	4	SPE-LC-MS/MS	0.072/NA	100	2.3	4.5	[55]
							100.0(100)	2.3(2.3)	4.5(4.5)	
Total							26.0(100)	8.6(202.0)	1648.0(76 000)	

Pharmaceutical	Country	Year	Sampling type/locations	Number of samples	Method	LOD/LOQ (ng L^{-1})	Frequency (highest value) (%)	Average (highest value) (ng L^{-1})	Maximum (highest value) (ng L^{-1})	Reference
Anti-inflammatories										
Diclofenac (DIC)	Asia	NA	NA	NA	NA	NA	NA	NA	62	[75]
	Brazil	1996	TP/17	17	SPE-GC-MS	10/NA	NA	20	450	[38]
	Canada	2005	GS/2	8	SPE-GC-MS	1.0/NA	0	ND	ND	[83]
	Canada	2006/2007	GS/17	125	SPE-LC-MS/MS	NA	0	ND	ND	[113]
	China	2007	GS/14	14	SPE-GC-MS	1.1/4.4	79	51.2	147	[125]
	China	2012/2013	GS/20	20	SPE-LC-MS/MS	NA/NA	0	ND	ND	[108]
	China	2013	GS/19	57	SPE-LC-MS/MS	0.18/NA	100	12.9	58.4	[126]
	China	NA	NA	NA	NA	NA	NA	NA	7.7	[96]
	Europe	NA	NA	NA	NA	NA	NA	NA	794	[75]
	France	2007/2008	GS/8	24	SPE-LC-MS/MS	NA/1	75	NA	35.0	[92]
	Germany	2002	GS/7	7	SPE-GC-MS	0.08/NA	100	38.4	67	[132]
	Germany	2007	TP/2	36	SPE-LC-MS/MS	NA/45	NA	410	1400	[123]
	Germany	NA	NA	NA	NA	NA	NA	NA	14 000	[117]
	Greece	NA	NA	NA	NA	NA	NA	NA	1043	[33]
	International	NA	NA	NA	NA	NA	75.5	137	18 740	[100]
	Italy	2011	CS/2	6	SPE-LC-MS/MS	NA	NA	NA	60	[71]
	Japan	2006/2010	GS/8	27	SPE-LC-MS/MS	NA/2.5	3.7	44.0	44.0	[118]
	Kenya	2012/2013	GS/3	14	SPE-LC-MS	NA/NA	78.57	277.85	730	[26]

Pharmaceutical	Country	Year	Sampling type/locations	Number of samples	Method	LOD/LOQ (ng L^{-1})	Frequency (highest value) (%)	Average (highest value) (ng L^{-1})	Maximum (highest value) (ng L^{-1})	Reference
	Korea	2004/2005	GS/3	8	SPE-LC-MS/MS	NA	38	3.0	6.8	[79]
	Korea	2007	GS/5	5	SPE-LC-MS/MS	NA	0	ND	ND	[27]
	Luxembourg	2007/2008	TP/1	24	SPE-LC-MS/MS	0.3/1.0	NA	NA	55	[60]
	Malaysia	2013	GS/2	6	SPE-LC-MS/MS	NA/12	50	39	54	[50]
	Mexico	2015/2016	TP/4	16	SPE- LC-MS/MS	1.2/4.1	100	740	1398	[41]
	NA	NA	NA	NA	NA	NA	NA	NA	1800	[85]
	Portugal	2009	GS/6	12	SPE-LC-MS/MS	5/17	17	39.8	56.8	[90]
	Portugal	2013	GS/NA	NA	SPE-UPLC-MS/MS	NA/NA	NA	NA	1	[112]
	Portugal	2013/2014	GS/5	55	SPE-UHPLC-MS/MS	0.10/0.30	1.82	38	38	[20]
	Portugal	2016	GS/31	31	UHPLC-TOF-MS	NA/NA	32.3	14.8	51.8	[91]
	Scotland	2005/2006	GS/4	40	SPE-LC-MS/MS	0.2/0.6	17.5	0.14	1.9	[80]
	Serbia	NA	GS/14	14	SPE-LC-MS/MS	0.15/0.49	0	ND	ND	[72]
	South Africa	2015/2016	TP/7	38	SPE-GC-MS	484/1614	NA	NA	10200	[133]
	Spain	2005/2006	NA/7	21	SPE-LC-MS/MS	2/NA	100	2200	18 740	[103]
	Spain	2006	GS/8	16	SPE-LC-MS/MS	NA	100	87.7	358.1	[120]
	Spain	2010	NA/10	10	TF-LC-MS/MS	10.2/34.0	NA	NA	260	[93]

Pharmaceutical	Country	Year	Sampling type/locations	Number of samples	Method	LOD/LOQ (ng L^{-1})	Frequency (highest value) (%)	Average (highest value) (ng L^{-1})	Maximum (highest value) (ng L^{-1})	Reference
	Spain	2012	GS/22	22	SPE-UPLC-MS/MS	NA/3.9	50	49	865	[42]
	Spain	2016/2017	GS/23	321	SPE-LC-MS/MS	NA/0.3	45.3	23.8	650	[78]
	Spain	NA	NA/10	10	SPE-LC-MS/MS	2/5	NA	29	60	[14]
	Spain	NA	NA/7	7	SPE-LC-MS/MS	NA	100	2204	11 200	[121]
	Spain	NA	5	5	SPE-UPLC-MS/MS	1.4/4.6	60	14.9	29.1	[94]
	Spain/Belgium/Germany	NA	TP/NA	16	SPE-GC/LC-MS/MS	7/20	NA	NA	72	[62]
	Sweden	2014	TP/8	8	SPE-UPLC-MS/MS	NA/0.04	100	126.4	490	[124]
	UK	2007	GS/2	40	SPE-LC-MS/MS	NA	96	29	261	[35]
	UK	NA	NA	NA	NA	NA	NA	NA	568	[36]
	USA	2006/2007	GS/19	32	SPE-GC/LC-MS/MS	0.25/NA	21	1.1	1.2	[127]
							51.5(100)	221.0(2204.0)	1928.5(18 740)	
4-hydroxydiclofenac (4-OH-DIC)	Spain	2010	NA/10	10	TF-LC-MS/MS	0.3/0.9	NA	NA	39.8	[93]
	Sweden	2014	TP/8	8	SPE-UPLC-MS/MS	NA/0.04	0	ND	ND	[124]
							0	0	19.9(39.8)	
Ibuprofen (IBU)	America	NA	NA	NA	NA	NA	NA	NA	203	[75]
	Asia	NA	NA	NA	NA	NA	NA	NA	30	[75]
	Brazil	1996	TP/17	17	SPE-GC-MS	10/NA	NA	NA	190	[38]

Pharmaceutical	Country	Year	Sampling type/locations	Number of samples	Method	LOD/LOQ (ng L^{-1})	Frequency (highest value) (%)	Average (highest value) (ng L^{-1})	Maximum (highest value) (ng L^{-1})	Reference
	Canada	2005	GS/2	8	SPE-GC-MS	0.8/NA	22.2	7.7	9.5	[83]
	Canada	2006/2007	GS/17	125	SPE-LC-MS/MS	NA/0.5	21	0.98	79	[113]
	Canada	2010	GS/9	9	SPE-GC-MS/MS	7/NA	0	ND	ND	[88]
	China	2007	GS/14	14	SPE-GC-MS	0.7/2.2	86	129	490	[125]
	China	2012/2013	GS/20	20	SPE-LC-MS/MS	NA/NA	10	9.1	99.3	[108]
	China	2013	GS/19	57	SPE-LC-MS/MS	0.10/NA	100	12.6	242	[126]
	China	NA	NA	NA	NA	NA	NA	NA	685	[96]
	Costa Rica	NA	NA	NA	NA	NA	NA	NA	36 788	[33]
	Europe	NA	NA	NA	NA	NA	NA	NA	468	[75]
	France	2007/2008	GS/8	24	SPE-LC-MS/MS	NA/7	63	NA	8.0	[92]
	Germany	2002	GS/7	7	SPE-GC-MS	0.05/NA	100	10.5	32	[132]
	Germany	2007	TP/2	36	SPE-LC-MS/MS	NA/8	NA	16	120	[123]
	International	NA	NA	NA	NA	NA	63	504	31 323	[100]
	Italy	2001	GS/8	8	SPE-LC-MS/MS	2.2/4.2	63	21.0	78.5	[109]
	Japan	2006/2010	GS/8	27	SPE-LC-MS/MS	NA/1.1	44.4	8.6	33.0	[118]
	Kenya	2012/2013	GS/3	14	SPE-LC-MS	NA/NA	71.42	3712.1	17440	[26]
	Korea	2004/2005	GS/3	8	SPE-LC-MS/MS	NA	75	28	38	[79]
	Korea	2006	NA/5	5	SPE-LC-MS/MS	5/15	80	260	414	[110]

Pharmaceutical	Country	Year	Sampling type/locations	Number of samples	Method	LOD/LOQ (ng L^{-1})	Frequency (highest value) (%)	Average (highest value) (ng L^{-1})	Maximum (highest value) (ng L^{-1})	Reference
	Korea	2007	GS/5	5	SPE-LC-MS/MS	NA	NA	NA	50	[27]
	Luxembourg	2007/2008	TP/1	24	SPE-LC-MS/MS	0.3/1.0	100	NA	2382	[60]
	Mexico	2015/2016	TP/4	16	SPE- LC-MS/MS	1.1/3.7	NA	520	1106	[41]
	NA	NA	NA	NA	NA	NA	NA	NA	2700	[85]
	Portugal	2009	GS/6	12	SPE-LC-MS/MS	50/165	0	ND	ND	[90]
	Portugal	2013	GS/NA	NA	SPE-UPLC-MS/MS	NA/NA	NA	NA	23	[112]
	Portugal	2013/2014	GS/5	55	SPE-UHPLC-MS/MS	4.50/13.6	100	53.7	1317	[20]
	Portugal	2016	GS/31	31	UHPLC-TOF-MS	NA/NA	6.5	NA	NA	[91]
	Scotland	2005/2006	GS/4	40	SPE-LC-MS/MS	1.0/3.2	2.5	1.3	1.3	[80]
	Serbia	NA	GS/14	14	SPE-LC-MS/MS	3.28/10.93	0	ND	ND	[72]
	South Africa	2013	GS/12	12	SPE-LC-MS/MS	81/271	100	2604	6200	[34]
	South Africa	2015/2016	TP/7	38	SPE-GC-MS	143/477	NA	NA	17600	[133]
	Spain	2005/2006	NA/7	21	SPE-LC-MS/MS	8/NA	100	1370	9890	[103]
	Spain	2006	GS/8	16	SPE-LC-MS/MS	NA	100	152.9	490.4	[120]
	Spain	2011/2012	NA/1	5	SPE-LC-MS/MS	0.7/2.4	60	397	750	[64]
	Spain	2012	GS/22	22	SPE-UPLC-MS/MS	NA/20	82	830	16 482	[42]

Pharmaceutical	Country	Year	Sampling type/locations	Number of samples	Method	LOD/LOQ (ng L^{-1})	Frequency (highest value) (%)	Average (highest value) (ng L^{-1})	Maximum (highest value) (ng L^{-1})	Reference
	Spain	NA	NA/10	10	SPE-LC-MS/MS	8/42	NA	60	150	[14]
	Spain	NA	NA/7	7	SPE-LC-MS/MS	NA	100	1368	4350	[121]
	Spain	NA	5	5	SPE-UPLC-MS/MS	2.0/6.5	80	113	193	[94]
	Spain/Belgium/Germany	NA	TP/NA	16	SPE-GC/LC-MS/MS	12/42	NA	NA	152	[62]
	Sweden	2014	TP/8	8	SPE-UPLC-MS/MS	NA/2.0	0	ND	ND	[124]
	UK	2007	GS/2	40	SPE-LC-MS/MS	NA	93	24	74	[35]
	UK	NA	NA	NA	NA	NA	NA	NA	5044	[36]
	USA	1999/2000	CS/NA	84	SPE-UPLC-MS/MS	18/NA	9.5	200	1000	[116]
	USA	2001	NA/74	74	SPE-LC-MS/MS	18/NA	1.4	NA	270	[104]
	USA	2008/2009	GS/7	42	SPE-LC-MS/MS	4.7/14.0	0	ND	ND	[106]
	USA	2012/2013	GS/4	28	SPE-UPLC-MS/MS	0.085/NA	4	NA	4.98	[87]
	USA	2014/2015	GS/2	12	SPE-UPLC-MS/MS	NA	NA	0.14	1.08	[74]
	USA	NA	NA	NA	NA	NA	NA	NA	1000	[117]
							52.7(100)	387.9(3712.1)	3265.3(36 788)	
Naproxen (NAP)	America	NA	NA	NA	NA	NA	NA	NA	555	[75]
	Brazil	1996	TP/17	17	SPE-GC-MS	10/NA	NA	20	210	[38]
	Canada	2005	GS/2	8	SPE-GC-MS	0.5/NA	22.2	145	271.4	[83]

Pharmaceutical	Country	Year	Sampling type/locations	Number of samples	Method	LOD/LOQ (ng L^{-1})	Frequency (highest value) (%)	Average (highest value) (ng L^{-1})	Maximum (highest value) (ng L^{-1})	Reference
	Canada	2006/2007	GS/17	125	SPE-LC-MS/MS	NA/2	21	1	199	[113]
	Canada	2010	GS/9	9	SPE-GC-MS/MS	41/NA	NA	NA	45	[88]
	China	2007	GS/14	14	SPE-GC-MS	1.3/4.2	29	60.2	118	[125]
	China	2013	GS/19	57	SPE-LC-MS/MS	0.06/NA	74	0.5	10.8	[126]
	China	NA	NA	NA	NA	NA	NA	NA	328	[96]
	Europe	NA	NA	NA	NA	NA	NA	NA	80.5	[75]
	France	2007/2008	GS/8	24	SPE-LC-MS/MS	NA/4	46	NA	6.4	[92]
	Germany	2007	TP/2	36	SPE-LC-MS/MS	NA/16	NA	34	830	[123]
	Greece	NA	NA	NA	NA	NA	NA	NA	322	[33]
	International	NA	NA	NA	NA	NA	69	98	19 600	[100]
	Italy	2011	CS/2	6	SPE-LC-MS/MS	NA	NA	NA	10	[71]
	Korea	2004/2005	GS/3	8	SPE-LC-MS/MS	NA	75	11	18	[79]
	Korea	2007	GS/5	5	SPE-LC-MS/MS	NA	NA	NA	12	[27]
	Mexico	2015/2016	TP/4	16	SPE- LC-MS/MS	0.9/3.1	100	2537.8	4880	[41]
	NA	NA	NA	NA	NA	NA	NA	NA	390	[85]
	Portugal	2009	GS/6	12	SPE-LC-MS/MS	14/43	25	34.8	136	[90]
	Portugal	2013	GS/NA	NA	SPE-UPLC-MS/MS	NA/NA	NA	NA	6	[112]
	Portugal	2013/2014	GS/5	55	SPE-UHPLC-MS/MS	0.12/0.36	45.5	0.06	260	[20]

**Pharmaceutical**	**Country**	**Year**	**Sampling type/locations**	**Number of samples**	**Method**	**LOD/LOQ (ng L^{-1})**	**Frequency (highest value) (%)**	**Average (highest value) (ng L^{-1})**	**Maximum (highest value) (ng L^{-1})**	**Reference**
	South Africa	2015/2016	TP/7	38	SPE-GC-MS	75/248	NA	NA	59300	[133]
	Spain	2005/2006	NA/7	21	SPE-LC-MS/MS	30/NA	55	530	1810	[103]
	Spain	2006	GS/8	16	SPE-LC-MS/MS	NA	100	42.6	105.5	[120]
	Spain	2010	NA/10	10	TF-LC-MS/MS	19.0/6.4	NA	NA	63.5	[93]
	Spain	2012	GS/22	22	SPE-UPLC-MS/MS	NA/6	50	278	1797	[42]
	Spain	NA	NA/10	10	SPE-LC-MS/MS	7/20	NA	33	50	[14]
	Spain	NA	NA/7	7	SPE-LC-MS/MS	NA	100	228	687	[121]
	Spain/Belgium/Germany	NA	TP/NA	16	SPE-GC/LC-MS/MS	26/75	NA	NA	70	[62]
	Sweden	2014	TP/8	8	SPE-UPLC-MS/MS	NA/1.0	0	ND	ND	[124]
	UK	2007	GS/2	40	SPE-LC-MS/MS	NA	98	32	146	[35]
	UK	NA	NA	NA	NA	NA	NA	NA	146	[36]
	USA	2006/2007	GS/19	32	SPE-GC/LC-MS/MS	0.50/NA	58	0.9	32	[127]
	USA	2008/2009	GS/7	42	SPE-LC-MS/MS	1.0/2.9	23.8	5.0	30.8	[106]
	USA	2014/2015	GS/2	12	SPE-UPLC-MS/MS	NA	NA	1.82	6.10	[74]
	USA	NA	NA	NA	NA	NA	NA	NA	80	[117]
							55.1(100)	204.7(2537.8)	2572.6(59 300)	

Pharmaceutical	Country	Year	Sampling type/locations	Number of samples	Method	LOD/LOQ (ng L^{-1})	Frequency (highest value) (%)	Average (highest value) (ng L^{-1})	Maximum (highest value) (ng L^{-1})	Reference
Paracetamol (PARA)	Canada	2006/2007	GS/17	125	SPE-LC-MS/MS	NA/2	11	0.1	298	[113]
	China	2012/2013	GS/20	20	SPE-LC-MS/MS	NA/NA	5	4.2	84.8	[108]
	China	2013	GS/19	57	SPE-LC-MS/MS	0.10/NA	96	14.0	197	[126]
	Europe	NA	NA	NA	NA	NA	NA	NA	40	[75]
	France	2007/2008	GS/8	24	SPE-LC-MS/MS	NA/2	100	NA	71.0	[92]
	International	NA	NA	NA	NA	NA	51.6	148	15 700	[100]
	Japan	2006/2010	GS/8	27	SPE-LC-MS/MS	NA/2.8	25.9	19.5	29.0	[118]
	Kenya	2012/2013	GS/3	14	SPE-LC-MS	NA/NA	71.42	24 449	106 970	[26]
	Korea	2004/2005	GS/3	8	SPE-LC-MS/MS	NA	75	33	73	[79]
	Korea	2007	GS/5	5	SPE-LC-MS/MS	NA	NA	NA	80	[27]
	Portugal	2009	GS/6	12	SPE-LC-MS/MS	6/20	50	336	925	[90]
	Portugal	2011	GS/7	7	SPE-LC-MS/MS	42/141	29	210	250	[134]
	Portugal	2013	GS/NA	NA	SPE-UPLC-MS/MS	NA/NA	NA	NA	30	[112]
	Portugal	2016	GS/31	31	UHPLC-TOF-MS	NA/NA	80.6	1.8	10.6	[91]
	Scotland	2005/2006	GS/4	40	SPE-LC-MS/MS	3.9/13.1	15	7.0	9.1	[80]
	Serbia	NA	GS/14	14	SPE-LC-MS/MS	0.27/0.90	29	292	610	[72]
	South Africa	2013	GS/12	12	SPE-LC-MS/MS	2.7/91	100	1360	1780	[34]

Pharmaceutical	Country	Year	Sampling type/locations	Number of samples	Method	LOD/LOQ (ng L^{-1})	Frequency (highest value) (%)	Average (highest value) (ng L^{-1})	Maximum (highest value) (ng L^{-1})	Reference
	Spain	2005/2006	NA/7	21	SPE-LC-MS/MS	17/NA	90	420	2420	[103]
	Spain	2006	GS/8	16	SPE-LC-MS/MS	NA	100	34.8	96.5	[120]
	Spain	2010	NA/10	10	TF-LC-MS/MS	14.5/48.3	NA	NA	156	[93]
	Spain	NA	NA/10	10	SPE-LC-MS/MS	17/40	NA	42	250	[14]
	Spain	NA	NA/7	7	SPE-LC-MS/MS	NA	100	380	830	[121]
	Sweden	2014	TP/8	8	SPE-UPLC-MS/MS	NA/0.04	100	40.1	170	[124]
	Taiwan	2007/2008	GS/15	15	SPE-LC-MS/MS	2.0/NA	97	NA	15 700	[122]
	UK	2007	GS/2	40	SPE-LC-MS/MS	NA	85	483	1534	[35]
	UK	NA	NA	NA	NA	NA	NA	NA	2382	[43]
	USA	1999/2000	CS/NA	84	SPE-UPLC-MS/MS	9/NA	23.8	110	10 000	[116]
	USA	2001	NA/74	74	SPE-LC-MS/MS	9/NA	8.1	NA	160	[104]
	USA	2008/2009	GS/7	42	SPE-LC-MS/MS	2.5/7.5	23.8	20.7	72.8	[106]
	USA	2014/2015	GS/2	12	SPE-UPLC-MS/MS	NA	NA	ND	ND	[74]
	USA	NA	NA	NA	NA	NA	NA	NA	10 000	[117]
p-aminophenol	Portugal	2011	GS/7	7	SPE-LC-MS/MS	67/225	57	950	1630	[134]
							59.4(100)	1291.1(24 448.6)	5513.8(106 970)	
Total							53.8(100)	490.9(24 448.6)	3138.5(106 970)	
Hormones										
Estrone	Austria	NA	NA	NA	NA	NA	NA	NA	4.6	[75]

Pharmaceutical	Country	Year	Sampling type/locations	Number of samples	Method	LOD/LOQ (ng L-1)	Frequency (highest value) (%)	Average (highest value) (ng L-1)	Maximum (highest value) (ng L-1)	Reference
(E1)	Canada	2006/2007	GS/17	125	SPE-LC-MS/MS	NA	0	ND	ND	[113]
	China	2007	GS/14	14	SPE-GC-MS	0.2/0.5	93	14.9	75.0	[125]
	China	NA	NA/1	5	SPE-GC-MS	18/62	NA	180	NA	[135]
	France	2007	NA/2	2	SPE-LC-MS/MS	0.02/NA	50	0.3	0.3	[136]
	France	2007/2008	GS/8	24	SPE-LC-MS/MS	NA/0.04	33	NA	2	[92]
	France	NA	NA	NA	NA	NA	NA	NA	0.3	[75]
	Germany	2002	NA/1	5	SPE-LC-MS/MS	NA/0.1	100	0.86	NA	[65]
	Italy	2002	FP/1	7	SPE-LC-MS/MS	0.1/NA	100	8	15	[67]
	Japan	2001	GS/8	8	SPE-GC-MS/MS	0.1	100	2.1	6.6	[89]
	Korea	2004/2005	GS/3	8	SPE-LC-MS/MS	NA	38	3.6	5	[79]
	Korea	NA	NA	NA	NA	NA	NA	NA	69.1	[33]
	Korea	NA	NA	NA	NA	NA	NA	NA	5	[75]
	Luxembourg	2007/2008	TP/1	24	SPE-LC-MS/MS	0.3/1.0	NA	NA	27	[60]
	Spain	2006	GS/8	16	SPE-LC-MS/MS	NA	93	NA	5.8	[120]
	Taiwan	2007/2008	GS/15	15	SPE-LC-MS/MS	25/NA	8	NA	61	[122]
	USA	1999/2000	CS/NA	70	SPE-UPLC-MS/MS	5/NA	7.1	27	112	[116]
	USA	2006/2007	GS/19	32	SPE-GC/LC-MS/MS	0.20/NA	79	0.3	0.9	[127]

Pharmaceutical	Country	Year	Sampling type/locations	Number of samples	Method	LOD/LOQ (ng L^{-1})	Frequency (highest value) (%)	Average (highest value) (ng L^{-1})	Maximum (highest value) (ng L^{-1})	Reference
	USA	2012/2013	GS/4	28	SPE-UPLC-MS/MS	6.48/NA	0	ND	ND	[87]
							53.9(100)	21.6(180.0)	22.9(112.0)	
17β-estradiol (E2)	Austria	NA	NA	NA	NA	NA	NA	NA	1.2	[75]
	Canada	2006/2007	GS/17	125	SPE-LC-MS/MS	NA	0	ND	ND	[113]
	China	2007	GS/14	14	SPE-GC-MS	0.3/1.0	71	2.1	7.5	[125]
	China	NA	NA/1	5	SPE-GC-MS	9/32	NA	100	NA	[135]
	France	2007/2008	GS/8	24	SPE-LC-MS/MS	NA/0.06	0	ND	ND	[92]
	Italy	2002	FP/1	7	SPE-LC-MS/MS	0.2/NA	100	4	6	[67]
	Japan	2001	GS/8	8	SPE-GC-MS/MS	0.2	38	0.8	1.0	[89]
	Korea	NA	NA	NA	NA	NA	NA	NA	10.1	[33]
	Luxembourg	2007/2008	TP/1	24	SPE-LC-MS/MS	1.0/3.0	NA	NA	35	[60]
	South Africa	2014	GS/4	80	SPE-HPLC-DAD	825.5/2476.5	NA	NA	15 700	[137]
	Spain	2006	GS/8	16	SPE-LC-MS/MS	NA	0	ND	ND	[120]
	Taiwan	2007/2008	GS/15	15	SPE-LC-MS/MS	25/NA	10	NA	119	[122]
	USA	1999/2000	CS/NA	70	SPE-UPLC-MS/MS	5/NA	10	9	93	[116]
	USA	2006/2007	GS/19	32	SPE-GC/LC-MS/MS	0.50/NA	5	17	17	[127]
	USA	2008/2009	GS/7	42	SPE-LC-MS/MS	1.3/3.8	7.1	1.3	1.7	[106]

Pharmaceutical	Country	Year	Sampling type/locations	Number of samples	Method	LOD/LOQ (ng L^{-1})	Frequency (highest value) (%)	Average (highest value) (ng L^{-1})	Maximum (highest value) (ng L^{-1})	Reference
	USA	2012/2013	GS/4	28	SPE-UPLC-MS/MS	14.8/NA	0	ND	ND	[87]
	USA	2014/2015	GS/2	12	SPE-UPLC-MS/MS	NA	NA	0.45	5.32	[74]
							21.9(100)	11.2(100.0)	999.8(15 700)	
17α-estradiol (α-E2)	Canada	2006/2007	GS/17	125	SPE-LC-MS/MS	NA	0	ND	ND	[113]
	France	2007/2008	GS/8	24	SPE-LC-MS/MS	NA/0.02	0	ND	ND	[92]
	Japan	2001	GS/8	8	SPE-GC-MS/MS	0.2	0	ND	ND	[89]
	USA	1999/2000	CS/NA	70	SPE-UPLC-MS/MS	5/NA	5.7	30	74	[116]
							1.4(5.7)	7.5(30.0)	18.5(74.0)	
17α-ethinylestradiol (EE2)	Austria	NA	NA	NA	NA	NA	NA	NA	0.33	[75]
	Canada	2006/2007	GS/17	125	SPE-LC-MS/MS	NA	0	ND	ND	[113]
	France	2007/2008	GS/8	24	SPE-LC-MS/MS	NA/0.4	0	ND	ND	[92]
	Italy	2002	FP/1	7	SPE-LC-MS/MS	0.4/NA	NA	1	3	[67]
	Korea	2004/2005	GS/3	8	SPE-LC-MS/MS	NA	0	ND	ND	[79]
	Korea	NA	NA	NA	NA	NA	NA	NA	1.9	[33]
	Luxembourg	2007/2008	TP/1	24	SPE-LC-MS/MS	2.0/6.0	0	ND	ND	[60]
	Spain	2006	GS/8	16	SPE-LC-MS/MS	NA	0	ND	ND	[120]
	Spain	NA	NA/5	5	SPE-UPLC-MS/MS	2.4/8.0	0	ND	ND	[94]
	USA	1999/2000	CS/NA	70	SPE-UPLC-MS/MS	5/NA	15.7	73	831	[116]

**Pharmaceutical**	**Country**	**Year**	**Sampling type/locations**	**Number of samples**	**Method**	**LOD/LOQ (ng L^{-1})**	**Frequency (highest value) (%)**	**Average (highest value) (ng L^{-1})**	**Maximum (highest value) (ng L^{-1})**	**Reference**
	USA	2006/2007	GS/19	32	SPE-GC/LC-MS/MS	1.0/NA	5	1.4	1.4	[127]
	USA	2012/2013	GS/4	28	SPE-UPLC-MS/MS	8.48/NA	0	ND	ND	[87]
	USA	2014/2015	GS/2	12	SPE-UPLC-MS/MS	NA	NA	ND	ND	[74]
							2.3(15.7)	6.9(73.0)	64.4(831.0)	
Total							26.2(100)	12.6(180.0)	346.0(15 700)	

CS - Composite sampling
ESI- Electrospray ionization
FP - Flow proportional sampling
FD - Fluorescence detection
GC - Gas chromatography
GS - Grab sampling
HPLC - High performance liquid chromatography
LC - Liquid chromatography
LOQ - Limit of quantification
MS - Mass spectrometry
NA - Not available
ND - Not detected
SPE - Solid phase extraction
TF - TurboFlow
TOF - Time-of-flight
TP - Time proportional sampling
UPLC - Ultra performance liquid chromatography
UHPLC - Ultra high performance liquid chromatography

Table S5. Occurrence of pharmaceuticals in seawaters (SeaW), groundwaters (GWs), drinking waters (DWs) and mineral waters (DWs).

Pharmaceutical	Sample	Country	Year	Sampling type/locations	Number of samples	Method	LOD/LOQ (ng L^{-1})	Frequency (highest value) (%)	Average (highest value) (ng L^{-1})	Maximum (highest value) (ng L^{-1})	Reference
Anxiolytics											
Alprazolam (ALP)	DW	China	2014	GS/5	5	SPE-LC–MS/MS	NA/0.1	100	2.4	2.6	[4]
Lorazepam (LOR)	GW	Serbia	NA	GS/10	10	SPE-LC-MS/MS	0.55/1.82	0	ND	ND	[72]
	GW	Spain	2010/2011	GS/31	31	SPE-LC-MS/MS	NA	23	5.1	54	[138]
	**GW**							11.5(23)	2.6(5.1)	27(54)	
	DW	France	2007/2008	GS/8	24	SPE-LC-MS/MS	NA/12	4	NA	<LOQ	[92]
	DW	China	2014	GS/5	5	SPE-LC–MS/MS	NA/4.0	0	ND	ND	[4]
	**DW**							2.0(4.0)	0	0	
Total								25.4(100)	1.9(5.1)	11.3(54.0)	
Antibiotics											
Azithromycin (AZI)	SeaW	China	2009	GS/27	27	SPE-LC-MS/MS	NA/0.28	22	0.14	1.2	[95]
	GW	Serbia	NA	GS/10	10	SPE-LC-MS/MS	2.58/8.59	20	83	140	[72]
	GW	Spain	2010/2011	GS/31	31	SPE-LC-MS/MS	NA	93	96.3	1620	[138]
	GW	USA	2014/2015	GS/6	36	SPE-UPLC-MS/MS	NA	NA	12.21	131.43	[74]
	**GW**							56.5(93)	63.8(96.3)	630.5(1620)	
								45.0(93.0)	47.9(96.3)	473.2(1620.0)	
Clarithromycin (CLA)	SeaW	China	2009	GS/27	27	SPE-LC-MS/MS	NA/0.27	37	0.19	0.82	[95]

Pharmaceutical	Sample	Country	Year	Sampling type/locations	Number of samples	Method	LOD/LOQ (ng L-1)	Frequency (highest value) (%)	Average (highest value) (ng L-1)	Maximum (highest value) (ng L-1)	Reference
	GW	Portugal	2013	GS/NA	NA	SPE-UPLC-MS/MS	NA/NA	NA	NA	3	[112]
	GW	Spain	2010/2011	GS/31	31	SPE-LC-MS/MS	NA	100	5.2	20.5	[138]
	**GW**							100	5.2	11.8(20.5)	
	DW	Portugal	2013	GS/15	NA	SPE-UPLC-MS/MS	NA/NA	8	2	5	[112]
								48.3(100)	2.5(5.2)	7.33(20.5)	
Ciprofloxacin (CIP)	SeaW	China	2009	GS/27	27	SPE-LC-MS/MS	NA/4.8	93	31	66	[95]
	SeaW	Spain	2011	GS/4	48	SPE-LC-MS/MS	1.0/3.4	60	72	303.6	[139]
	**SeaW**							76.5(93)	51.5(72)	184.8(303.6)	
	GW	America	NA	NA	NA	NA	NA	NA	NA	0.28	[75]
	GW	Asia	NA	NA	NA	NA	NA	NA	NA	40	[75]
	GW	India (near industry)	2008	GS/6	NA	SPE-LC-MS	NA/10	NA	NA	14 000	[77]
	GW	Spain	2010/2011	GS/31	31	SPE-LC-MS/MS	NA	91	38.9	443	[138]
	GW	USA	2000	NA/47	47	SPE-LC-MS/MS	20/NA	0	ND	ND	[140]
	**GW**							45.5(91)	19.5(38.9)	2896.7(14 000)	
	DW	Canada	2006/2007	GS/17	123	SPE-LC-MS/MS	NA	0	ND	ND	[113]
								48.8(93.0)	28.4(72.0)	1856.6(14 000)	
Erythromycin (ERY)	SeaW	China	2009	GS/27	27	SPE-LC-MS/MS	NA/0.26	100	2.6	8.5	[95]
	GW	Serbia	NA	GS/10	10	SPE-LC-MS/MS	1.65/5.48	0	ND	ND	[72]
	GW	Spain	2010/2011	GS/31	31	SPE-LC-MS/MS	NA	33	9.6	41.3	[138]

Pharmaceutical	Sample	Country	Year	Sampling type/locations	Number of samples	Method	LOD/LOQ (ng L^{-1})	Frequency (highest value) (%)	Average (highest value) (ng L^{-1})	Maximum (highest value) (ng L^{-1})	Reference
	GW	USA	2000	NA/47	47	SPE-LC-MS/MS	50/NA	0	ND	ND	[140]
	GW	USA	2014/2015	GS/6	36	SPE-UPLC-MS/MS	NA	NA	0.04	1.25	[74]
	**GW**							11(33)	2.4(9.6)	10.6(41.3)	
	DW	Canada	2006/2007	GS/17	123	SPE-LC-MS/MS	NA/10	3	0.3	13	[113]
	DW	Japan	2006/2010	GS/8	23	SPE-LC-MS/MS	NA/10.0	0	ND	ND	[118]
	DW	Netherlands	2009	GS/2	15	SPE-LC-MS/MS	NA	0	ND	ND	[119]
	**DW**							1(3)	0.1(0.3)	4.3(13)	
								19.4(100)	1.6(9.6)	8.0(41.3)	
Total								36.7(100)	17.7(96.3)	701.6(14 000)	
Lipid regulators											
Bezafibrate (BEZ)	GW	Canada	NA	NA	NA	NA	NA	NA	NA	12	[117]
	GW	Germany	NA	NA	NA	NA	NA	NA	NA	112	[33]
	GW	Spain	2010/2011	GS/31	31	SPE-LC-MS/MS	NA	51	2.2	25.8	[138]
	**GW**							51	2.2	49.9(112)	
	MinW	Spain	2012	GS/11	11	SPE-UPLC-MS/MS	NA/0.2	18	1	10	[42]
	DW	Canada	2006/2007	GS/17	123	SPE-LC-MS/MS	NA/0.5	2	NA	1	[113]
	DW	France	2007/2008	GS/8	24	SPE-LC-MS/MS	NA/1	42	NA	2.2	[92]
	DW	Germany	NA	NA	NA	NA	NA	NA	NA	27	[117]
	DW	Japan	2006/2010	GS/8	23	SPE-LC-MS/MS	NA/10.0	0	ND	ND	[118]
	DW	Netherlands	2009	GS/2	15	SPE-LC-MS/MS	NA	0	ND	ND	[119]

Pharmaceutical	Sample	Country	Year	Sampling type/locations	Number of samples	Method	LOD/LOQ (ng L^{-1})	Frequency (highest value) (%)	Average (highest value) (ng L^{-1})	Maximum (highest value) (ng L^{-1})	Reference
	DW	Spain	2012	GS/5	8	SPE-UPLC-MS/MS	NA/0.2	0	ND	ND	[42]
	**DW**							8.8(42)	0	5.0(27)	
								16.1(51.0)	0.6(2.2)	19.0(112.0)	
Gemfibrozil (GEM)	SeaW	Ireland	2011/2012	GS/2	12	SPE-LC-MS/MS	38/NA	50	258	400	[84]
	SeaW	Sweden	NA	GS/13	13	bag SPE-LC-MS/MS	6/20	0	ND	ND	[141]
	**SeaW**							25(50)	129(258)	200(400)	
	GW	Canada	NA	NA	NA	NA	NA	NA	NA	430	[117]
	GW	Portugal	2013	GS/NA	NA	SPE-UPLC-MS/MS	NA/NA	0	ND	ND	[112]
	GW	Spain	2010/2011	GS/31	31	SPE-LC-MS/MS	NA	81	69.9	751	[138]
	GW	Spain	NA	NA	NA	NA	NA	NA	NA	574	[33]
	GW	USA	2000	NA/47	47	SPE-LC-MS/MS	15/NA	0	ND	ND	[140]
	GW	USA	2014/2015	GS/6	36	SPE-UPLC-MS/MS	NA	NA	0.15	2.20	[74]
	**GW**							27.0(81)	17.5(69.9)	292.9(751.0)	
	MinW	Spain	2012	GS/11	11	SPE-UPLC-MS/MS	NA/0.3	82	8	20	[42]
	DW	Canada	2006/2007	GS/17	123	SPE-LC-MS/MS	NA/1	15	0.5	4	[113]
	DW	Canada	NA	NA	NA	NA	NA	NA	NA	70	[117]
	DW	Portugal	2013	GS/15	NA	SPE-UPLC-MS/MS	NA/NA	NA	NA	18	[112]
	DW	Spain	2012	GS/5	8	SPE-UPLC-MS/MS	NA/0.3	38	2	7	[42]
	DW	USA	2006/2007	GS/19	33	SPE-GC/LC-MS/MS	0.25/NA	33	0.46	2.1	[127]
	**DW**							28.7(38)	1.0(2.0)	20.2(70.0)	

Pharmaceutical	Sample	Country	Year	Sampling type/locations	Number of samples	Method	LOD/LOQ (ng L^{-1})	Frequency (highest value) (%)	Average (highest value) (ng L^{-1})	Maximum (highest value) (ng L^{-1})	Reference
								33.2(82.0)	33.9(258.0)	162.7(751.0)	
Total								25.8(82.0)	22.8(258.0)	102.8(751.0)	
Antiepileptic											
Carbamazepine (CAR)	SeaW	Ireland	2011/2012	GS/2	12	SPE-LC-MS/MS	4/NA	100	678	1410	[84]
	SeaW	Spain	2011	GS/4	48	SPE-LC-MS/MS	0.9/2.9	0	ND	ND	[139]
	SeaW	Sweden	NA	GS/13	13	bag SPE-LC-MS/MS	1/3	100	NA	26.3	[141]
	**SeaW**							66.7(100)	339.0(678)	478.8(1410)	
	GW	America	NA	NA	NA	NA	NA	NA	NA	420	[75]
	GW	Europe	NA	NA	NA	NA	NA	NA	NA	3600	[75]
	GW	Germany	2000	NA/105	105	SPE-GC/LC-MS/MS	32/NA	12.4	NA	900	[142]
	GW	Kenya	2012/2013	GS/3	3	SPE-LC-MS	NA/NA	66.6	23.3	40.0	[26]
	GW	NA	NA	NA	NA	NA	NA	NA	NA	465	[117]
	GW	Portugal	2013	GS/NA	NA	SPE-UPLC-MS/MS	NA/NA	0	NA	3	[112]
	GW	Serbia	NA	GS/10	10	SPE-LC-MS/MS	0.27/0.90	60	14.5	23	[72]
	GW	Spain	2010/2011	GS/31	31	SPE-LC-MS/MS	NA	97	44.3	136	[138]
	GW	USA	2004-2010	NA/1231	1231	NA	30/NA	1.46	40	420	[143]
	GW	USA	2014/2015	GS/6	36	SPE-UPLC-MS/MS	NA	NA	5.10	15.90	[74]
	GW	USA	NA	NA	NA	NA	NA	NA	NA	420	[33]
	**GW**							39.6(97)	25.4(44.3)	585.7(3600)	
	DW	Canada	2006/2007	GS/17	123	SPE-LC-MS/MS	NA/1	25	0.21	601	[113]
	DW	China	2014	GS/5	5	SPE-LC–MS/MS	NA/2.3	100	NA	2.5	[4]

**Pharmaceutical**	**Sample**	**Country**	**Year**	**Sampling type/locations**	**Number of samples**	**Method**	**LOD/LOQ (ng L^{-1})**	**Frequency (highest value) (%)**	**Average (highest value) (ng L^{-1})**	**Maximum (highest value) (ng L^{-1})**	**Reference**
	DW	France	2007/2008	GS/8	24	SPE-LC-MS/MS	NA/1	83	NA	32.0	[92]
	DW	Italy	NA	GS/21	21	SPE--LC–MS/MS	NA/0.18	14.3	7.6	10.3	[144]
	DW	Japan	2006/2010	GS/8	23	SPE-LC-MS/MS	NA/0.4	13.0	13.6	24.8	[118]
	DW	Netherlands	2007	NA	2	NA	NA	NA	NA	30	[130]
	DW	Netherlands	2009	GS/2	15	SPE-LC-MS/MS	NA	0	ND	ND	[119]
	DW	Portugal	2013	GS/15	NA	SPE-UPLC-MS/MS	NA/NA	96	1.9	14	[112]
	DW	USA	2006/2007	GS/19	33	SPE-GC/LC-MS/MS	0.25/NA	42	6.4	18	[127]
	DW	USA	2012/2013	GS/4	27	SPE-UPLC-MS/MS	0.03/NA	0	ND	ND	[87]
	DW	USA	NA	NA	NA	NA	NA	NA	NA	258	[117]
	**DW**							41.5(100)	4.2(13.6)	90.1(601.0)	
								45.0(100)	59.6(678.0)	354.8(3600.0)	
Selective serotonin reuptake inhibitors (SSRIs)											
Citalopram (CIT)	GW	India (near industry)	2008	GS/6	NA	SPE-LC-MS	NA/10	NA	NA	1400	[77]
Fluoxetine (FLU)	GW	Spain	2010/2011	GS/31	31	SPE-LC-MS/MS	NA	18	1.8	21	[138]
	GW	USA	2000	NA/47	47	SPE-LC-MS/MS	18/NA	4.3	NA	56	[140]
	GW	USA	2014/2015	GS/6	36	SPE-UPLC-MS/MS	NA	NA	19.84	410.77	[74]
	**GW**							11.2(18.0)	10.8(19.8)	162.6(410.8)	
	DW	China	2014	GS/5	5	SPE-LC–MS/MS	NA/0.2	0	ND	0.2	[4]

Pharmaceutical	Sample	Country	Year	Sampling type/locations	Number of samples	Method	LOD/LOQ (ng L-1)	Frequency (highest value) (%)	Average (highest value) (ng L-1)	Maximum (highest value) (ng L-1)	Reference
	DW	France	2007/2008	GS/8	24	SPE-LC-MS/MS	NA/85	0	ND	ND	[92]
	DW	USA	2006/2007	GS/19	33	SPE-GC/LC-MS/MS	0.50/NA	9	0.68	0.82	[127]
	**DW**							3.0(9.0)	0.2(0.7)	0.3(0.8)	
								6.26(18)	4.5(19.8)	81.5(410.8)	
Norfluoxetine (Nor-FLU)	DW	France	2007/2008	GS/8	24	SPE-LC-MS/MS	NA/32	0	ND	ND	[92]
	DW	USA	2006/2007	GS/19	33	0	NA	NA	0.77	0.77	[127]
	**DW**							0	0.4(0.8)	0.4(0.8)	
								0(0)	0.4(0.8)	0.4(0.8)	
Paroxetine (PAR)	GW	Spain	2010/2011	GS/31	31	SPE-LC-MS/MS	NA	20	2.4	30.2	[138]
	GW	USA	2014/2015	GS/6	36	SPE-UPLC-MS/MS	NA	NA	1.4	5.88	[74]
	**GW**							20(20)	1.9(2.4)	18.0(30.2)	
	DW	Japan	2006/2010	GS/8	23	SPE-LC-MS/MS	NA/8.0	0	ND	ND	[118]
								10.0(20.0)	1.3(2.4)	12.0(30.2)	
Total								6.4(20)	2.7(19.8)	160.5(1400.0)	
Anti-inflammatories											
Diclofenac (DIC)	SeaW	Ireland	2011/2012	GS/2	12	SPE-LC-MS/MS	22/NA	92	315	460	[84]
	SeaW	Spain	2011	GS/4	48	SPE-LC-MS/MS	1.4/4.6	18.8	86	343.6	[139]
	SeaW	Sweden	NA	GS/13	13	bag SPE-LC-MS/MS	4/13	0	ND	ND	[141]
	**SeaW**							36.9(92.0)	133.7(315.0)	267.9(460.0)	
	GW	Europe	NA	NA	NA	NA	NA	NA	NA	24	[75]
	GW	Germany	2000	NA/105	105	SPE-GC/LC-MS/MS	29/NA	3.8	NA	590	[142]

Pharmaceutical	Sample	Country	Year	Sampling type/locations	Number of samples	Method	LOD/LOQ (ng L^{-1})	Frequency (highest value) (%)	Average (highest value) (ng L^{-1})	Maximum (highest value) (ng L^{-1})	Reference
	GW	Germany	NA	NA	NA	NA	NA	NA	NA	3050	[33]
	GW	Portugal	2013	GS/NA	NA	SPE-UPLC-MS/MS	NA/NA	0	ND	ND	[112]
	GW	Serbia	NA	GS/10	10	SPE-LC-MS/MS	0.15/0.49	0	ND	ND	[72]
	GW	Spain	2010/2011	GS/31	31	SPE-LC-MS/MS	NA	65	75.3	380	[138]
	**GW**							17.2(65.0)	25.1(75.3)	674.0(3050.0)	
	MinW	Spain	2012	GS/11	11	SPE-UPLC-MS/MS	NA/1.0	55	25	87	[42]
	DW	Canada	2006/2007	GS/17	123	SPE-LC-MS/MS	NA	1	NA	NA	[113]
	DW	France	2007/2008	GS/8	24	SPE-LC-MS/MS	NA/1	29	NA	1.0	[92]
	DW	Germany	NA	NA	NA	NA	NA	NA	NA	35	[117]
	DW	Japan	2006/2010	GS/8	23	SPE-LC-MS/MS	NA/2.5	4.3	15.8	15.8	[118]
	DW	Portugal	2013	GS/15	NA	SPE-UPLC-MS/MS	NA/NA	NA	NA	11	[112]
	DW	Spain	2012	GS/5	8	SPE-UPLC-MS/MS	NA/1.0	38	18	60	[42]
	DW	USA	2006/2007	GS/19	33	SPE-GC/LC-MS/MS	0.25/NA	0	ND	ND	[127]
	**DW**							14.5(38.0)	11.3(18.0)	20.5(60.0)	
								23.6(92.0)	53.5(315.0)	316.1(3050.0)	
4-hydroxydiclofenac (4-OH-DIC)	GW	Spain	2010/2011	GS/31	31	SPE-LC-MS/MS	NA	26	15.3	147	[138]
Ibuprofen (IBU)	SeaW	Sweden	NA	GS/13	13	bag SPE-LC-MS/MS	7/23	0	ND	ND	[141]
	GW	America	NA	NA	NA	NA	NA	NA	NA	3.97	[75]

Pharmaceutical	Sample	Country	Year	Sampling type/locations	Number of samples	Method	LOD/LOQ (ng L^{-1})	Frequency (highest value) (%)	Average (highest value) (ng L^{-1})	Maximum (highest value) (ng L^{-1})	Reference
	GW	Canada	NA	NA	NA	NA	NA	NA	NA	2150	[117]
	GW	Europe	NA	NA	NA	NA	NA	NA	NA	395	[33,75]
	GW	Portugal	2013	GS/NA	NA	SPE-UPLC-MS/MS	NA/NA	0	ND	ND	[112]
	GW	Serbia	NA	GS/10	10	SPE-LC-MS/MS	3.28/10.93	0	ND	ND	[72]
	GW	Spain	2010/2011	GS/31	31	SPE-LC-MS/MS	NA	66	87.1	988	[138]
	GW	USA	2000	NA/47	47	SPE-LC-MS/MS	18/NA	2.1	ND	3110	[140]
	GW	USA	2014/2015	GS/6	36	SPE-UPLC-MS/MS	NA	NA	0.07	2.35	[74]
	**GW**							17.0(66.0)	17.4(87.1)	831.2(3110.0)	
	MinW	Spain	2012	GS/11	11	SPE-UPLC-MS/MS	NA/5.0	73	12	46	[42]
	DW	Canada	2006/2007	GS/17	123	SPE-LC-MS/MS	NA/0.5	15	0.33	25	[113]
	DW	France	2007/2008	GS/8	24	SPE-LC-MS/MS	NA/7	38	NA	1.3	[92]
	DW	Japan	2006/2010	GS/8	23	SPE-LC-MS/MS	NA/1.1	8.7	5.0	6.0	[118]
	DW	Portugal	2013	GS/15	NA	SPE-UPLC-MS/MS	NA/NA	NA	NA	21	[112]
	DW	Spain	2012	GS/5	8	SPE-UPLC-MS/MS	NA/5.0	88	39	94	[42]
	DW	USA	2012/2013	GS/4	27	SPE-UPLC-MS/MS	0.85/NA	4	NA	1.16	[87]
	DW	USA	NA	NA	NA	NA	NA	NA	NA	1350	[117]
	**DW**							30.7(88.0)	14.8(39.0)	214.1(1350.0)	
								26.8(88.0)	14.4(87.1)	427.7(3110.0)	
Naproxen (NAP)	SeaW	Sweden	NA	GS/13	13	bag SPE-LC-MS/MS	12/40	0	ND	ND	[141]

Pharmaceutical	Sample	Country	Year	Sampling type/locations	Number of samples	Method	LOD/LOQ (ng L^{-1})	Frequency (highest value) (%)	Average (highest value) (ng L^{-1})	Maximum (highest value) (ng L^{-1})	Reference
	GW	Canada	NA	NA	NA	NA	NA	NA	NA	300	[117]
	GW	Portugal	2013	GS/NA	NA	SPE-UPLC-MS/MS	NA/NA	0	ND	ND	[112]
	GW	Spain	2010/2011	GS/31	31	SPE-LC-MS/MS	NA	13	0.1	5.6	[138]
	GW	Spain	NA	NA	NA	NA	NA	NA	NA	263	[33]
	GW	USA	2014/2015	GS/6	36	SPE-UPLC-MS/MS	NA	NA	1.93	13.15	[74]
	**GW**							6.5(13.0)	0.7(1.9)	116.4(300.0)	
	MinW	Spain	2012	GS/11	11	SPE-UPLC-MS/MS	NA/0.5	82	25	49	[42]
	DW	Canada	2006/2007	GS/17	123	SPE-LC-MS/MS	NA/2	0	ND	ND	[113]
	DW	France	2007/2008	GS/8	24	SPE-LC-MS/MS	NA/4	17	NA	0.5	[92]
	DW	Portugal	2013	GS/15	NA	SPE-UPLC-MS/MS	NA/NA	NA	NA	6	[112]
	DW	Spain	2012	GS/5	8	SPE-UPLC-MS/MS	NA/0.5	100	11	93	[42]
	DW	USA	2006/2007	GS/19	33	SPE-GC/LC-MS/MS	0.50/NA	0	ND	ND	[127]
	**DW**							29.3(100.0)	3.7(11.0)	19.9(93.0)	
								26.5(100)	4.8(25.0)	60.9(300.0)	
Paracetamol (PARA)	SeaW	Spain	2011	GS/4	48	SPE-LC-MS/MS	0.6/2	6	116	297	[139]
	GW	Europe	NA	NA	NA	NA	NA	NA	NA	1890	[75]
	GW	France	NA	NA	NA	NA	NA	NA	NA	211	[117]
	GW	Kenya	2012/2013	GS/3	3	SPE-LC-MS	NA/NA	100	20	30	[26]

Pharmaceutical	Sample	Country	Year	Sampling type/locations	Number of samples	Method	LOD/LOQ (ng L^{-1})	Frequency (highest value) (%)	Average (highest value) (ng L^{-1})	Maximum (highest value) (ng L^{-1})	Reference
	GW	Portugal	2013	GS/NA	NA	SPE-UPLC-MS/MS	NA/NA	NA	NA	3	[112]
	GW	Serbia	NA	GS/10	10	SPE-LC-MS/MS	0.27/0.90	0	ND	ND	[72]
	GW	Spain	2010/2011	GS/31	31	SPE-LC-MS/MS	NA	5	<LOQ	<LOQ	[138]
	GW	USA	2000	NA/47	47	SPE-LC-MS/MS	9/NA	6.4	NA	380	[140]
	GW	USA	2004-2010	NA/1231	1231	NA	60/NA	0.32	180	1890	[143]
	GW	USA	2014/2015	GS/6	36	SPE-UPLC-MS/MS	NA	NA	0.23	2.5	[74]
	**GW**							22.3(100)	40.0(180.0)	489.6(1890.0)	
	DW	Canada	2006/2007	GS/17	123	SPE-LC-MS/MS	NA/2	1	NA	17	[113]
	DW	France	2007/2008	GS/8	24	SPE-LC-MS/MS	NA/2	100	NA	45.0	[92]
	DW	France	NA	NA	NA	NA	NA	NA	NA	210	[117]
	DW	Japan	2006/2010	GS/8	23	SPE-LC-MS/MS	NA/2.8	0	ND	ND	[118]
	DW	Portugal	2013	GS/15	NA	SPE-UPLC-MS/MS	NA/NA	NA	NA	47	[112]
	**DW**							33.7(100)	0	63.8(210.0)	
								24.3(100)	45.2(180.0)	334.8(1890.0)	
Total								25.2(100)	29.1(315.0)	313.9(3110.0)	
Hormones											
Estrone (E1)	GW	Austria	NA	NA	NA	NA	NA	NA	NA	1.6	[75]
	GW	France	2007	NA/3	3	SPE-LC-MS/MS	0.02/NA	67	2.2	3.5	[136]
	GW	France	NA	NA	NA	NA	NA	NA	NA	3.5	[75]
	GW	USA	NA	NA	NA	NA	NA	NA	NA	79	[33]

Pharmaceutical	Sample	Country	Year	Sampling type/locations	Number of samples	Method	LOD/LOQ (ng L^{-1})	Frequency (highest value) (%)	Average (highest value) (ng L^{-1})	Maximum (highest value) (ng L^{-1})	Reference
	**GW**							67.0	2.2	21.9(79.0)	
	DW	Canada	2006/2007	GS/17	123	SPE-LC-MS/MS	NA	0	ND	ND	[113]
	DW	France	2007/2008	GS/8	24	SPE-LC-MS/MS	NA/0.04	4	NA	0.3	[92]
	DW	Germany	2002	NA/1	5	SPE-LC-MS/MS	NA/0.1	100	0.16	NA	[65]
	DW	USA	2006/2007	GS/19	33	SPE-GC/LC-MS/MS	0.20/NA	0	ND	ND	[127]
	DW	USA	2012/2013	GS/4	27	SPE-UPLC-MS/MS	6.48/NA	0	ND	ND	[87]
	**DW**							20.8(100)	0(0.2)	0.1(0.3)	
								28.5(100)	0.5(2.2)	11.0(79.0)	
17β-estradiol (E2)	GW	Austria	NA	NA	NA	NA	NA	NA	NA	0.79	[75]
	GW	France	2007	NA/3	3	SPE-LC-MS/MS	0.03/NA	67	0.8	1.3	[136]
	GW	USA	2014/2015	GS/6	36	SPE-UPLC-MS/MS	NA	NA	0.98	27.47	[74]
	GW	USA	NA	NA	NA	NA	NA	NA	NA	147	[33]
	**GW**							67.0	0.9(1.0)	44.1(147.0)	
	DW	Canada	2006/2007	GS/17	123	SPE-LC-MS/MS	NA	0	ND	ND	[113]
	DW	France	2007/2008	GS/8	24	SPE-LC-MS/MS	NA/0.06	0	ND	ND	[92]
	DW	USA	2006/2007	GS/19	33	SPE-GC/LC-MS/MS	0.50/NA	0	ND	ND	[127]
	DW	USA	2012/2013	GS/4	27	SPE-UPLC-MS/MS	14.8/NA	0	ND	ND	[87]
	**DW**							0	0	0	

Pharmaceutical	Sample	Country	Year	Sampling type/locations	Number of samples	Method	LOD/LOQ (ng L-1)	Frequency (highest value) (%)	Average (highest value) (ng L-1)	Maximum (highest value) (ng L-1)	Reference
								13.4(67.0)	0.3(1.0)	22.1(147.0)	
17α-estradiol (α-E2)	GW	France	2007	NA/3	3	SPE-LC-MS/MS	0.03/NA	67	1.0	1.6	[136]
	DW	Canada	2006/2007	GS/17	123	SPE-LC-MS/MS	NA	0	ND	ND	[113]
	DW	France	2007/2008	GS/8	24	SPE-LC-MS/MS	NA/0.02	0	ND	ND	[92]
	**DW**							0	0	0	
								22.3(67.0)	0.3(1.0)	0.5(1.6)	
17α-ethinylestradiol (EE2)	GW	Austria	NA	NA	NA	NA	NA	NA	NA	0.94	[75]
	GW	France	2007	NA/3	3	SPE-LC-MS/MS	0.03/NA	67	1.8	3	[136]
	GW	France	NA	NA	NA	NA	NA	NA	NA	3	[75]
	GW	USA	2014/2015	GS/6	36	SPE-UPLC-MS/MS	NA	NA	120.01	4320.35	[74]
	GW	USA	NA	NA	NA	NA	NA	NA	NA	230	[33]
	**GW**							67.0	60.9(120.0)	911.5(4320.4)	
	DW	Canada	2006/2007	GS/17	123	SPE-LC-MS/MS	NA	0	ND	ND	[113]
	DW	France	2007/2008	GS/8	24	SPE-LC-MS/MS	NA/0.4	0	ND	ND	[92]
	DW	USA	2006/2007	GS/19	33	SPE-GC/LC-MS/MS	1.0/NA	0	ND	ND	[127]
	DW	USA	2012/2013	GS/4	27	SPE-UPLC-MS/MS	8.48/NA	0	ND	ND	[87]
	**DW**							0	0	0	
								13.4(67.0)	20.3(120.0)	506.4(4320.4)	
Total								19.6(100)	6.3(120.0)	172.3(4320.4)	

CS - Composite sampling
DW – Drinking water
ESI- Electrospray ionization
FP - Flow proportional sampling
GC - Gas chromatography
GS - Grab sampling

GW - Groundwater
LC - Liquid chromatography
LOQ - Limit of quantification
MS - Mass spectrometry
MinW – Mineral water
NA - Not available
ND - Not detected
SPE - Solid phase extraction
SeaW - Seawater
TP - Time proportional sampling
UPLC - Ultra performance liquid chromatography
UHPLC - Ultra high performance liquid chromatography

References:

[1] L. Vergeynst, A. Haeck, P. De Wispelaere, H. Van Langenhove, K. Demeestere, Multi-residue analysis of pharmaceuticals in wastewater by liquid chromatography-magnetic sector mass spectrometry: Method quality assessment and application in a Belgian case study., Chemosphere. 119 (2015) S2–S8. doi:10.1016/j.chemosphere.2014.03.069.

[2] S. Yuan, X. Jiang, X. Xia, H. Zhang, S. Zheng, Detection, occurrence and fate of 22 psychiatric pharmaceuticals in psychiatric hospital and municipal wastewater treatment plants in Beijing, China., Chemosphere. 90 (2013) 2520–2525. doi:10.1016/j.chemosphere.2012.10.089.

[3] S.-L. YUAN, X.-F. LI, X.-M. JIANG, H.-X. ZHANG, S.-K. ZHENG, Simultaneous determination of 13 psychiatric pharmaceuticals in sewage by automated solid phase extraction and liquid chromatography-mass spectrometry., Chinese J. Anal. Chem. 41 (2013) 49–56. doi:10.1016/S1872-2040(13)60623-4.

[4] M. Wu, J. Xiang, C. Que, F. Chen, G. Xu, Occurrence and fate of psychiatric pharmaceuticals in the urban water system of Shanghai, China, Chemosphere. 138 (2015) 486–493. doi:10.1016/j.chemosphere.2015.07.002.

[5] B. Subedi, K. Balakrishna, D.I. Joshua, K. Kannan, Mass loading and removal of pharmaceuticals and personal care products including psychoactives, antihypertensives, and antibiotics in two sewage treatment plants in southern India, Chemosphere. 167 (2017) 429–437. doi:10.1016/j.chemosphere.2016.10.026.

[6] M.A. Sousa, C. Gonçalves, E. Cunha, J. Hajšlová, M.F. Alpendurada, Cleanup strategies and advantages in the determination of several therapeutic classes of pharmaceuticals in wastewater samples by SPE-LC-MS/MS., Anal. Bioanal. Chem. 399 (2011) 807–22. doi:10.1007/s00216-010-4297-0.

[7] L.H.M.L.M. Santos, M. Gros, S. Rodriguez-Mozaz, C. Delerue-Matos, A. Pena, D. Barceló, M.C.B.S.M. Montenegro, Contribution of hospital effluents to the load of pharmaceuticals in urban wastewaters: identification of ecologically relevant pharmaceuticals., Sci. Total Environ. 461–462 (2013) 302–16. doi:10.1016/j.scitotenv.2013.04.077.

[8] R. Salgado, R. Marques, J.P. Noronha, J.T. Mexia, G. Carvalho, A.

Oehmen, M.A.M. Reis, Assessing the diurnal variability of pharmaceutical and personal care products in a full-scale activated sludge plant., Environ. Pollut. 159 (2011) 2359–2367. doi:10.1016/j.envpol.2011.07.004.

[9] E. Gracia-Lor, J. V. Sancho, R. Serrano, F. Hernández, Occurrence and removal of pharmaceuticals in wastewater treatment plants at the Spanish Mediterranean area of Valencia, Chemosphere. 87 (2012) 453–462. doi:10.1016/j.chemosphere.2011.12.025.

[10] B. Subedi, K. Kannan, Occurrence and fate of select psychoactive pharmaceuticals and antihypertensives in two wastewater treatment plants in New York State, USA., Sci. Total Environ. 514 (2015) 273–280. doi:10.1016/j.scitotenv.2015.01.098.

[11] P. Verlicchi, M. Al Aukidy, E. Zambello, Occurrence of pharmaceutical compounds in urban wastewater: removal, mass load and environmental risk after a secondary treatment-a review., Sci. Total Environ. 429 (2012) 123–155. doi:10.1016/j.scitotenv.2012.04.028.

[12] A. Ginebreda, A. Jelić, M. Petrović, M. López de Alda, D. Barceló, New indexes for compound prioritization and complexity quantification on environmental monitoring inventories., Environ. Sci. Pollut. Res. Int. 19 (2012) 958–70. doi:10.1007/s11356-011-0557-6.

[13] R.H. Lindberg, M. Östman, U. Olofsson, R. Grabic, J. Fick, Occurrence and behaviour of 105 active pharmaceutical ingredients in sewage waters of a municipal sewer collection system., Water Res. 58 (2014) 221–9. doi:10.1016/j.watres.2014.03.076.

[14] M. Gros, M. Petrović, D. Barceló, Development of a multi-residue analytical methodology based on liquid chromatography-tandem mass spectrometry (LC-MS/MS) for screening and trace level determination of pharmaceuticals in surface and wastewaters., Talanta. 70 (2006) 678–90. doi:10.1016/j.talanta.2006.05.024.

[15] O. Golovko, V. Kumar, G. Fedorova, T. Randak, R. Grabic, Seasonal changes in antibiotics, antidepressants/psychiatric drugs, antihistamines and lipid regulators in a wastewater treatment plant., Chemosphere. 111 (2014) 418–26. doi:10.1016/j.chemosphere.2014.03.132.

[16] M.E. Dasenaki, N.S. Thomaidis, Multianalyte method for the determination of pharmaceuticals in wastewater samples using solid-

phase extraction and liquid chromatography-tandem mass spectrometry., Anal. Bioanal. Chem. (2015) 4229–4245. doi:10.1007/s00216-015-8654-x.

[17] P. Verlicchi, M. Al Aukidy, A. Jelic, M. Petrović, D. Barceló, Comparison of measured and predicted concentrations of selected pharmaceuticals in wastewater and surface water: a case study of a catchment area in the Po Valley (Italy)., Sci. Total Environ. 470–471 (2014) 844–54. doi:10.1016/j.scitotenv.2013.10.026.

[18] A. Murata, H. Takada, K. Mutoh, H. Hosoda, A. Harada, N. Nakada, Nationwide monitoring of selected antibiotics: Distribution and sources of sulfonamides, trimethoprim, and macrolides in Japanese rivers., Sci. Total Environ. 409 (2011) 5305–5312. doi:10.1016/j.scitotenv.2011.09.014.

[19] C. Miège, J.M. Choubert, L. Ribeiro, M. Eusèbe, M. Coquery, Fate of pharmaceuticals and personal care products in wastewater treatment plants-conception of a database and first results., Environ. Pollut. 157 (2009) 1721–6. doi:10.1016/j.envpol.2008.11.045.

[20] P. Paíga, L.H.M.L.M. Santos, S. Ramos, S. Jorge, J.G. Silva, C. Delerue-Matos, Presence of pharmaceuticals in the Lis river (Portugal): Sources, fate and seasonal variation., Sci. Total Environ. 573 (2016) 164–177. doi:10.1016/j.scitotenv.2016.08.089.

[21] M. Gros, S. Rodríguez-Mozaz, D. Barceló, Rapid analysis of multiclass antibiotic residues and some of their metabolites in hospital, urban wastewater and river water by ultra-high-performance liquid chromatography coupled to quadrupole-linear ion trap tandem mass spectrometry., J. Chromatogr. A. 1292 (2013) 173–88. doi:10.1016/j.chroma.2012.12.072.

[22] B.D. Blair, J.P. Crago, C.J. Hedman, R.J.F. Treguer, C. Magruder, L.S. Royer, R.D. Klaper, Evaluation of a model for the removal of pharmaceuticals, personal care products, and hormones from wastewater., Sci. Total Environ. 444 (2013) 515–521. doi:10.1016/j.scitotenv.2012.11.103.

[23] E. Zuccato, S. Castiglioni, R. Bagnati, M. Melis, R. Fanelli, Source, occurrence and fate of antibiotics in the Italian aquatic environment., J. Hazard. Mater. 179 (2010) 1042–8. doi:10.1016/j.jhazmat.2010.03.110.

[24] K. Dutta, M.Y. Lee, W.W.P. Lai, C.H. Lee, A.Y.C. Lin, C.F. Lin, J.G. Lin, Removal of pharmaceuticals and organic matter from

municipal wastewater using two-stage anaerobic fluidized membrane bioreactor., Bioresour. Technol. 165 (2014) 42–49. doi:10.1016/j.biortech.2014.03.054.

[25] M. Papageorgiou, C. Kosma, D. Lambropoulou, Seasonal occurrence, removal, mass loading and environmental risk assessment of 55 pharmaceuticals and personal care products in a municipal wastewater treatment plant in Central Greece., Sci. Total Environ. 543 (2016) 547–569. doi:10.1016/j.scitotenv.2015.11.047.

[26] K.O. K'oreje, L. Vergeynst, D. Ombaka, P. De Wispelaere, M. Okoth, H. Van Langenhove, K. Demeestere, Occurrence patterns of pharmaceutical residues in wastewater, surface water and groundwater of Nairobi and Kisumu city, Kenya, Chemosphere. 149 (2016) 238–244. doi:10.1016/j.chemosphere.2016.01.095.

[27] W. Sim, J. Lee, J. Oh, Occurrence and fate of pharmaceuticals in wastewater treatment plants and rivers in Korea., Environ. Pollut. 158 (2010) 1938–47. doi:10.1016/j.envpol.2009.10.036.

[28] W.-J. Sim, J.-W. Lee, E.-S. Lee, S.-K. Shin, S.-R. Hwang, J.-E. Oh, Occurrence and distribution of pharmaceuticals in wastewater from households, livestock farms, hospitals and pharmaceutical manufactures., Chemosphere. 82 (2011) 179–86. doi:10.1016/j.chemosphere.2010.10.026.

[29] M. Seifrtová, A. Pena, C.M. Lino, P. Solich, Determination of fluoroquinolone antibiotics in hospital and municipal wastewaters in Coimbra by liquid chromatography with a monolithic column and fluorescence detection., Anal. Bioanal. Chem. 391 (2008) 799–805. doi:10.1007/s00216-008-2020-1.

[30] R. Rosal, A. Rodríguez, J.A. Perdigón-Melón, A. Petre, E. García-Calvo, M.J. Gómez, A. Agüera, A.R. Fernández-Alba, Occurrence of emerging pollutants in urban wastewater and their removal through biological treatment followed by ozonation., Water Res. 44 (2010) 578–88. doi:10.1016/j.watres.2009.07.004.

[31] R.H. Lindberg, P. Wennberg, M.I. Johansson, M. Tysklind, B.A. V Andersson, Screening of Human Antibiotic Substances and Determination of Weekly Mass Flows in Five Sewage Treatment Plants in Sweden., Environ. Sci. Technol. 39 (2005) 3421–3429. doi:10.1021/es048143z.

[32] K.G. Karthikeyan, M.T. Meyer, Occurrence of antibiotics in wastewater treatment facilities in Wisconsin, USA., Sci. Total

Environ. 361 (2006) 196–207. doi:10.1016/j.scitotenv.2005.06.030.

[33] Y. Luo, W. Guo, H.H. Ngo, L.D. Nghiem, F.I. Hai, J. Zhang, S. Liang, X.C. Wang, A review on the occurrence of micropollutants in the aquatic environment and their fate and removal during wastewater treatment., Sci. Total Environ. 473–474 (2014) 619–641. doi:10.1016/j.scitotenv.2013.12.065.

[34] S. Matongo, G. Birungi, B. Moodley, P. Ndungu, Occurrence of selected pharmaceuticals in water and sediment of Umgeni River, KwaZulu-Natal, South Africa., Environ. Sci. Pollut. Res. 22 (2015) 10298–10308. doi:10.1007/s11356-015-4217-0.

[35] B. Kasprzyk-Hordern, R.M. Dinsdale, A.J. Guwy, The removal of pharmaceuticals, personal care products, endocrine disruptors and illicit drugs during wastewater treatment and its impact on the quality of receiving waters., Water Res. 43 (2009) 363–380. doi:10.1016/j.watres.2008.10.047.

[36] B. Petrie, R. Barden, B. Kasprzyk-Hordern, A review on emerging contaminants in wastewaters and the environment: Current knowledge, understudied areas and recommendations for future monitoring., Water Res. 72 (2014) 3–27. doi:10.1016/j.watres.2014.08.053.

[37] P. Gao, Y. Ding, H. Li, I. Xagoraraki, Occurrence of pharmaceuticals in a municipal wastewater treatment plant: mass balance and removal processes., Chemosphere. 88 (2012) 17–24. doi:10.1016/j.chemosphere.2012.02.017.

[38] M. Stumpf, T.A. Ternes, R.D. Wilken, S. V Rodrigues, W. Baumann, Polar drug residues in sewage and natural waters in the state of Rio de Janeiro, Brazil., Sci. Total Environ. 225 (1999) 135–41. doi:10.1016/S0048-9697(98)00339-8.

[39] Q. Sui, J. Huang, S. Deng, W. Chen, G. Yu, Seasonal variation in the occurrence and removal of pharmaceuticals and personal care products in different biological wastewater treatment processes., Environ. Sci. Technol. 45 (2011) 3341–8. doi:10.1021/es200248d.

[40] C.I. Kosma, D.A. Lambropoulou, T.A. Albanis, Investigation of PPCPs in wastewater treatment plants in Greece: Occurrence, removal and environmental risk assessment., Sci. Total Environ. 466–467 (2014) 421–438. doi:10.1016/j.scitotenv.2013.07.044.

[41] J.A. Rivera-Jaimes, C. Postigo, R.M. Melgoza-Alemán, J. Aceña, D.

Barceló, M. López de Alda, Study of pharmaceuticals in surface and wastewater from Cuernavaca, Morelos, Mexico: Occurrence and environmental risk assessment, Sci. Total Environ. 613–614 (2018) 1263–1274. doi:10.1016/j.scitotenv.2017.09.134.

[42] E. Carmona, V. Andreu, Y. Picó, Occurrence of acidic pharmaceuticals and personal care products in Turia River Basin: from waste to drinking water., Sci. Total Environ. 484 (2014) 53–63. doi:10.1016/j.scitotenv.2014.02.085.

[43] D.R. Baker, B. Kasprzyk-Hordern, Spatial and temporal occurrence of pharmaceuticals and illicit drugs in the aqueous environment and during wastewater treatment: New developments., Sci. Total Environ. 454–455 (2013) 442–456. doi:10.1016/j.scitotenv.2013.03.043.

[44] H.-B. Lee, T.E. Peart, M.L. Svoboda, Determination of endocrine-disrupting phenols, acidic pharmaceuticals, and personal-care products in sewage by solid-phase extraction and gas chromatography-mass spectrometry., J. Chromatogr. A. 1094 (2005) 122–9. doi:10.1016/j.chroma.2005.07.070.

[45] Q. Sun, M. Lv, A. Hu, X. Yang, C.-P. Yu, Seasonal variation in the occurrence and removal of pharmaceuticals and personal care products in a wastewater treatment plant in Xiamen, China., J. Hazard. Mater. (2013). doi:10.1016/j.jhazmat.2013.11.056.

[46] Q. Sun, M. Li, C. Ma, X. Chen, X. Xie, C.P. Yu, Seasonal and spatial variations of PPCP occurrence, removal and mass loading in three wastewater treatment plants located in different urbanization areas in Xiamen, China., Environ. Pollut. 208 (2016) 371–381. doi:10.1016/j.envpol.2015.10.003.

[47] S.K. Behera, H.W. Kim, J.-E. Oh, H.-S. Park, Occurrence and removal of antibiotics, hormones and several other pharmaceuticals in wastewater treatment plants of the largest industrial city of Korea., Sci. Total Environ. 409 (2011) 4351–60. doi:10.1016/j.scitotenv.2011.07.015.

[48] D. Bendz, N. a Paxéus, T.R. Ginn, F.J. Loge, Occurrence and fate of pharmaceutically active compounds in the environment, a case study: Höje River in Sweden., J. Hazard. Mater. 122 (2005) 195–204. doi:10.1016/j.jhazmat.2005.03.012.

[49] X.-S. Miao, C.D. Metcalfe, Determination of cholesterol-lowering statin drugs in aqueous samples using liquid chromatography–electrospray ionization tandem mass spectrometry., J. Chromatogr. A.

998 (2003) 133–141. doi:10.1016/S0021-9673(03)00645-9.

[50] F.F. Al-Qaim, M.P. Abdullah, M.R. Othman, J. Latip, Z. Zakaria, Multi-residue analytical methodology-based liquid chromatography-time-of-flight-mass spectrometry for the analysis of pharmaceutical residues in surface water and effluents from sewage treatment plants and hospitals., J. Chromatogr. A. 1345 (2014) 139–53. doi:10.1016/j.chroma.2014.04.025.

[51] M. Boisvert, P.B. Fayad, S. Sauvé, Development of a new multi-residue laser diode thermal desorption atmospheric pressure chemical ionization tandem mass spectrometry method for the detection and quantification of pesticides and pharmaceuticals in wastewater samples., Anal. Chim. Acta. 754 (2012) 75–82. doi:10.1016/j.aca.2012.09.046.

[52] N. Nakada, T. Tanishima, H. Shinohara, K. Kiri, H. Takada, Pharmaceutical chemicals and endocrine disrupters in municipal wastewater in Tokyo and their removal during activated sludge treatment., Water Res. 40 (2006) 3297–303. doi:10.1016/j.watres.2006.06.039.

[53] R. Salgado, J.P. Noronha, A. Oehmen, G. Carvalho, M.A.M. Reis, Analysis of 65 pharmaceuticals and personal care products in 5 wastewater treatment plants in Portugal using a simplified analytical methodology., Water Sci. Technol. 62 (2010) 2862. doi:10.2166/wst.2010.985.

[54] M.J. Gómez, M.J. Martínez Bueno, S. Lacorte, A.R. Fernández-Alba, A. Agüera, Pilot survey monitoring pharmaceuticals and related compounds in a sewage treatment plant located on the Mediterranean coast., Chemosphere. 66 (2007) 993–1002. doi:10.1016/j.chemosphere.2006.07.051.

[55] A. Lajeunesse, C. Gagnon, S. Sauvé, Determination of Basic Antidepressants and Their N-Desmethyl Metabolites in Raw Sewage and Wastewater Using Solid-Phase Extraction and Liquid Chromatography-Tandem Mass Spectrometry., Anal. Chem. 80 (2008) 5325–5333. doi:10.1021/ac800162q.

[56] T. Vasskog, U. Berger, P.-J. Samuelsen, R. Kallenborn, E. Jensen, Selective serotonin reuptake inhibitors in sewage influents and effluents from Tromsø, Norway., J. Chromatogr. A. 1115 (2006) 187–195. doi:10.1016/j.chroma.2006.02.091.

[57] T. Vasskog, T. Anderssen, S. Pedersen-Bjergaard, R. Kallenborn, E.

Jensen, Occurrence of selective serotonin reuptake inhibitors in sewage and receiving waters at Spitsbergen and in Norway., J. Chromatogr. A. 1185 (2008) 194–205. doi:10.1016/j.chroma.2008.01.063.

[58] V. Koutsouba, T. Heberer, B. Fuhrmann, K. Schmidt-Baumler, D. Tsipi, A. Hiskia, Determination of polar pharmaceuticals in sewage water of Greece by gas chromatography-mass spectrometry., Chemosphere. 51 (2003) 69–75. doi:10.1016/S0045-6535(02)00819-6.

[59] V.G. Samaras, A.S. Stasinakis, D. Mamais, N.S. Thomaidis, T.D. Lekkas, Fate of selected pharmaceuticals and synthetic endocrine disrupting compounds during wastewater treatment and sludge anaerobic digestion., J. Hazard. Mater. 244–245 (2013) 259–267. doi:10.1016/j.jhazmat.2012.11.039.

[60] J.-Y. Pailler, A. Krein, L. Pfister, L. Hoffmann, C. Guignard, Solid phase extraction coupled to liquid chromatography-tandem mass spectrometry analysis of sulfonamides, tetracyclines, analgesics and hormones in surface water and wastewater in Luxembourg., Sci. Total Environ. 407 (2009) 4736–4743. doi:10.1016/j.scitotenv.2009.04.042.

[61] L.H.M.L.M. Santos, A.N. Araújo, A. Fachini, A. Pena, C. Delerue-Matos, M.C.B.S.M. Montenegro, Ecotoxicological aspects related to the presence of pharmaceuticals in the aquatic environment., J. Hazard. Mater. 175 (2010) 45–95. doi:10.1016/j.jhazmat.2009.10.100.

[62] M.D. Hernando, E. Heath, M. Petrovic, D. Barceló, Trace-level determination of pharmaceutical residues by LC-MS/MS in natural and treated waters. A pilot-survey study., Anal. Bioanal. Chem. 385 (2006) 985–91. doi:10.1007/s00216-006-0394-5.

[63] A. Tauxe-Wuersch, L.F. De Alencastro, D. Grandjean, J. Tarradellas, Occurrence of several acidic drugs in sewage treatment plants in Switzerland and risk assessment., Water Res. 39 (2005) 1761–72. doi:10.1016/j.watres.2005.03.003.

[64] L. Ferrando-Climent, N. Collado, G. Buttiglieri, M. Gros, I. Rodriguez-Roda, S. Rodriguez-Mozaz, D. Barceló, Comprehensive study of ibuprofen and its metabolites in activated sludge batch experiments and aquatic environment., Sci. Total Environ. 438 (2012) 404–13. doi:10.1016/j.scitotenv.2012.08.073.

[65] S. Zuehlke, U. Duennbier, T. Heberer, Determination of estrogenic steroids in surface water and wastewater by liquid chromatography-electrospray tandem mass spectrometry., J. Sep. Sci. 28 (2005) 52–58. doi:10.1002/jssc.200301727.

[66] A.C. Johnson, R.J. Williams, A Model To Estimate Influent and Effluent Concentrations of Estradiol, Estrone, and Ethinylestradiol at Sewage Treatment Works., Environ. Sci. Technol. 38 (2004) 3649–3658. doi:10.1021/es035342u.

[67] A. Laganà, A. Bacaloni, I. De Leva, A. Faberi, G. Fago, A. Marino, Analytical methodologies for determining the occurrence of endocrine disrupting chemicals in sewage treatment plants and natural waters., Anal. Chim. Acta. 501 (2004) 79–88. doi:10.1016/j.aca.2003.09.020.

[68] R. Loos, R. Carvalho, D.C. António, S. Comero, G. Locoro, S. Tavazzi, B. Paracchini, M. Ghiani, T. Lettieri, L. Blaha, B. Jarosova, S. Voorspoels, K. Servaes, P. Haglund, J. Fick, R.H. Lindberg, D. Schwesig, B.M. Gawlik, EU-wide monitoring survey on emerging polar organic contaminants in wastewater treatment plant effluents., Water Res. 47 (2013) 6475–87. doi:10.1016/j.watres.2013.08.024.

[69] M.S. Kostich, A.L. Batt, J.M. Lazorchak, Concentrations of prioritized pharmaceuticals in effluents from 50 large wastewater treatment plants in the US and implications for risk estimation., Environ. Pollut. 184 (2014) 354–359. doi:10.1016/j.envpol.2013.09.013.

[70] P. Verlicchi, A. Galletti, M. Petrovic, D. Barceló, M. Al Aukidy, E. Zambello, Removal of selected pharmaceuticals from domestic wastewater in an activated sludge system followed by a horizontal subsurface flow bed - Analysis of their respective contributions., Sci. Total Environ. 454–455 (2013) 411–425. doi:10.1016/j.scitotenv.2013.03.044.

[71] M. Al Aukidy, P. Verlicchi, A. Jelic, M. Petrovic, D. Barcelò, Monitoring release of pharmaceutical compounds: Occurrence and environmental risk assessment of two WWTP effluents and their receiving bodies in the Po Valley, Italy., Sci. Total Environ. 438 (2012) 15–25. doi:10.1016/j.scitotenv.2012.08.061.

[72] S. Grujić, T. Vasiljević, M. Laušević, Determination of multiple pharmaceutical classes in surface and ground waters by liquid chromatography-ion trap-tandem mass spectrometry., J. Chromatogr. A. 1216 (2009) 4989–5000. doi:10.1016/j.chroma.2009.04.059.

[73] M.J.M. Bueno, M.J. Gomez, S. Herrera, M.D. Hernando, A. Agüera, A.R. Fernández-Alba, Occurrence and persistence of organic emerging contaminants and priority pollutants in five sewage treatment plants of Spain: two years pilot survey monitoring., Environ. Pollut. 164 (2012) 267–73. doi:10.1016/j.envpol.2012.01.038.

[74] A.D. McEachran, D. Shea, W. Bodnar, E.G. Nichols, Pharmaceutical occurrence in groundwater and surface waters in forests land-applied with municipal wastewater., Environ. Toxicol. Chem. 35 (2016) 898–905. doi:10.1002/etc.3216.

[75] W.C. Li, Occurrence, sources, and fate of pharmaceuticals in aquatic environment and soil., Environ. Pollut. 187C (2014) 193–201. doi:10.1016/j.envpol.2014.01.015.

[76] A. Chiffre, F. Degiorgi, A. Buleté, L. Spinner, P.M. Badot, Occurrence of pharmaceuticals in WWTP effluents and their impact in a karstic rural catchment of Eastern France, Environ. Sci. Pollut. Res. 23 (2016) 25427–25441. doi:10.1007/s11356-016-7751-5.

[77] J. Fick, H. Söderström, Contamination of surface, ground, and drinking water from pharmaceutical production., Environ. Toxicol. Chem. 28 (2009) 2522–2527. http://en.cnki.com.cn/Article_en/CJFDTOTAL-HXJZ2009Z1013.htm (accessed November 5, 2013).

[78] L. Mijangos, H. Ziarrusta, O. Ros, L. Kortazar, L.A. Fernández, M. Olivares, O. Zuloaga, A. Prieto, N. Etxebarria, Occurrence of emerging pollutants in estuaries of the Basque Country: Analysis of sources and distribution, and assessment of the environmental risk, Water Res. 147 (2018) 152–163. doi:10.1016/j.watres.2018.09.033.

[79] S.D. Kim, J. Cho, I.S. Kim, B.J. Vanderford, S.A. Snyder, Occurrence and removal of pharmaceuticals and endocrine disruptors in South Korean surface, drinking, and waste waters., Water Res. 41 (2007) 1013–1021. doi:10.1016/j.watres.2006.06.034.

[80] C. Nebot, R. Falcon, K.G. Boyd, S.W. Gibb, Introduction of human pharmaceuticals from wastewater treatment plants into the aquatic environment: a rural perspective., Environ. Sci. Pollut. Res. 14 (2015) 10559–10568. doi:10.1007/s11356-015-4234-z.

[81] M.J. Hilton, K. V Thomas, Determination of selected human pharmaceutical compounds in effluent and surface water samples by high-performance liquid chromatography–electrospray tandem mass

spectrometry., J. Chromatogr. A. 1015 (2003) 129–141. doi:10.1016/S0021-9673(03)01213-5.

[82] M.J.M. Bueno, A. Agüera, M.D. Hernando, M.J. Gómez, A.R. Fernández-Alba, Evaluation of various liquid chromatography-quadrupole-linear ion trap-mass spectrometry operation modes applied to the analysis of organic pollutants in wastewaters., J. Chromatogr. A. 1216 (2009) 5995–6002. doi:10.1016/j.chroma.2009.06.047.

[83] S.S. Verenitch, C.J. Lowe, A. Mazumder, Determination of acidic drugs and caffeine in municipal wastewaters and receiving waters by gas chromatography-ion trap tandem mass spectrometry., J. Chromatogr. A. 1116 (2006) 193–203. doi:10.1016/j.chroma.2006.03.005.

[84] G. McEneff, L. Barron, B. Kelleher, B. Paull, B. Quinn, A year-long study of the spatial occurrence and relative distribution of pharmaceutical residues in sewage effluent, receiving marine waters and marine bivalves., Sci. Total Environ. 476–477 (2014) 317–326. doi:10.1016/j.scitotenv.2013.12.123.

[85] J. Corcoran, M.J. Winter, C.R. Tyler, Pharmaceuticals in the aquatic environment: a critical review of the evidence for health effects in fish., Crit. Rev. Toxicol. 40 (2010) 287–304. doi:10.3109/10408440903373590.

[86] A. Daneshvar, J. Svanfelt, L. Kronberg, M. Prévost, G.A. Weyhenmeyer, Seasonal variations in the occurrence and fate of basic and neutral pharmaceuticals in a Swedish river-lake system., Chemosphere. 80 (2010) 301–9. doi:10.1016/j.chemosphere.2010.03.060.

[87] B. Subedi, N. Codru, D.M. Dziewulski, L.R. Wilson, J. Xue, S. Yun, E. Braun-Howland, C. Minihane, K. Kannan, A pilot study on the assessment of trace organic contaminants including pharmaceuticals and personal care products from on-site wastewater treatment systems along Skaneateles Lake in New York State, USA., Water Res. 72 (2014) 28–39. doi:10.1016/j.watres.2014.10.049.

[88] B.A. Crouse, A.J. Ghoshdastidar, A.Z. Tong, The presence of acidic and neutral drugs in treated sewage effluents and receiving waters in the Cornwallis and Annapolis River watersheds and the Mill CoveSewage Treatment Plant in Nova Scotia, Canada., Environ. Res. 112 (2012) 92–9. doi:10.1016/j.envres.2011.11.011.

[89] T. Isobe, H. Shiraishi, M. Yasuda, A. Shinoda, H. Suzuki, M. Morita, Determination of estrogens and their conjugates in water using solid-phase extraction followed by liquid chromatography-tandem mass spectrometry., J. Chromatogr. A. 984 (2003) 195–202. doi:10.1016/S0021-9673(02)01851-4.

[90] C.M.O. Gonçalves, M.A.D. Sousa, M. de F.P.S.P. Alpendurada, Analysis of acidic, basic and neutral pharmaceuticals in river waters: Clean-up by 1°, 2° amino anion exchange and enrichment using an hydrophilic adsorbent, Int. J. Environ. Anal. Chem. 93 (2013) 1–22. doi:10.1080/03067319.2012.702272.

[91] P. Reis-Santos, M. Pais, B. Duarte, I. Caçador, A. Freitas, A.S. Vila Pouca, J. Barbosa, S. Leston, J. Rosa, F. Ramos, H.N. Cabral, B.M. Gillanders, V.F. Fonseca, Screening of human and veterinary pharmaceuticals in estuarine waters: A baseline assessment for the Tejo estuary, Mar. Pollut. Bull. 135 (2018) 1079–1084. doi:10.1016/j.marpolbul.2018.08.036.

[92] E. Vulliet, C. Cren-Olivé, M.-F. Grenier-Loustalot, Occurrence of pharmaceuticals and hormones in drinking water treated from surface waters., Environ. Chem. Lett. 9 (2009) 103–114. doi:10.1007/s10311-009-0253-7.

[93] R. López-Serna, M. Petrović, D. Barceló, Direct analysis of pharmaceuticals, their metabolites and transformation products in environmental waters using on-line TurboFlow™ chromatography-liquid chromatography-tandem mass spectrometry., J. Chromatogr. A. 1252 (2012) 115–29. doi:10.1016/j.chroma.2012.06.078.

[94] B. Huerta, S. Rodriguez-Mozaz, C. Nannou, L. Nakis, A. Ruhí, V. Acuña, S. Sabater, D. Barcelo, Determination of a broad spectrum of pharmaceuticals and endocrine disruptors in biofilm from a waste water treatment plant-impacted river., Sci. Total Environ. 540 (2016) 241–249. doi:10.1016/j.scitotenv.2015.05.049.

[95] R. Zhang, G. Zhang, Q. Zheng, J. Tang, Y. Chen, W. Xu, Y. Zou, X. Chen, Occurrence and risks of antibiotics in the Laizhou Bay, China: Impacts of river discharge., Ecotoxicol. Environ. Saf. 80 (2012) 208–215. doi:10.1016/j.ecoenv.2012.03.002.

[96] Q. Bu, B. Wang, J. Huang, S. Deng, G. Yu, Pharmaceuticals and personal care products in the aquatic environment in China: A review., J. Hazard. Mater. 262 (2013) 189–211. doi:10.1016/j.jhazmat.2013.08.040.

[97] M. Ivešić, A. Krivohlavek, I. Žuntar, S. Tolić, S. Šikić, V. Music, I. Pavlić, A. Bursik, N. Galić, Monitoring of selected pharmaceuticals in surface waters of Croatia, Environ. Sci. Pollut. Res. 24 (2017) 23389–23400. doi:10.1007/s11356-017-9894-4.

[98] T. Christian, R.J. Schneider, H.A. Färber, D. Skutlarek, M.T. Meyer, H.E. Goldbach, Determination of antibiotic residues in manure, soil, and surface waters., Acta Hydrochim. Hydrobiol. 31 (2003) 36–44. doi:10.1002/aheh.200390014.

[99] P. a. Segura, H. Takada, J. a. Correa, K. El Saadi, T. Koike, S. Onwona-Agyeman, J. Ofosu-Anim, E.B. Sabi, O. V. Wasonga, J.M. Mghalu, A.M. dos Santos, B. Newman, S. Weerts, V. Yargeau, Global occurrence of anti-infectives in contaminated surface waters: Impact of income inequality between countries., Environ. Int. 80 (2015) 89–97. doi:10.1016/j.envint.2015.04.001.

[100] S.R. Hughes, L.E. Brown, P. Kay, Global Synthesis and Critical Evaluation of Pharmaceutical Data Sets Collected from River Systems., Environ. Sci. Technol. 47 (2013) 661–677. http://pubs.acs.org/doi/abs/10.1021/es3030148.

[101] S. Managaki, A. Murata, H. Takada, C.T. Bui, N.H. Chiem, Distribution of macrolides, sulfonamides, and trimethoprim in tropical waters: Ubiquitous occurrence of veterinary antibiotics in the Mekong Delta., Environ. Sci. Technol. 41 (2007) 8004–8010. doi:10.1021/es0709021.

[102] G.A. Khan, B. Berglund, K.M. Khan, P.E. Lindgren, J. Fick, Occurrence and Abundance of Antibiotics and Resistance Genes in Rivers, Canal and near Drug Formulation Facilities - A Study in Pakistan., PLoS One. 8 (2013) 4–11. doi:10.1371/journal.pone.0062712.

[103] A. Ginebreda, I. Muñoz, M.L. Alda, R. Brix, J. López-Doval, D. Barceló, Environmental risk assessment of pharmaceuticals in rivers: Relationships between hazard indexes and aquatic macroinvertebrate diversity indexes in the Llobregat River (NE Spain)., Environ. Int. 36 (2010) 153–162. doi:10.1016/j.envint.2009.10.003.

[104] M.J. Focazio, D.W. Kolpin, K.K. Barnes, E.T. Furlong, M.T. Meyer, S.D. Zaugg, L.B. Barber, M.E. Thurman, A national reconnaissance for pharmaceuticals and other organic wastewater contaminants in the United States - II) Untreated drinking water sources., Sci. Total Environ. 402 (2008) 201–216. doi:10.1016/j.scitotenv.2008.02.021.

[105] T.L. Jones-Lepp, C. Sanchez, D.A. Alvarez, D.C. Wilson, R.L. Taniguchi-Fu, Point sources of emerging contaminants along the Colorado River Basin: Source water for the arid Southwestern United States., Sci. Total Environ. 430 (2012) 237–245. doi:10.1016/j.scitotenv.2012.04.053.

[106] B.D. Blair, J.P. Crago, C.J. Hedman, R.D. Klaper, Pharmaceuticals and personal care products found in the Great Lakes above concentrations of environmental concern., Chemosphere. 93 (2013) 2116–2123. doi:10.1016/j.chemosphere.2013.07.057.

[107] P.T.P. Hoa, S. Managaki, N. Nakada, H. Takada, A. Shimizu, D.H. Anh, P.H. Viet, S. Suzuki, Antibiotic contamination and occurrence of antibiotic-resistant bacteria in aquatic environments of northern Vietnam., Sci. Total Environ. 409 (2011) 2894–2901. doi:10.1016/j.scitotenv.2011.04.030.

[108] C. Wu, X. Huang, J.D. Witter, A.L. Spongberg, K. Wang, D. Wang, J. Liu, Occurrence of pharmaceuticals and personal care products and associated environmental risks in the central and lower Yangtze river, China., Ecotoxicol. Environ. Saf. 106 (2014) 19–26. doi:10.1016/j.ecoenv.2014.04.029.

[109] D. Calamari, E. Zuccato, S. Castiglioni, R. Bagnati, R. Fanelli, Strategic Survey of Therapeutic Drugs in the Rivers Po and Lambro in Northern Italy, Environ. Sci. Technol. 37 (2003) 1241–1248. doi:10.1021/es020158e.

[110] J.-W. Kim, H.-S. Jang, J.-G. Kim, H. Ishibashi, M. Hirano, K. Nasu, N. Ichikawa, Y. Takao, R. Shinohara, K. Arizono, Occurrence of Pharmaceutical and Personal Care Products (PPCPs) in Surface Water from Mankyung River, South Korea., J. Heal. Sci. 55 (2009) 249–258. doi:10.1248/jhs.55.249.

[111] T.W. Na, T.W. Kang, K.H. Lee, S.H. Hwang, H.J. Jung, K. Kim, Distribution and ecological risk of pharmaceuticals in surface water of the Yeongsan river, Republic of Korea, Ecotoxicol. Environ. Saf. 181 (2019) 180–186. doi:10.1016/j.ecoenv.2019.06.004.

[112] V. de J. Gaffney, C.M.M. Almeida, A. Rodrigues, E. Ferreira, M.J. Benoliel, V.V. Cardoso, Occurrence of pharmaceuticals in a water supply system and related human health risk assessment., Water Res. 72 (2015) 199–208. doi:10.1016/j.watres.2014.10.027.

[113] S. Kleywegt, V. Pileggi, P. Yang, C. Hao, X. Zhao, C. Rocks, S. Thach, P. Cheung, B. Whitehead, Pharmaceuticals, hormones and

bisphenol A in untreated source and finished drinking water in Ontario, Canada — occurrence and treatment efficiency., Sci. Total Environ. 409 (2011) 1481–8. doi:10.1016/j.scitotenv.2011.01.010.

[114] S. Mokh, M. El Khatib, M. Koubar, Z. Daher, M. Al Iskandarani, Innovative SPE-LC-MS/MS technique for the assessment of 63 pharmaceuticals and the detection of antibiotic-resistant-bacteria: A case study natural water sources in Lebanon, Sci. Total Environ. 609 (2017) 830–841. doi:10.1016/j.scitotenv.2017.07.230.

[115] A. Pena, D. Chmielova, C.M. Lino, P. Solich, Determination of fluoroquinolone antibiotics in surface waters from Mondego River by high performance liquid chromatography using a monolithic column, J. Sep. Sci. 30 (2007) 2924–2928. doi:10.1002/jssc.200700363.

[116] D.W. Kolpin, E.T. Furlong, M.T. Meyer, E.M. Thurman, S.D. Zaugg, L.B. Barber, H.T. Buxton, Pharmaceuticals, hormones, and other organic wastewater contaminants in U.S. streams, 1999-2000: A national reconnaissance., Environ. Sci. Technol. 36 (2002) 1202–1211. doi:10.1021/es011055j.

[117] S. Mompelat, B. Le Bot, O. Thomas, Occurrence and fate of pharmaceutical products and by-products, from resource to drinking water., Environ. Int. 35 (2009) 803–14. doi:10.1016/j.envint.2008.10.008.

[118] D. Simazaki, R. Kubota, T. Suzuki, M. Akiba, T. Nishimura, S. Kunikane, Occurrence of selected pharmaceuticals at drinking water purification plants in Japan and implications for human health., Water Res. 76 (2015) 187–200. doi:10.1016/j.watres.2015.02.059.

[119] C.M. de Jongh, P.J.F. Kooij, P. de Voogt, T.L. ter Laak, Screening and human health risk assessment of pharmaceuticals and their transformation products in Dutch surface waters and drinking water., Sci. Total Environ. 427–428 (2012) 70–7. doi:10.1016/j.scitotenv.2012.04.010.

[120] R. López-Roldán, M.L. de Alda, M. Gros, M. Petrovic, J. Martín-Alonso, D. Barceló, Advanced monitoring of pharmaceuticals and estrogens in the Llobregat River basin (Spain) by liquid chromatography-triple quadrupole-tandem mass spectrometry in combination with ultra performance liquid chromatography-time of flight-mass spectrometry., Chemosphere. 80 (2010) 1337–44. doi:10.1016/j.chemosphere.2010.06.042.

[121] A. Ginebreda, M. Kuzmanovic, H. Guasch, M.L. de Alda, J.C.

López-Doval, I. Muñoz, M. Ricart, A.M. Romaní, S. Sabater, D. Barceló, Assessment of multi-chemical pollution in aquatic ecosystems using toxic units: compound prioritization, mixture characterization and relationships with biological descriptors., Sci. Total Environ. 468–469 (2014) 715–723. doi:10.1016/j.scitotenv.2013.08.086.

[122] A.Y.C. Lin, Y.T. Tsai, Occurrence of pharmaceuticals in Taiwan's surface waters: Impact of waste streams from hospitals and pharmaceutical production facilities., Sci. Total Environ. 407 (2009) 3793–3802. doi:10.1016/j.scitotenv.2009.03.009.

[123] U. Kunkel, M. Radke, Fate of pharmaceuticals in rivers: Deriving a benchmark dataset at favorable attenuation conditions., Water Res. 46 (2012) 5551–5565. doi:10.1016/j.watres.2012.07.033.

[124] Z. Li, A. Sobek, M. Radke, Fate of pharmaceuticals and their transformation products in four small european rivers receiving treated wastewater., Environ. Sci. Technol. (2016) acs.est.5b06327. doi:10.1021/acs.est.5b06327.

[125] J.L. Zhao, G.G. Ying, L. Wang, J.F. Yang, X.B. Yang, L.H. Yang, X. Li, Determination of phenolic endocrine disrupting chemicals and acidic pharmaceuticals in surface water of the Pearl Rivers in South China by gas chromatography-negative chemical ionization-mass spectrometry., Sci. Total Environ. 407 (2009) 962–974. doi:10.1016/j.scitotenv.2008.09.048.

[126] M. Lv, Q. Sun, A. Hu, L. Hou, J. Li, X. Cai, C.-P. Yu, Pharmaceuticals and personal care products in a mesoscale subtropical watershed and their application as sewage markers., J. Hazard. Mater. 280 (2014) 696–705. doi:10.1016/j.jhazmat.2014.08.054.

[127] M.J. Benotti, R.A. Trenholm, B.J. Vanderford, J.C. Holady, B.D. Stanford, S.A. Snyder, Pharmaceuticals and endocrine disrupting compounds in U.S. drinking water., Environ. Sci. Technol. 43 (2009) 597–603. doi:10.1021/es801845a.

[128] M.G. Cantwell, D.R. Katz, J.C. Sullivan, D. Shapley, J. Lipscomb, J. Epstein, A.R. Juhl, C. Knudson, G.D. O'Mullan, Spatial patterns of pharmaceuticals and wastewater tracers in the Hudson River Estuary, Water Res. 137 (2018) 335–343. doi:10.1016/j.watres.2017.12.044.

[129] A. Hossain, S. Nakamichi, M. Habibullah-Al-Mamun, K. Tani, S. Masunaga, H. Matsuda, Occurrence and ecological risk of

pharmaceuticals in river surface water of Bangladesh, Environ. Res. 165 (2018) 258–266. doi:10.1016/j.envres.2018.04.030.

[130] M. Schriks, M.B. Heringa, M.M.E. Van der Kooi, P. de Voogt, A.P. Van Wezel, Toxicological relevance of emerging contaminants for drinking water quality., Water Res. 44 (2010) 461–76. doi:10.1016/j.watres.2009.08.023.

[131] T.V. Madureira, J.C. Barreiro, M.J. Rocha, E. Rocha, Q.B. Cass, M.E. Tiritan, Spatiotemporal distribution of pharmaceuticals in the Douro River estuary (Portugal)., Sci. Total Environ. 408 (2010) 5513–20. doi:10.1016/j.scitotenv.2010.07.069.

[132] S. Weigel, R. Kallenborn, H. Hühnerfuss, Simultaneous solid-phase extraction of acidic, neutral and basic pharmaceuticals from aqueous samples at ambient (neutral) pH and their determination by gas chromatography-mass spectrometry., J. Chromatogr. A. 1023 (2004) 183–195. doi:10.1016/j.chroma.2003.10.036.

[133] B.P. Gumbi, B. Moodley, G. Birungi, P.G. Ndungu, Detection and quantification of acidic drug residues in South African surface water using gas chromatography-mass spectrometry, Chemosphere. 168 (2017) 1042–1050. doi:10.1016/j.chemosphere.2016.10.105.

[134] L.H.M.L.M. Santos, P. Paíga, A.N. Araújo, A. Pena, C. Delerue-Matos, M.C.B.S.M. Montenegro, Development of a simple analytical method for the simultaneous determination of paracetamol, paracetamol-glucuronide and p-aminophenol in river water., J. Chromatogr. B. Analyt. Technol. Biomed. Life Sci. 930 (2013) 75–81. doi:10.1016/j.jchromb.2013.04.032.

[135] L. Yang, T. Luan, C. Lan, Solid-phase microextraction with on-fiber silylation for simultaneous determinations of endocrine disrupting chemicals and steroid hormones by gas chromatography-mass spectrometry., J. Chromatogr. A. 1104 (2006) 23–32. doi:10.1016/j.chroma.2005.11.108.

[136] E. Vulliet, L. Wiest, R. Baudot, M.F. Grenier-Loustalot, Multi-residue analysis of steroids at sub-ng/L levels in surface and ground-waters using liquid chromatography coupled to tandem mass spectrometry., J. Chromatogr. A. 1210 (2008) 84–91. doi:10.1016/j.chroma.2008.09.034.

[137] O.S. Olatunji, O.S. Fatoki, B.O. Opeolu, B.J. Ximba, R. Chitongo, Determination of selected steroid hormones in some surface water around animal farms in Cape Town using HPLC-DAD, Environ.

Monit. Assess. 189 (2017). doi:10.1007/s10661-017-6070-8.

[138] R. López-Serna, A. Jurado, E. Vázquez-Suñé, J. Carrera, M. Petrović, D. Barceló, Occurrence of 95 pharmaceuticals and transformation products in urban groundwaters underlying the metropolis of Barcelona, Spain., Environ. Pollut. 174 (2013) 305–315. doi:10.1016/j.envpol.2012.11.022.

[139] C. Afonso-Olivares, M. Torres-Padrón, Z. Sosa-Ferrera, J. Santana-Rodríguez, Assessment of the Presence of Pharmaceutical Compounds in Seawater Samples from Coastal Area of Gran Canaria Island (Spain)., Antibiotics. 2 (2013) 274–287. doi:10.3390/antibiotics2020274.

[140] K.K. Barnes, D.W. Kolpin, E.T. Furlong, S.D. Zaugg, M.T. Meyer, L.B. Barber, A national reconnaissance of pharmaceuticals and other organic wastewater contaminants in the United States — I) Groundwater., Sci. Total Environ. 402 (2008) 192–200. doi:10.1016/j.scitotenv.2008.04.028.

[141] J. Magnér, M. Filipovic, T. Alsberg, Application of a novel solid-phase-extraction sampler and ultra-performance liquid chromatography quadrupole-time-of-flight mass spectrometry for determination of pharmaceutical residues in surface sea water., Chemosphere. 80 (2010) 1255–60. doi:10.1016/j.chemosphere.2010.06.065.

[142] F. Sacher, F.T. Lange, H.-J. Brauch, I. Blankenhorn, Pharmaceuticals in groundwaters., J. Chromatogr. A. 938 (2001) 199–210. doi:10.1016/S0021-9673(01)01266-3.

[143] M.S. Fram, K. Belitz, Occurrence and concentrations of pharmaceutical compounds in groundwater used for public drinking-water supply in California., Sci. Total Environ. 409 (2011) 3409–17. doi:10.1016/j.scitotenv.2011.05.053.

[144] F. Riva, S. Castiglioni, E. Fattore, A. Manenti, E. Davoli, E. Zuccato, Monitoring emerging contaminants in the drinking water of Milan and assessment of the human risk, Int. J. Hyg. Environ. Health. 221 (2018) 451–457. doi:10.1016/j.ijheh.2018.01.008.
